# Effect of fentanyl on HIV expression in peripheral blood mononuclear cells

**DOI:** 10.3389/fmicb.2024.1463441

**Published:** 2024-09-25

**Authors:** Janani Madhuravasal Krishnan, Krishna M. Roskin, Heidi L. Meeds, Jason T. Blackard

**Affiliations:** ^1^Division of Digestive Diseases, Department of Internal Medicine, University of Cincinnati College of Medicine, Cincinnati, OH, United States; ^2^Divisions of Biomedical Informatics and Immunobiology, Cincinnati Children’s Hospital Medical Center, Cincinnati, OH, United States; ^3^Department of Pediatrics, University of Cincinnati College of Medicine, Cincinnati, OH, United States; ^4^Center for Addiction Research, University of Cincinnati College of Medicine, Cincinnati, OH, United States

**Keywords:** opioid, fentanyl, drug use, HIV, CD4^+^ T lymphocytes, PBMC, single-cell RNA sequencing

## Abstract

**Introduction:**

Illicit drug use, particularly the synthetic opioid fentanyl, presents a significant global health challenge. Previous studies have shown that fentanyl enhances viral replication; yet, the mechanisms by which it affects HIV pathogenesis remain unclear. This study investigated the impact of fentanyl on HIV replication in CD4^+^ T lymphocytes.

**Methods:**

CD4^+^ T lymphocytes from HIV-negative donors were activated, infected with HIV_NL4-3_, and treated with fentanyl. HIV proviral DNA and p24 antigen expression were quantified using real-time PCR and ELISA, respectively. Single-cell RNA libraries were analyzed to identify differentially expressed genes.

**Results:**

Results indicated that fentanyl treatment increased HIV p24 expression and proviral DNA levels, and naltrexone mitigated these effects. Single-cell RNAseq analysis identified significantly altered gene expression in CD4^+^ T lymphocytes.

**Discussion:**

The results of our findings suggest that fentanyl promotes HIV replication *ex vivo*, emphasizing the need for a deeper understanding of opioid-virus interactions to develop better treatment strategies for individuals with HIV and opioid use disorder.

## 1 Introduction

The prevalence of substance use disorder (SUD) in the United States exceeds 20 million individuals (Abuse, 2020). Overdose deaths, mainly attributed to fentanyl use, have driven the opioid crisis (Althoff et al., 2020). The number of deaths from opioid overdoses in the United States increased each year from 2019 to 2022 (Moghtaderi et al., 2023). Over 70,000 of those deaths were caused by illicitly manufactured fentanyl (Scholl, 2019). The opioid crisis in the United States is also associated with an elevated risk of infection (Blackard et al., 2019).

Opioids, including endogenous, exogenous, and synthetic substances, activate opioid receptors on various immune cells, impacting both innate and adaptive immune functions (Tahamtan et al., 2016). The concern over fentanyl’s impact on HIV pathogenesis stems from its ability to modulate immune function and increase susceptibility to infection (Blackard and Sherman, 2021). Opioids can influence various aspects of immune function, enhance viral replication, and contribute to virus-induced pathology (Wickham and Wickham, 2016). For instance, morphine stimulated the replication of HIV in human Kupffer cells (Schweitzer et al., 1991). Morphine also enhanced CXCR4 and CCR5 expression and subsequent virion binding, trafficking of HIV-infected cells, and enhanced disease progression (Steele et al., 2003). Furthermore, HIV replication was elevated in morphine-exposed immune cells (Li et al., 2003; Peterson et al., 1990). Xu Wang et al. demonstrated that morphine and heroin suppress the expression of anti-HIV miRNAs in monocytes, leading to increased susceptibility to HIV infection *in vitro* and *in vivo* (Wang et al., 2011). This was further supported by Reynolds et al. who demonstrated that heroin potentiates HIV replication in astrocytes (Reynolds et al., 2006). Wang et al. also observed that heroin inhibits the expression of HIV restriction miRNAs in macrophages, further enhancing HIV replication (Wang et al., 2015). Liang et al. showed that morphine increased the generation of HIV drug resistance mutations (Liang et al., 2017). Collectively, these studies suggest that drugs of abuse use can exacerbate HIV replication and potentially contribute to the development of drug-resistant mutations.

We previously reported how synthetic opioid fentanyl impacts HIV replication and chemokine co-receptor expression, potentially increasing the risk of transmission and disease progression. Bulk RNA sequencing analysis revealed differential regulation of genes associated with antiviral response, cell death, and immune signaling in fentanyl-treated cells (Madhuravasal Krishnan et al., 2023). Fentanyl has a proviral effect on HBV and HCV, induces apoptosis, and alters NFκB signaling (Kong et al., 2021).

These findings underscore the ability of fentanyl to promote viral replication *in vitro*. However, its effects on viral replication *in vivo* remain poorly characterized, prompting investigations into its impact on cellular gene expression using single-cell transcriptomics.

## 2 Methods

### 2.1 Cell lines and reagents

#### 2.1.1 Compounds

Fentanyl and naltrexone were obtained as Certified Reference Materials from Cerilliant (Round Rock, TX). Per their certificates of analysis, these reagents are suitable for the identification, calibration, and quantification of analytes in analytical and R&D applications (Armenian et al., 2018). Both drugs were diluted with dH_2_O to concentrations of 10 μg/mL.

#### 2.1.2 Primary cell isolation and culture

**Figure 1:**
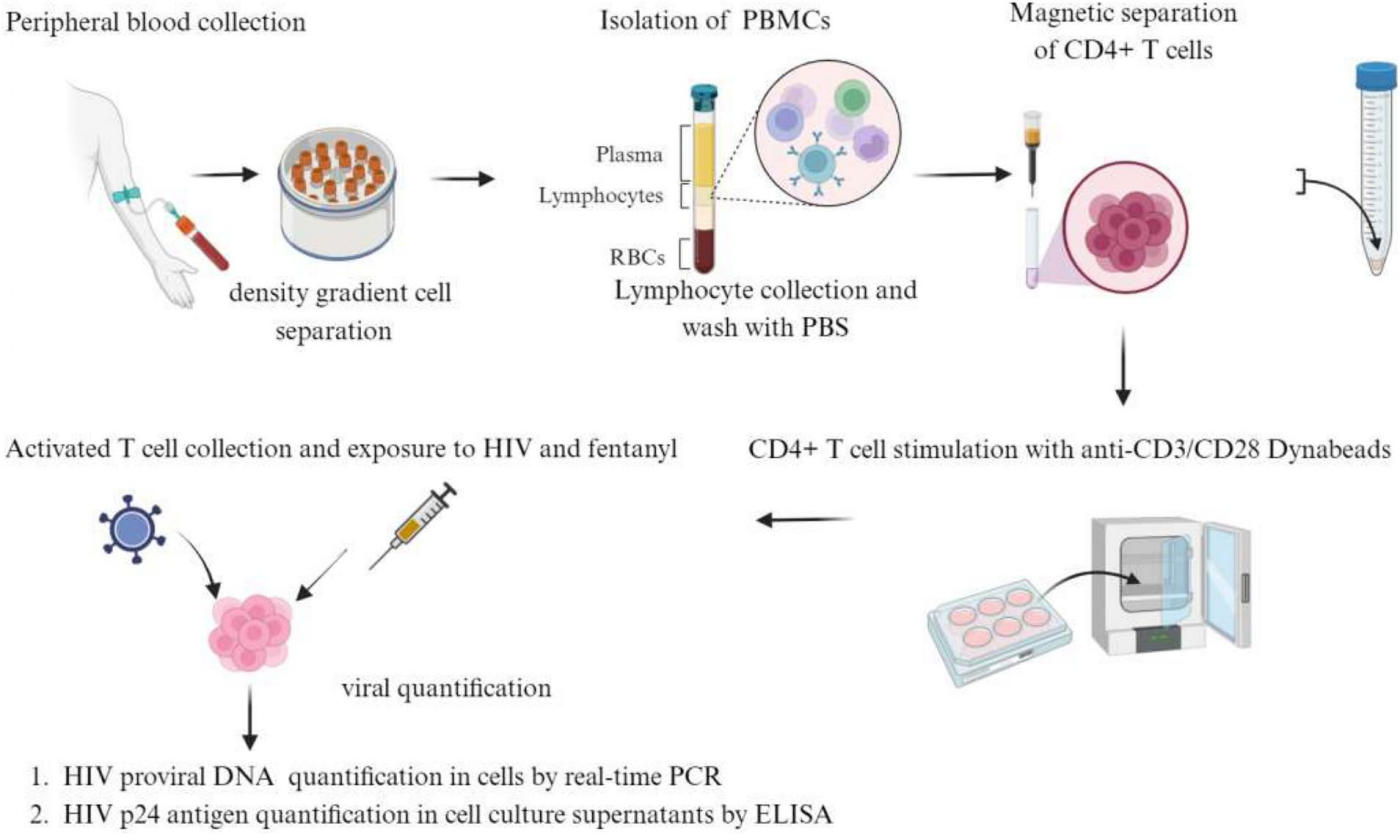
Primary cell isolation, activation, and exposure to HIV and fentanyl.

Peripheral blood samples were obtained from healthy adult donors who were HIV-1 antibody negative and had no history of drug abuse. Four mL of blood was collected in 10 BD Vacutainer Cell Preparation Tubes (catalog #362760). Tubes were inverted 8–10 times and centrifuged for 25 min at 1900 RCF. Mononuclear cells were collected in 15 mL centrifuge tubes and rinsed two times with phosphate-buffered saline (PBS). The cell pellet was resuspended in 5 mL of PBS, and the total number of cells in the suspension was determined by trypan blue staining. Naïve CD4^+^ T lymphocytes were isolated by negative selection using the naïve CD4^+^ T cell isolation kit (cat. no. 130–096-533, Miltenyi Biotec GmbH, Bergisch Gladbach, Germany) according to the manufacturer’s instructions. Briefly, the PBMC cell suspension was centrifuged at 800 g for 5 min. The cell pellet was resuspended in 40 μL of MACS Running Buffer (PBS + 5% FBS) per 1 × 10^7^ total cells. Ten μL of CD4^+^ T cell Biotin-antibody Cocktail was added per 1 × 10^7^ total cells and incubated at 4°C for 10 min. Thirty μL of MACS Running Buffer and 20 μL of Anti-Biotin Microbeads per 1 × 10^7^ total cells were added and incubated at 4°C for 10 min. Cells were resuspended in MACS running buffer and applied to a LS column placed in a magnet. Unlabeled CD4^+^ T cells were collected and centrifuged at 800 g for 5 min. Cells were resuspended with 1 mL of MACS running buffer, and the total cell count was determined by trypan blue staining. Cells were centrifuged again and resuspended in 1 mL RPMI +10% FBS + 1% antibiotics (Pen/Strep) + 1% glutamine. Purified cells were seeded at 0.3 × 10^6^ cell per well in a 96-well plate in 200 μL of RPMI +10% FBS + 1% antibiotics (Pen/Strep) + 1% glutamine. The cells were stimulated with anti-CD3/CD28 dynabeads (cat. no. 111.31D, Invitrogen, 1 cell per 3 beads) + 100 IU/mL IL-2 at 37°C in 5% CO_2_ for 3 days. After 3 days of incubation, the activated cells were collected, transferred to a 5 mL polystyrene tube, and placed in a dynabead magnet to remove the beads from the mixture. The cells were then transferred to a new tube and centrifuged at 800 g for 5 min. The cell pellet was resuspended in 1 mL of RPMI (Life Technologies) supplemented with 10% heat-inactivated FBS, 100 U/mL penicillin, and 100 μg/mL antibiotics (Life Technologies), and the cell count was determined before proceeding with infection or drug exposure. Figure 1 illustrates a detailed overview of the process for isolating primary cells from PBMCs and exposing them to HIV and drugs.

### 2.2 Propagation of HIV

Infectious HIV was prepared by transfection of 1 × 10^6^ 293 T cells (ATCC #CRL-3216) per well with 2 μg of the full-length HIV_NL4-3_ plasmid obtained from the NIH AIDS Reagent Program by the FuGene6 transfection reagent (Roche; Basel, Switzerland). Transfected cells were incubated at 37°C for 48 h. The supernatant was harvested and passed through a 0.20 μm filter to remove cellular debris and then precipitated in polyethylene glycol at 4°C. The precipitated virus was centrifuged at 3000 g for 20 min, and the virus precipitate was resuspended in phosphate-buffered saline (PBS) and preserved at −80°C. The virus was tittered using TZM-bl cells and *β*-galactosidase staining. HIV p24 protein in cell culture supernatants was also quantified as outlined below.

### 2.3 HIV infection, drug exposure, and p24 protein quantification

~1 × 10^5^ CD4^+^ T lymphocytes were seeded per well. Cells were infected with HIV_NL4-3_ at MOI of 1 for 2 h and rinsed 3 times with RPMI +10% FBS + 1% antibiotics (Pen/Strep) + 1% glutamine to remove any unbound virus and replaced with fresh media. Fentanyl/naltrexone at a concentration of 10 μg/mL was added to the respective well and incubated at 37°C in 5% CO_2_. After 72 h of drug incubation, the expression of HIV p24 protein was quantified in cell culture supernatants using the HIV p24 ELISA Kit (Abcam; Cambridge, MA) with a lower limit of sensitivity of 1.1 pg./mL.

### 2.4 Quantification of integrated HIV DNA

CD4^+^ T lymphocytes were seeded per well. Cells were infected with HIV_NL4-3_ at MOI of 1 for 2 h and rinsed 3 times with RPMI +10% FBS + 1% antibiotics (Pen/Strep) + 1% glutamine to remove any unbound virus and replaced with fresh media. Fentanyl/naltrexone at a concentration of 10 μg/mL was added to the respective well and incubated at 37°C in 5% CO_2_. After 72 h of drug incubation, cellular DNA was extracted from cells using the Qiagen mini kit per the manufacturer’s instruction. DNA was similarly extracted from ACH-2 cells that contain a single copy of HIV-1 proviral DNA (LAV strain) (Mann et al., 1989) and used as a positive control to quantify virus levels. The number of HIV-1 proviral copies was quantified by real-time PCR amplification using Brilliant III ultra-fast SYBR green QPCR master mix (Agilent) as described elsewhere (Gibellini et al., 2004). Real-time PCR was performed using SYBR green 2x Master Mix with 200 nM of each oligonucleotide primer targeting the HIV-1 pol gene and DNA extracted from cells treated and untreated with HIV_NL4-3_ +/− fentanyl +/− naltrexone. To quantify HIV-1 provirus, a standard curve was defined using serial dilutions of ACH-2-derived DNA ranging from 1 to 10^6^ copies per cell. All standard dilutions, controls, and samples were run in duplicate, and the average value ct was utilized to quantify HIV-1 DNA copies.

### 2.5 Sample processing for 10x single-cell RNA sequencing

To prepare CD4^+^ T lymphocytes infected with HIV in the presence or absence of fentanyl for 10x single-cell labeling, cells were counted, and viability was confirmed as greater than 90%, and the concentration was adjusted to 1 × 10^6^ cells/mL in Dulbecco’s phosphate-buffered saline.

Single-cell RNA sequencing (scRNAseq) analysis was conducted following a rigorous protocol. Cell barcoding and complementary DNA (cDNA) synthesis were carried out at the Cincinnati Children’s Hospital Medical Center single cell genomics facility (RRID: SCR_022653) using Chromium Next GEM Single Cell 5’ Reagent Kits v2 (Dual index) following the manufacturer’s instructions. The process involved loading cell suspensions, beads, master mix, and partitioning oil onto a “K” chip to target an output of 10,000 cells per library, which was then run on the Chromium X platform. Reverse transcription was performed at 53°C for 45 min, followed by cDNA amplification for 14 cycles using a Bio-Rad C1000 Touch thermocycler. Subsequently, cDNA size selection was conducted using SpriSelect beads, and the quality of the obtained cDNA was verified using an Agilent Bioanalyzer High Sensitivity chip. DNA fragmentation, end-repair, A-tailing, and ligation of sequencing adapters were performed according to the manufacturer’s protocol (10x Genomics, USA). The prepared libraries were sequenced on a NovaSeq 6,000 S1 or S4 flow cell at the DNA Sequencing Core Facility of Cincinnati Children’s Hospital Medical Center. The raw base call files were de-multiplexed using Cell Ranger v6.0.0 mkfastq. The reads were aligned to the human reference genome GRCh38, and gene expression was quantified using Cell Ranger count. Further analysis of the data was conducted using Seurat v4.0.5 in R v4.1.2. Cells displaying more than 20% mitochondrial gene expression or < 100 total expressed genes were excluded from analysis. Gene expression counts were normalized using the NormalizeData function in Seurat, which employs a logarithmic normalization method. Samples were integrated using the FindIntegrationAnchors and IntegrateData functions from Seurat and used for principal component analysis, variable gene identification, Shared Nearest Neighbor (SNN) clustering analysis, and Uniform Manifold Approximation and Projection (UMAP).

Cell types and clusters were annotated using a high-quality peripheral blood mononuclear cell (PBMC) dataset (Hao et al., 2021). Cell annotations were transferred from a multi-model PBMC reference dataset using the FindTransferAnchors and MapQuery functions in Seurat.

Differentially expressed genes (DEGs) were identified using the Wilcoxon rank sum test, considering a log_2_ fold change (log_2_FC) ≥ 0.5, a minimum of 10% of cells expressing the gene in both compared groups, and a Bonferroni-adjusted *p*-value <0.05. All plots were generated using R ggplot2 v3.3.5 (Kassambara, 2018), ggpubr v0.4.0 (Kaimal et al., 2010), and Seurat. Enrichment analysis was performed using ToppCluster and Enricher-KG (Chen et al., 2013; Kuleshov et al., 2016; Xie et al., 2021) to enable the identification of specialized biological functions and regulatory networks.

### 2.6 Statistical analysis

The standard deviation of each experimental condition was represented by error bars for technical duplicates. An ANOVA with replication was used to evaluate the statistical significance (*p* < 0.05) for fentanyl compared to the no drug condition.

## 3 Results

We previously showed that fentanyl increases replication of HIV in several cell types (Madhuravasal Krishnan et al., 2023; Kong et al., 2021). Thus, we evaluated the potential impact of fentanyl on HIV expression. As shown in Supplementary Figures S1–S5, fentanyl led to an increase in HIV p24 protein expression at 72 h post-drug exposure in each of the five PBMC donors. Log_10_ copies of HIV proviral DNA were also increased in the presence of fentanyl compared to HIV-infected, drug-naïve CD4^+^ T lymphocytes.

**Figure 2:**
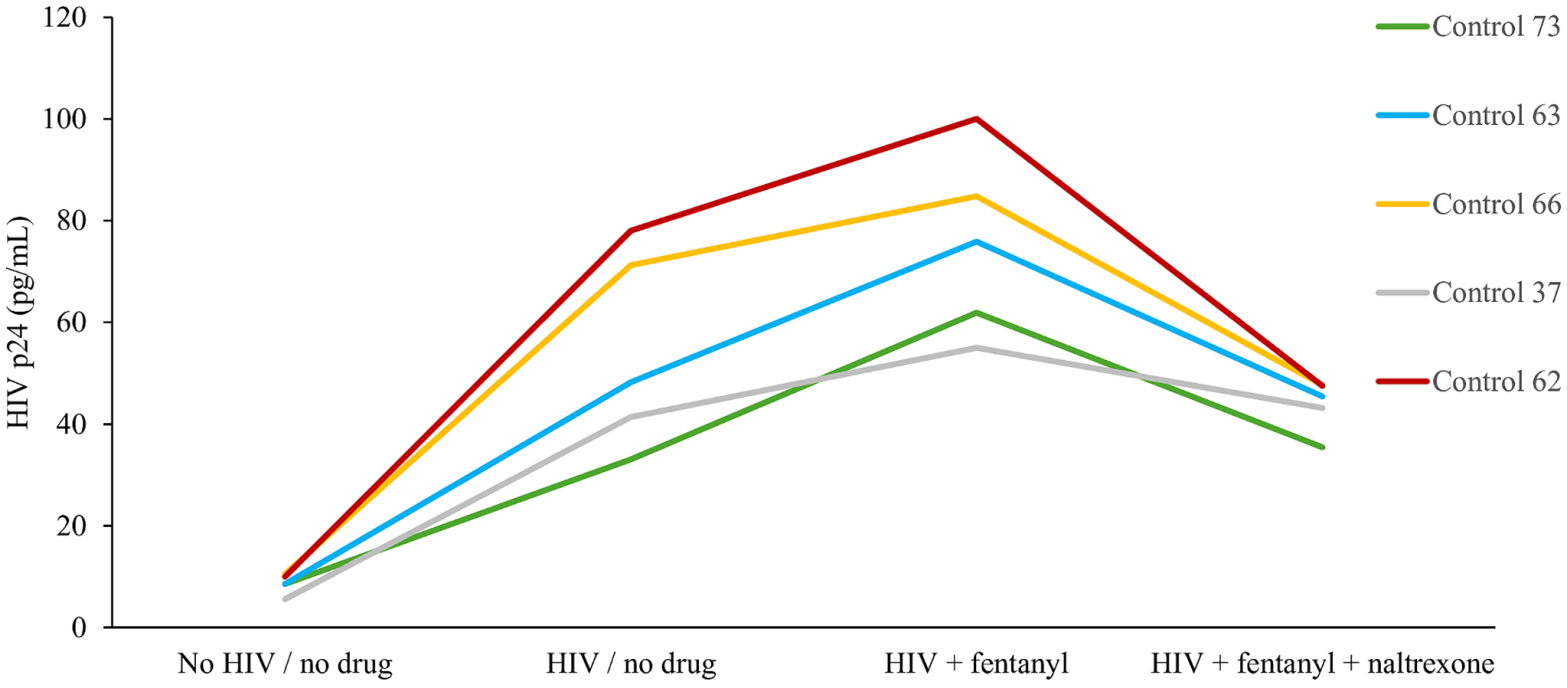
PBMC-derived T cells were seeded at ~1 × 10^5^ cells per well. Ten ug of naltrexone was added, and cells were incubated for 24 h. After incubation, cells were infected with HIV_NL4-3_ at MOI of 1 for 2 h and rinsed with RPMI +10% FBS + 1% antibiotics (Pen/Strep) + 1% glutamine three times to remove any unbound virus and replaced with fresh media. Fentanyl at a concentration of 10 ug/mL was added to the respective well and incubated. After incubation with drug and virus for 72 h, HIV p24 antigen expression was estimated from the cell culture supernatant. Anova for fentanyl versus no drug = 0.0030.

**Figure 3:**
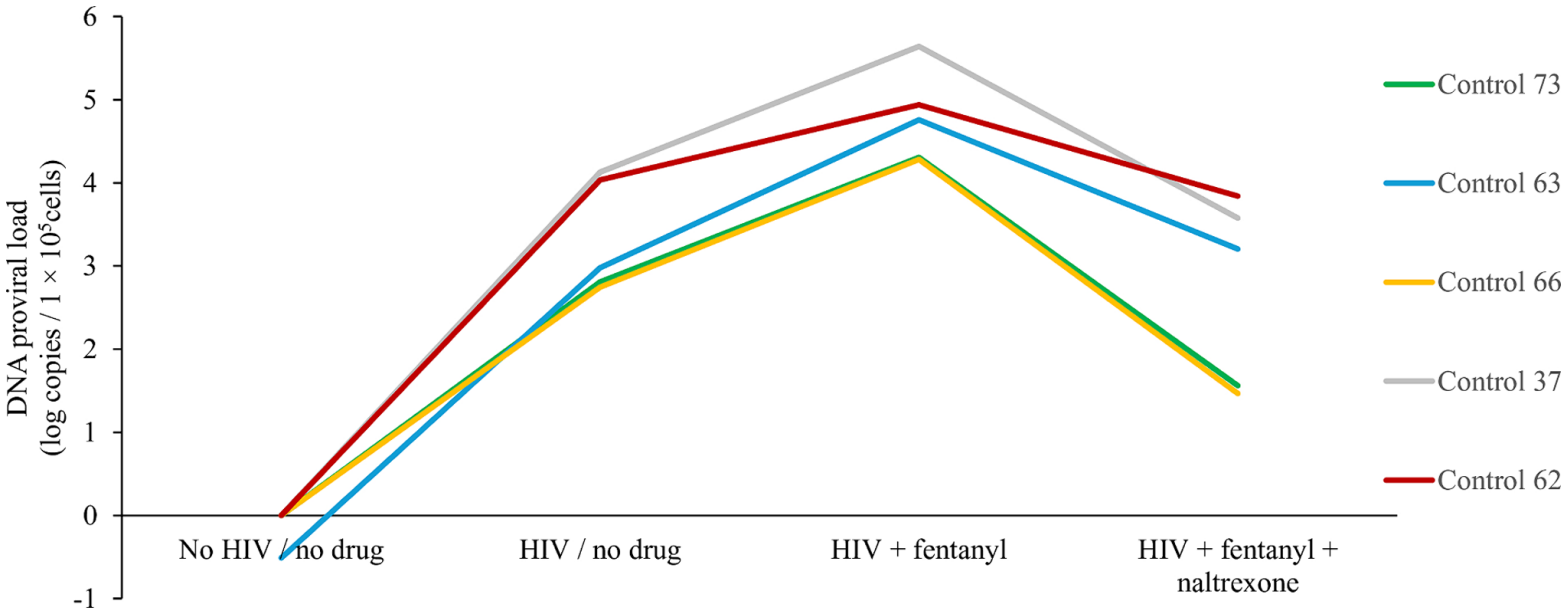
PBMC-derived T cells were seeded at ~1 × 10^5^ cells per well. Ten ug of naltrexone was added, and cells are incubated for 24 h. After incubation, cells were infected with HIV_NL4-3_ at MOI of 1 for 2 h and rinsed with RPMI +10% FBS + 1% antibiotics (Pen/Strep) + 1% glutamine three times to remove any unbound virus and replaced with fresh media. Fentanyl at a concentration of 10 ug/mL was added to the respective well and incubated. After incubation with drug and virus for 72 h, HIV proviral DNA was quantified in cells by real-time PCR based on SYBR Green I detection from the cells of each five distinct controls. Anova for fentanyl versus no drug = 0.0063.

Across the PBMC donors, fentanyl exposure resulted in a median enhancement of HIV p24 expression of 75.51 pg./mL compared to 54.37 pg./mL for the no drug HIV-infected cells (Figure 2). The effect of fentanyl on HIV proviral DNA levels was similar but more dramatic. Median HIV proviral DNA levels were 4.78 log_10_ copies / 1 × 10^5^ cells in fentanyl-treated PBMCs compared to a median of 3.33 log_10_ copies / 1 × 10^5^ in HIV-infected, fentanyl-naïve cells (Figure 3). HIV DNA was not detected in the no drug / no HIV negative controls.

Naltrexone is an opioid antagonist used to treat opioid and alcohol use disorder and can limit HIV replication induced by opioids. Treatment with 10 μg/mL naltrexone blocked the effect of fentanyl on HIV replication. The addition of naltrexone significantly reduced the proviral effect of fentanyl by ~2 log_10_ and diminished antigen expression to 43.83 pg./mL.

**Figure 4:**
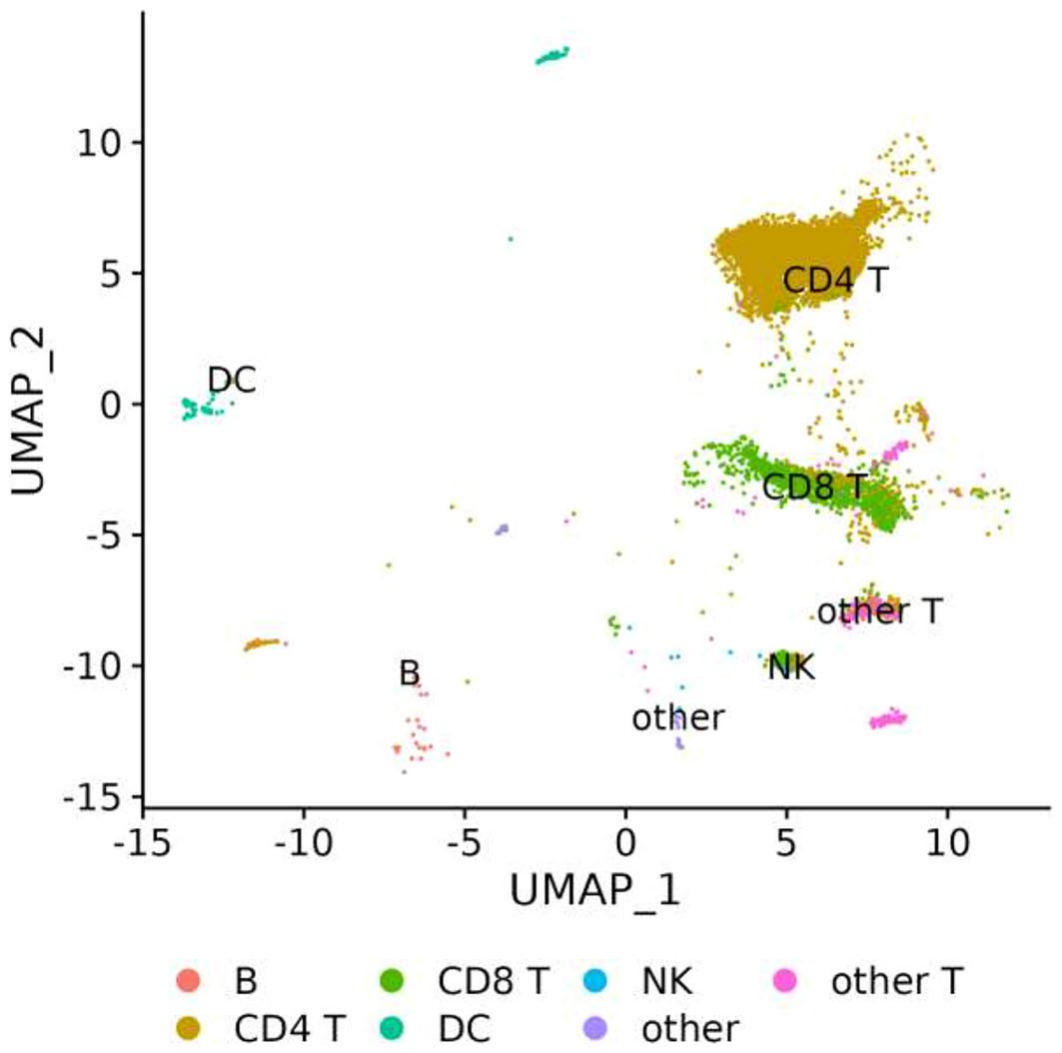
UMAP visualization of PBMCs infected with HIV_NL4-3_ in the presence/absence of fentanyl. Each circle represents an individual cell colored by cell type.

**Figure 5:**
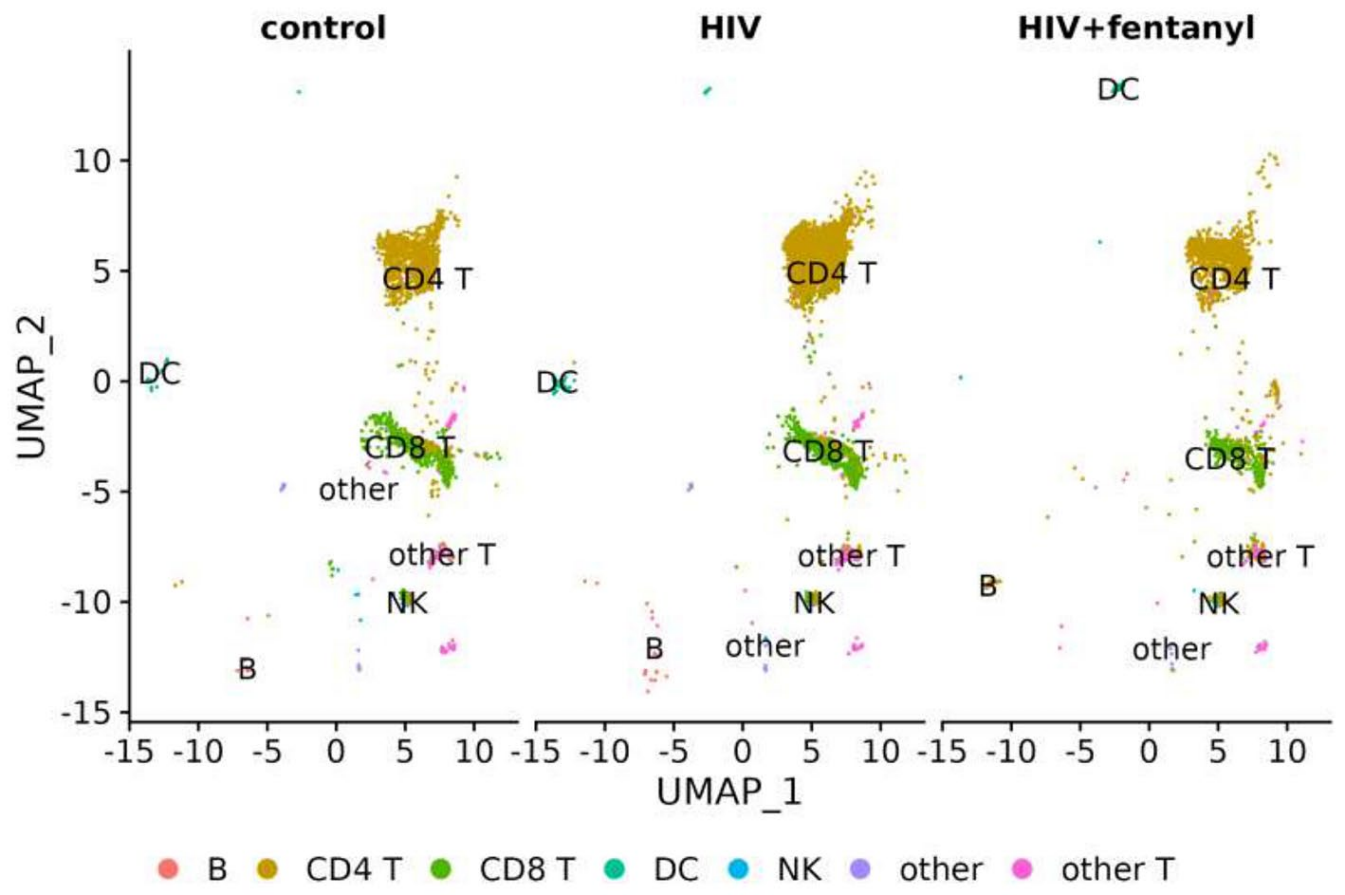
UMAP visualization of PBMCs for each condition, PBMC control, and PBMC infected with HIV in the presence or absence of fentanyl. Each circle represents an individual cell colored by cell type.

The genes involved in fentanyl-induced HIV replication are largely unknown. Thus, we conducted single-cell RNAseq in CD4^+^ T lymphocytes that were infected with HIV_NL4-3_ in the presence/absence of fentanyl. After quality control, a total of 24,914 cells were included in the single-cell RNAseq analysis. The median number of cells passing the mitochondrial filter was 24,886. A high-quality reference dataset (Wang et al., 2011) was used to identify cell clusters within PBMCs from all three groups (Figure 4). An overview of the data visualizations for each group is provided in Figure 5. The total number of the CD4^+^ T under each condition compared to other cell types is listed in Table 1. The single-cell RNAseq data are available under accession PRJNA1116308.

**Table 1:** The number of CD4^+^ T lymphocytes versus other cell types under each condition.

Group	Predicted cell type	Cell count	Cell percent
Control	CD4^+^ T cells	6,589	88%
Control	Other cell types	931	12%
HIV	CD4^+^ T cells	9,412	89%
HIV	Other cell types	1,210	11%
HIV + fentanyl	CD4^+^ T cells	3,087	84%
HIV + fentanyl	Other cell types	597	16%

**Figure 6:**
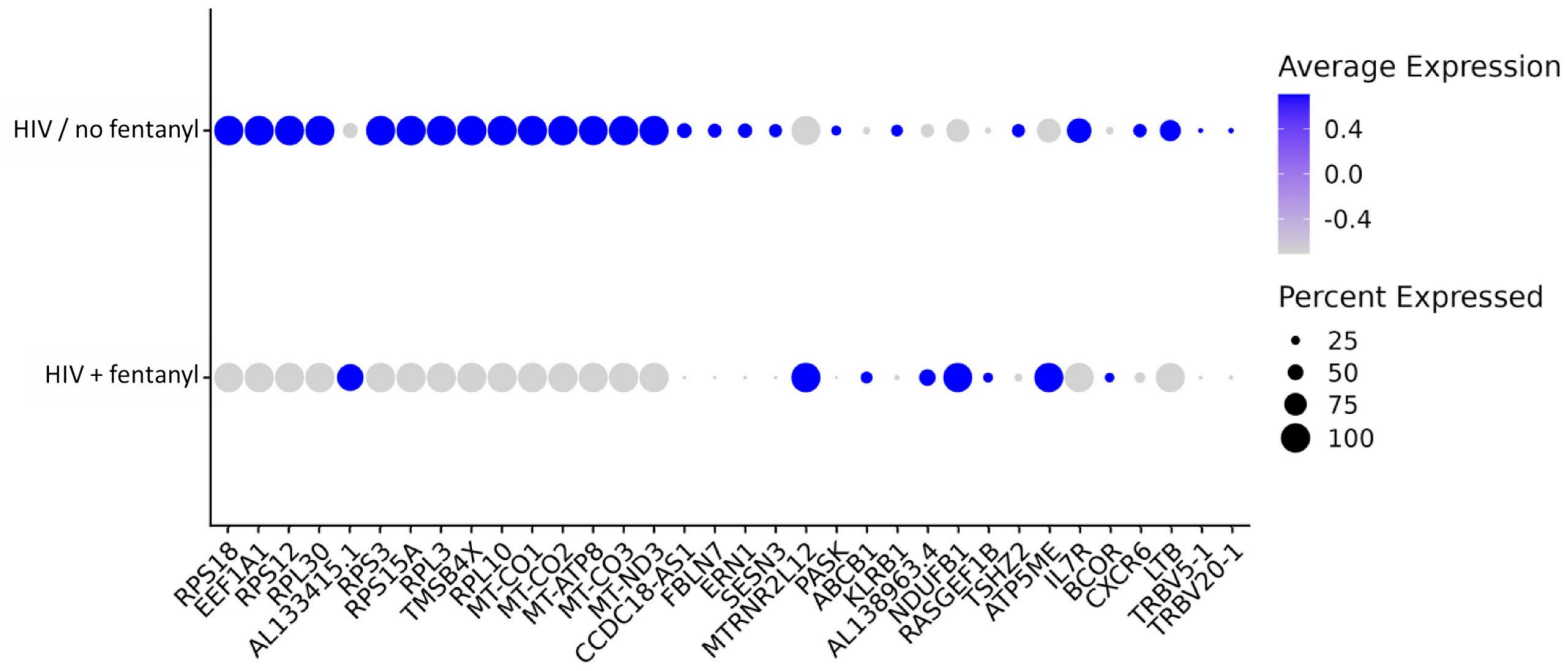
Dot plot of differentially expressed genes in CD4^+^ T lymphocytes infected with HIV_NL4-3_ with and without fentanyl exposure.

DEGs that were differentially expressed by CD4^+^ T lymphocytes are listed in Supplementary Table S1. For HIV-infected CD4^+^ T lymphocytes in the presence/absence of fentanyl, there were 34 differentially expressed genes (DEGs) with an adjusted *p* value less than 0.05 and an absolute log_2_ fold change greater than 0.5 (Figure 6 and Table 2), including 26 DEGs that were lower and 8 DEGs that were higher in fentanyl-exposed, HIV-positive cells.

**Table 2:** List of differentially expressed genes (DEGs) with an adjusted *p*-value less than 0.05 and an absolute log_2_ fold change greater than 0.5 in CD4^+^ T lymphocytes.

DEGs	*p*-value	Average log_2_FC	Percent in group 1	Percent in group 2	Adjust *p*-value
MT-CO2	0	−1.01215	1	0.999	0
TRBV20-1	1.39E-10	−0.99662	0.095	0.131	3.05E-06
TRBV5-1	3.88E-12	−0.95095	0.069	0.107	8.55E-08
KLRB1	7.9337E-135	−0.85565	0.12	0.357	1.7469E-130
MT-ATP8	0	−0.79407	0.998	0.99	0
IL7R	3.12E-49	−0.7542	0.997	0.824	6.86E-45
MT-ND3	0	−0.66864	1	0.997	0
EEF1A1	0	−0.63889	1	1	0
RPS3	0	−0.61477	1	1	0
LTB	2.44E-15	−0.58946	0.99	0.695	5.38E-11
CXCR6	6.50E-45	−0.58618	0.319	0.414	1.43E-40
RPL10	0	−0.56763	1	1	0
RPS18	0	−0.56645	1	1	0
PASK	5.57E-185	−0.56499	0.029	0.287	1.23E-180
SESN3	1.71E-242	−0.54846	0.061	0.405	3.77E-238
RPL3	0	−0.54552	1	1	0
MT-CO3	0	−0.54195	1	0.997	0
TMSB4X	0	−0.52561	1	1	0
FBLN7	2.44E-280	−0.52293	0.054	0.431	5.37E-276
RPL30	0	−0.52173	1	1	0
TSHZ2	4.60E-93	−0.5163	0.217	0.405	1.01E-88
RPS12	0	−0.5152	1	1	0
MT-CO1	0	−0.51371	1	1	0
ERN1	1.97E-279	−0.50579	0.062	0.446	4.34E-275
RPS15A	0	−0.50399	1	1	0
CCDC18-AS1	2.51E-304	−0.50387	0.06	0.464	5.53E-300
ATP5ME	3.14E-92	0.509268	0.992	0.802	6.92E-88
NDUFB1	8.84E-109	0.535544	0.992	0.769	1.95E-104
MTRNR2L12	7.3568E-186	0.546561	1	0.988	1.6199E-181
BCOR	9.53E-47	0.643097	0.273	0.2	2.10E-42
RASGEF1B	5.4075E-103	0.733849	0.284	0.151	1.19067E-98
AL133415.1	0	0.896048	0.9	0.477	0
AL138963.4	5.81E-117	0.917569	0.529	0.415	1.28E-112
ABCB1	6.30E-147	0.922118	0.36	0.197	1.39E-142

**Figure 7:**
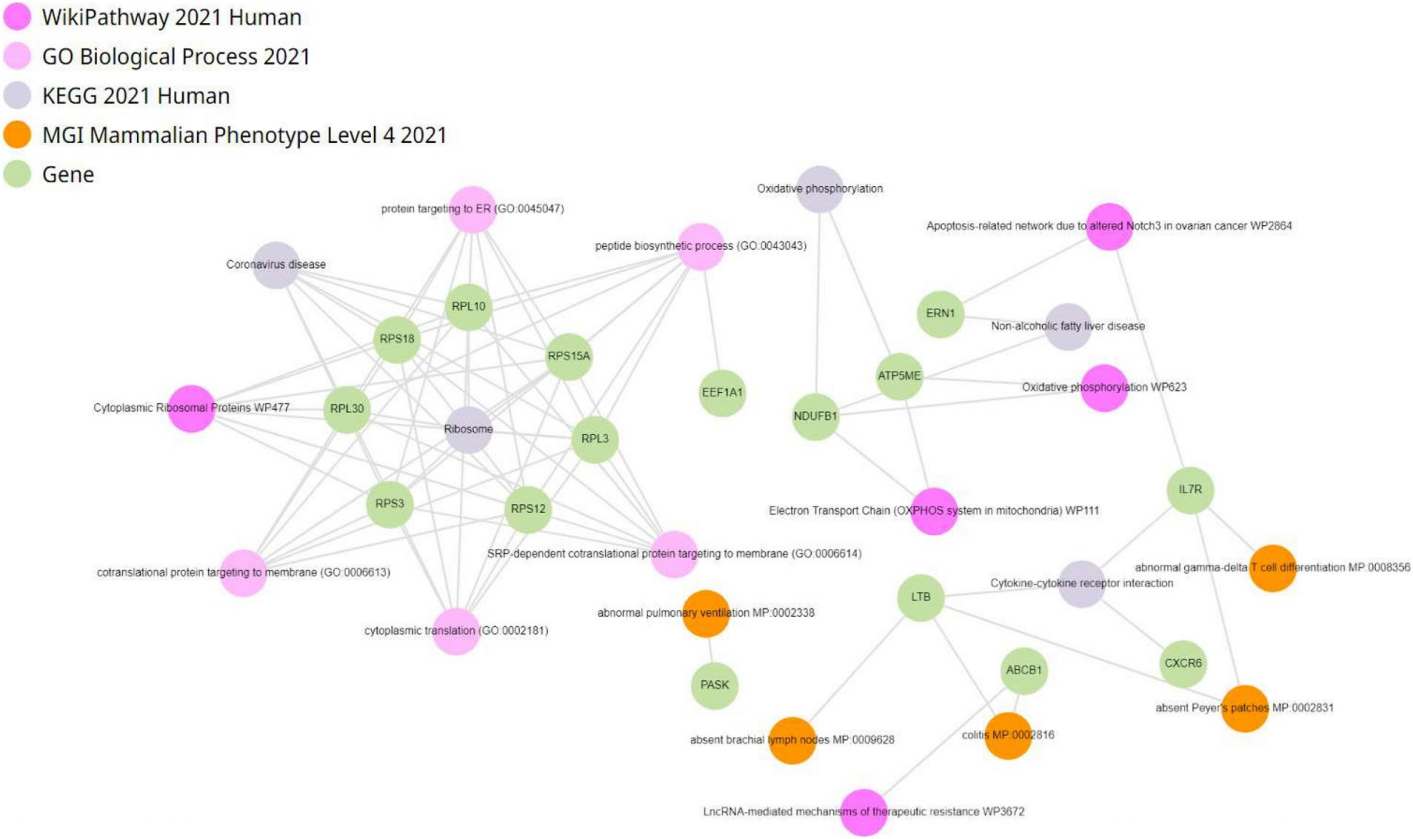
Enrichment analysis of differentially expressed genes in CD4^+^ T lymphocytes.

Enrichment analysis of differentially expressed genes was conducted within CD4^+^ T lymphocytes. Genes were enriched in oxidative phosphorylation, electron transport in ATP synthesis, mitochondria, and electron transport chain (Figure 7). To investigate the impact of fentanyl on immune signaling pathways, we conducted dot plot analysis to identify expression patterns of genes that are differentially expressed in fentanyl-exposed cells. Our findings reveal a downregulation of several genes associated with interferon signaling and restriction factors (see Supplementary Figures S7, S8).

## 4 Discussion

Single-cell RNA sequencing has been used to investigate the impact of opioids on HIV replication. Steele et al. found that opioids, particularly *μ*-opioids, can increase the expression of HIV-1 coreceptors CXCR4 and CCR5, leading to enhanced viral replication (Steele et al., 2003). This was further supported by Wang et al. who demonstrated that opioids inhibited the expression of anti-HIV microRNAs, thereby increasing the susceptibility of monocytes to HIV infection (Wang et al., 2015). Single-cell RNA sequencing studies have revealed that opioid usage, including heroine and morphine, can suppress the antiviral gene program in various immune cell types, potentially increasing susceptibility to viral infections (Karagiannis et al., 2020). Furthermore, fentanyl has been shown to enhance viral replication *in vitro*, suggesting a potential link between opioid use and viral pathogenesis. Single-cell RNA sequencing revealed cell-type-specific responses to morphine, with a focus on the significant transcriptional changes in oligodendrocytes (Avey et al., 2018). However, Basukala et al. found that while opioid use May not directly impact HIV replication and latency in CD4^+^ T lymphocytes, it can shape the HIV reservoir *in vivo* by modulating general immune functions (Basukala et al., 2023). These studies collectively suggest that single-cell RNA sequencing can provide valuable insights into the complex interplay between opioids and HIV replication. Therefore, we performed single-cell RNA sequencing on the CD4^+^ T lymphocytes isolated from healthy PBMC donors. Several genes of interest were found to be differentially expressed in fentanyl-treated CD4^+^ T lymphocytes. Fentanyl-induced changes have significant implications for the expression and regulation of several genes involved in HIV pathogenesis and immune response.

AL133415.1 is a long non-coding RNA (lncRNA) that plays a vital role in Alzheimer’s disease pathology by regulating neuronal apoptosis, cell viability, and oxidative stress (Foret et al., 2020). Fentanyl-induced modulation of AL133415.1 could impact similar pathways in the context of HIV, affecting neuronal health and immune response. AL138963.4 is another lncRNA expressed in CD4^+^ T cell clones with intact provirus. Its role in viral pathogenesis is not fully understood but it is involved in cytokine production and immune cell activity (Paquette et al., 1992). Changes in AL133425.1 and AL138963.4 expression due to fentanyl could alter the immune response to HIV and affect viral replication.

KLRB1 – also known as CD161 – is a C-type lectin receptor found on natural killer cells (NK cells), T cells, and subsets of innate lymphoid cells. Due to its involvement in immune regulation and response, it plays an important role in HIV infection and pathogenesis (Riccardi et al., 1990). KLRB1 is expressed on NK cells, where it regulates their activity. Early during infection, HIV replication is limited by NK cells which directly kill infected cells and produce antiviral cytokines (Lotzová, 2019). KLRB1 May influence NK cell cytotoxicity and cytokine production, potentially affecting their ability to control HIV infection. In addition to CD8^+^ T cells and mucosal-associated invariant T cells (MAIT), KLRB1 is also expressed on subsets of T cells. These T cell populations are involved in immune surveillance and response to HIV (Wong et al., 2013). The expression of KLRB1 on T cells May influence their activation, proliferation, and the production of cytokines during HIV infection. While KLRB1 itself is not a receptor for HIV entry, its expression on immune cells May indirectly affect viral replication. HIV-infected cells May interact with KLRB1-expressing cells or modulate the immune microenvironment, influencing viral replication (Khaitan et al., 2016). Dysregulated immune activation and inflammation are hallmark features of HIV infection (Cua and Tato, 2010). Our study results show that fentanyl induces KLRB1 downregulation, thereby impacting NK cell cytotoxicity and cytokine production, which affects the control of HIV infection and immune activation.

ABCB1 – also known as P-glycoprotein – is a transporter protein involved in drug efflux and cellular detoxification. Its role in HIV pathogenesis includes contributions to drug resistance, regulation of drug penetration into the central nervous system, modulation of immune responses, and implications in HIV-associated cancers (Lucia et al., 2011). Overexpression of ABCB1 is a major challenge in HIV treatment as it contributes to multidrug resistance. Cells with high ABCB1 activity can expel multiple antiretroviral agents, reducing their efficacy and necessitating higher doses or alternative therapies (Pagnotta et al., 2021). Although the precise role of ABCB1 in HIV is not fully understood, it remains a significant factor in HIV research and treatment due to its impact on drug efficacy and disease progression. Our study results indicate that fentanyl-induced upregulation of ABCB1 can significantly hinder the effective treatment of HIV, particularly in populations with difficult-to-treat infections or those with a history of drug abuse.

Ribosomal proteins such as RPS18, RPS12, RPS3, and RPS15A play significant roles in facilitating the replication and translation of viral proteins (Zhao et al., 2011). RPS18 aids in the translation of HIV mRNA into viral proteins. It helps ensure that the viral RNA is efficiently translated by the host ribosomal machinery, promoting the production of essential HIV proteins required for viral replication and assembly (Lucas, 2012). The ribosomal protein RPS3 plays a significant role in the pathogenesis of HIV. It is involved in various stages of the viral life cycle, including viral replication and immune response modulation. Studies have shown that RPS3 can interact with HIV proteins, potentially influencing viral replication efficiency and the host cell response to infection. RPS3’s involvement in translation directly impacts the synthesis of HIV proteins. Additionally, its role in DNA repair May be exploited by HIV to maintain the integrity of its genetic material or to manipulate host cellular responses to favor viral replication (Kim and Kim, 2018). RPS15A contributes to the efficient initiation of translation of HIV mRNA. This ensures that viral proteins are produced in sufficient quantities for successful viral replication and infection of new cells (Ramos et al., 2022). Fentanyl May alter their expression, affecting the efficiency of HIV protein synthesis and viral replication.

CXCR6 is a chemokine receptor that can serve as an alternative coreceptor for HIV entry into specific immune cells (Islam et al., 2013). It plays a crucial role in immune cell trafficking and localization, influencing the distribution and persistence of HIV-infected cells in tissues. CXCR6-expressing cells contribute to chronic immune activation and inflammation, exacerbating HIV-associated immune dysfunction. These cells May also act as viral reservoirs, harboring latent HIV and complicating eradication efforts. Variations in CXCR6 expression can impact disease progression, making it a promising target for therapeutic strategies against HIV (Gosselin et al., 2017). Downregulation of CXCR6 by fentanyl could reduce chronic immune activation and inflammation, potentially impacting the distribution and persistence of HIV-infected cells and influencing disease progression. IL7R plays a critical role in T-cell survival and function. In HIV, dysregulated IL7R signaling can worsen CD4^+^ T-cell depletion, chronic immune activation, and T-cell exhaustion. Additionally, IL7R’s involvement in viral reservoir maintenance complicates HIV treatments (Chahroudi and Silvestri, 2010). Understanding IL7R’s role in HIV pathogenesis is vital for developing effective treatments. Downregulation of IL7R by fentanyl as noted in our study suggests that this could impair T-cell survival and function, potentially exacerbating CD4^+^ T-cell depletion and chronic immune activation, complicating efforts to control and cure HIV.

MT-CO2 encodes a subunit of the cytochrome c oxidase enzyme complex involved in the electron transport chain in mitochondria (Poulos et al., 1982; Moon, 1994). This enzyme plays a crucial role in cellular respiration, facilitating the final step in the electron transport chain by transferring electrons to oxygen molecules, leading to the production of water and ATP (Dornas and Lagente, 2019). Mitochondrial dysfunction has been implicated in various aspects of HIV infection, including metabolic abnormalities, neurocognitive disorders, and cardiovascular diseases (Edwards et al., 2019; Pantaleo et al., 1993). Our study results indicate that fentanyl-induced downregulation of MT-CO2 May exacerbate mitochondrial dysfunction, thereby impairing cellular respiration and contributing to complications associated with HIV.

The downregulation of MT-ATP8 observed in our study May have significant implications. Given that MT-ATP8 encodes a subunit of the ATP synthase complex, its downregulation could impair mitochondrial function and ATP production, which are critical for cellular energy metabolism (Amodeo et al., 2020). This impairment May contribute to mitochondrial dysfunction, exacerbating the inflammatory response and further dysregulating immune signaling pathways (Gupta et al., 2015; Alhetheel et al., 2013). Additionally, reduced expression of MT-ATP8 could influence the cellular response to HIV, potentially leading to increased susceptibility to HIV-induced cellular dysfunction and apoptosis. The downregulation May also affect the overall immune response in HIV-infected individuals, impairing their ability to control the virus and leading to worsened disease progression.

Furthermore, these findings highlight the need to consider the role of mitochondrial dysfunction in the context of HIV pathogenesis and the potential impact of fentanyl on HIV-related complications. Overall, the downregulation of MT-ATP8 observed in our study underscores the importance of mitochondrial health in the immune response to HIV and May inform therapeutic strategies aimed at mitigating these effects.

Enrichment analysis of single-cell RNA sequencing data in CD4^+^ T lymphocytes revealed heightened expression of genes related to crucial metabolic pathways such as oxidative phosphorylation, electron transport in ATP synthesis, and the electron transport chain, along with mitochondrial genes. This upregulation suggests increased energy metabolism in HIV-infected CD4^+^ T cells, potentially due to metabolic reprogramming to meet heightened energy demands. The enrichment of genes associated with oxidative phosphorylation implies an enhanced cellular energy production mechanism during HIV infection (Smeitink et al., 2001). Furthermore, the presence of enriched genes in electron transport for ATP synthesis indicates active ATP production via oxidative phosphorylation, crucial for various cellular functions including immune responses. Dysregulation of ATP synthesis pathways in HIV-infected CD4^+^ T cells May impact their functionality and survival (Ingwall and Ingwall, 2002). Additionally, the enrichment of mitochondrial genes suggests altered mitochondrial function, which plays essential roles in energy production, apoptosis regulation, and immune signaling. Dysfunction of mitochondria in HIV-infected CD4^+^ T cells May contribute to immune dysregulation and disease progression (Amand et al., 2023). Moreover, changes in gene expression related to the electron transport chain (ETC) May reflect alterations in mitochondrial function and cellular metabolism, potentially leading to reduced ATP production, oxidative stress, and compromised cell viability (Ramirez and Liu, 2018). Overall, these *in vitro* findings provide insights into the molecular mechanisms underlying HIV pathogenesis and offer potential therapeutic targets for restoring immune function and metabolic balance *in vivo*.

Fentanyl impacts immune signaling pathways, including interferon signaling and restriction factors, by binding to *μ*-opioid receptors on immune cells. This binding can suppress the production of interferons, which are crucial for antiviral defense (Dhawan et al., 1996). Fentanyl’s influence on interferon signaling can reduce the activation of interferon-stimulated genes (ISGs) that are essential for controlling viral replication (Cheon et al., 2013). Moreover, fentanyl May alter the expression or function of restriction factors—proteins that inhibit viral replication—by interfering with pathways such as the JAK–STAT pathway, which is critical for the interferon response (Schindler et al., 2007). This can weaken the immune system’s ability to restrict viral infections, leading to increased susceptibility to infections and impaired antiviral immunity. In the context of fentanyl-induced HIV pathogenesis, IFITM1, SERINC5, and TRIM5α play crucial roles as restriction factors that help the immune system combat viral infections, including HIV (Colomer-Lluch et al., 2018). In the presence of fentanyl, the suppression of interferon signaling can reduce the expression of IFITM1, weakening the cell’s ability to block HIV entry, potentially enhancing viral replication and spread. SERINC5 is a restriction factor that inhibits HIV-1 infectivity by incorporating into the viral envelope and interfering with the virus’s ability to infect new cells (Passos et al., 2019). In our study, we noted that fentanyl downregulated SERINC5 expression or altered its activity, that May lead to diminishing antiviral effects and facilitating the ability of HIV to infect and replicate within host cells. TRIM5α is a restriction factor that recognizes and targets the HIV capsid for degradation, preventing the virus from establishing infection (Stremlau et al., 2006; Laguette et al., 2011). Our results show decreased expression of TRIM5α in the presence of fentanyl that implies, fentanyl could potentially impair TRIM5α function by altering immune signaling pathways, leading to reduced degradation of the HIV capsid and allowing more efficient viral replication. These results suggest that fentanyl could modulate immune signaling pathways and potentially impact the expression or functionality of these proteins, thereby influencing HIV pathogenesis.

This study has several limitations worth noting. First, the sample size is relatively small, potentially limiting the generalizability of the findings. However, it is important to highlight that this study represents the first investigation into the impact of fentanyl on HIV-infected CD4^+^ T lymphocytes, and all five donors exhibited similar viral response patterns following fentanyl exposure. Second, the study utilized a single concentration of fentanyl, warranting further investigation into the varying effects on pathogenesis at different concentrations or levels matching those found in patient populations. Third, all experiments involving drug exposure and viral infection were conducted at a single time point, necessitating evaluation of the long-term effects of drug exposure on viral pathogenesis. Lastly, it is essential to acknowledge that all experiments were performed *ex vivo*. Future studies should aim to assess samples from HIV-positive individuals with and without drug exposure using single-cell RNAseq analysis across multiple distinct blood cell populations to provide a more comprehensive understanding of the observed effects.

In summary, our findings indicate that opioid use influences HIV pathogenesis through diverse mechanisms. To comprehensively understand the interactions among viruses, immune cells, and opioids, it is essential to characterize alterations in the peripheral blood transcriptome. This knowledge could not only improve clinical management approaches for challenging patient populations but also unveil novel therapeutic avenues.

## Data Availability

The datasets presented in this study can be found in online repositories. The names of the repository/repositories and accession number(s) can be found in the article/Supplementary material.
