# Supplementary material for: Effect of fentanyl on HIV expression in peripheral blood mononuclear cells
Source: Front Microbiol. 2024 Sep 25;15:1463441. doi: 10.3389/fmicb.2024.1463441 (PMC11461324; doi:10.3389/fmicb.2024.1463441)
Supplement: Supplementary file 1 [file Table_1.DOCX]

**Supplementary table 1:** List of differentially expressed genes in the CD4^+^ T lymphocytes infected with HIV:

| DEG's | p_val | avg_log2FC | pct.1 | pct.2 | p_val_adj |
| --- | --- | --- | --- | --- | --- |
| ABCB1 | 6.30E-147 | 0.922118 | 0.36 | 0.197 | 1.39E-142 |
| AL138963.4 | 5.81E-117 | 0.917569 | 0.529 | 0.415 | 1.28E-112 |
| AL133415.1 | 0 | 0.896048 | 0.9 | 0.477 | 0 |
| RASGEF1B | 5.41E-103 | 0.733849 | 0.284 | 0.151 | 1.19E-98 |
| BCOR | 9.53E-47 | 0.643097 | 0.273 | 0.2 | 2.10E-42 |
| MTRNR2L12 | 7.36E-186 | 0.546561 | 1 | 0.988 | 1.62E-181 |
| NDUFB1 | 8.84E-109 | 0.535544 | 0.992 | 0.769 | 1.95E-104 |
| RPS10 | 0.041167 | 0.509315 | 0.303 | 0.391 | 1 |
| ATP5ME | 3.14E-92 | 0.509268 | 0.992 | 0.802 | 6.92E-88 |
| COX17 | 1.40E-128 | 0.445055 | 0.955 | 0.638 | 3.08E-124 |
| EIF2S2 | 3.10E-147 | 0.44426 | 0.888 | 0.566 | 6.82E-143 |
| LPP | 0.006328 | 0.443882 | 0.256 | 0.298 | 1 |
| HSPD1 | 5.74E-148 | 0.425421 | 0.941 | 0.574 | 1.26E-143 |
| STMN1 | 0 | 0.418277 | 0.967 | 0.291 | 0 |
| FTH1 | 5.16E-114 | 0.417067 | 1 | 1 | 1.14E-109 |
| DEK | 1.37E-166 | 0.406866 | 0.968 | 0.572 | 3.02E-162 |
| C17orf49 | 1.20E-138 | 0.405261 | 0.948 | 0.583 | 2.65E-134 |
| NSA2 | 6.16E-103 | 0.393484 | 0.916 | 0.629 | 1.36E-98 |
| NUCKS1 | 1.98E-174 | 0.390408 | 0.875 | 0.504 | 4.35E-170 |
| TXN | 1.50E-47 | 0.384799 | 1 | 0.899 | 3.31E-43 |
| PA2G4 | 2.74E-89 | 0.380906 | 0.965 | 0.653 | 6.04E-85 |
| CAST | 9.56E-99 | 0.373901 | 0.972 | 0.642 | 2.11E-94 |
| PKM | 7.08E-80 | 0.349982 | 1 | 0.862 | 1.56E-75 |
| IK | 3.17E-68 | 0.347378 | 0.767 | 0.559 | 6.98E-64 |
| RNF213 | 2.95E-35 | 0.344145 | 0.998 | 0.83 | 6.50E-31 |
| RPS29 | 4.06E-132 | 0.34408 | 1 | 1 | 8.93E-128 |
| PDCD5 | 8.89E-103 | 0.343813 | 0.903 | 0.592 | 1.96E-98 |
| FLNA | 3.85E-101 | 0.337883 | 0.692 | 0.431 | 8.47E-97 |
| ST13 | 1.01E-70 | 0.325783 | 0.969 | 0.667 | 2.23E-66 |
| SLC26A3 | 2.41E-33 | 0.325478 | 0.116 | 0.058 | 5.31E-29 |
| SYF2 | 2.75E-63 | 0.325114 | 0.933 | 0.661 | 6.05E-59 |
| KTN1 | 6.99E-30 | 0.323756 | 0.988 | 0.767 | 1.54E-25 |
| TPM4 | 6.65E-116 | 0.322955 | 0.937 | 0.574 | 1.46E-111 |
| CD74 | 2.00E-152 | 0.322887 | 1 | 0.846 | 4.40E-148 |
| HMGB1 | 1.15E-57 | 0.321782 | 1 | 0.981 | 2.54E-53 |
| TPM3 | 4.56E-37 | 0.311757 | 0.999 | 0.89 | 1.00E-32 |
| LINC01943 | 4.54E-131 | 0.309761 | 0.812 | 0.454 | 9.99E-127 |
| HLA-DRB1 | 1.74E-185 | 0.309602 | 0.605 | 0.261 | 3.83E-181 |
| DDT | 1.97E-13 | 0.30277 | 0.541 | 0.503 | 4.34E-09 |
| ANXA5 | 1.90E-77 | 0.302552 | 0.983 | 0.647 | 4.19E-73 |
| DNAJA1 | 7.78E-66 | 0.298939 | 0.764 | 0.536 | 1.71E-61 |
| CCT3 | 2.73E-89 | 0.298585 | 0.842 | 0.547 | 6.02E-85 |
| SERBP1 | 3.56E-83 | 0.297932 | 0.96 | 0.624 | 7.83E-79 |
| HSP90AA1 | 5.31E-27 | 0.297553 | 1 | 0.901 | 1.17E-22 |
| GPI | 9.66E-99 | 0.296031 | 0.911 | 0.576 | 2.13E-94 |
| PHPT1 | 1.10E-110 | 0.293092 | 0.95 | 0.575 | 2.43E-106 |
| ROMO1 | 8.09E-16 | 0.29169 | 0.994 | 0.804 | 1.78E-11 |
| ENO1 | 1.46E-40 | 0.286817 | 1 | 0.871 | 3.22E-36 |
| NONO | 4.61E-82 | 0.285244 | 0.78 | 0.512 | 1.02E-77 |
| DNAJC15 | 2.06E-65 | 0.283128 | 0.786 | 0.549 | 4.53E-61 |
| GNB1 | 8.00E-67 | 0.282378 | 0.801 | 0.549 | 1.76E-62 |
| BOLA2B | 0.234526 | 0.281921 | 0.122 | 0.129 | 1 |
| AAK1 | 5.33E-54 | 0.279427 | 0.888 | 0.63 | 1.17E-49 |
| ZNHIT1 | 5.40E-69 | 0.277941 | 0.905 | 0.616 | 1.19E-64 |
| MRPL52 | 1.45E-56 | 0.277257 | 0.804 | 0.581 | 3.20E-52 |
| SRSF3 | 2.08E-101 | 0.273893 | 0.942 | 0.58 | 4.58E-97 |
| DNAJC8 | 2.65E-59 | 0.27325 | 0.795 | 0.562 | 5.82E-55 |
| KHDRBS1 | 9.11E-50 | 0.272991 | 0.986 | 0.677 | 2.01E-45 |
| LSM2 | 1.09E-102 | 0.271432 | 0.873 | 0.548 | 2.40E-98 |
| SEC11C | 5.90E-75 | 0.271127 | 0.957 | 0.626 | 1.30E-70 |
| RAD21 | 3.23E-93 | 0.270169 | 0.965 | 0.612 | 7.10E-89 |
| HSPB1 | 5.13E-61 | 0.268921 | 0.714 | 0.493 | 1.13E-56 |
| PET100 | 5.39E-25 | 0.268812 | 0.956 | 0.758 | 1.19E-20 |
| HSP90AB1 | 2.68E-11 | 0.266393 | 0.999 | 0.8 | 5.90E-07 |
| NOL7 | 1.88E-56 | 0.266179 | 0.768 | 0.549 | 4.13E-52 |
| KPNB1 | 9.79E-72 | 0.26268 | 0.784 | 0.528 | 2.16E-67 |
| HIST1H1E | 3.88E-107 | 0.261383 | 0.982 | 0.585 | 8.54E-103 |
| YWHAE | 3.39E-12 | 0.261243 | 0.571 | 0.516 | 7.46E-08 |
| IQGAP1 | 1.72E-71 | 0.260208 | 0.96 | 0.621 | 3.78E-67 |
| CCT4 | 1.83E-79 | 0.259834 | 0.913 | 0.598 | 4.02E-75 |
| GTF3A | 1.55E-74 | 0.259591 | 0.914 | 0.596 | 3.40E-70 |
| ITGAL | 5.25E-100 | 0.257994 | 0.787 | 0.478 | 1.16E-95 |
| S100A11 | 1.18E-54 | 0.257973 | 1 | 0.996 | 2.61E-50 |
| IRF1 | 3.98E-85 | 0.257336 | 0.764 | 0.476 | 8.75E-81 |
| DCTN3 | 6.22E-94 | 0.256763 | 0.941 | 0.59 | 1.37E-89 |
| ACAP2 | 3.74E-61 | 0.255302 | 0.806 | 0.551 | 8.25E-57 |
| C12orf75 | 5.69E-133 | 0.254494 | 0.908 | 0.496 | 1.25E-128 |
| EIF3A | 2.13E-76 | 0.251428 | 0.914 | 0.595 | 4.69E-72 |
| VIM | 1.32E-81 | 0.250433 | 1 | 0.991 | 2.91E-77 |
| VCP | 1.32E-61 | 0.250158 | 0.941 | 0.626 | 2.90E-57 |
| ECHDC2 | 2.84E-143 | -0.25001 | 0.029 | 0.244 | 6.26E-139 |
| CTBS | 4.63E-148 | -0.25004 | 0.045 | 0.281 | 1.02E-143 |
| DCAF6 | 4.94E-152 | -0.2501 | 0.045 | 0.285 | 1.09E-147 |
| BIRC3 | 1.01E-113 | -0.25011 | 0.045 | 0.239 | 2.23E-109 |
| ANKRD17 | 1.46E-159 | -0.25018 | 0.076 | 0.354 | 3.21E-155 |
| BABAM1 | 4.03E-179 | -0.2502 | 0.093 | 0.409 | 8.87E-175 |
| AKAP17A | 3.34E-142 | -0.25034 | 0.037 | 0.258 | 7.34E-138 |
| NOP58 | 7.00E-169 | -0.25045 | 0.082 | 0.374 | 1.54E-164 |
| TGOLN2 | 6.23E-175 | -0.25053 | 0.106 | 0.429 | 1.37E-170 |
| AATF | 2.89E-167 | -0.25067 | 0.06 | 0.331 | 6.36E-163 |
| CNST | 1.86E-154 | -0.25078 | 0.038 | 0.275 | 4.09E-150 |
| TIA1 | 1.14E-157 | -0.25079 | 0.049 | 0.3 | 2.51E-153 |
| PSMD6 | 6.16E-143 | -0.25082 | 0.181 | 0.467 | 1.36E-138 |
| ANAPC13 | 1.77E-156 | -0.25096 | 0.044 | 0.288 | 3.89E-152 |
| MED30 | 1.01E-167 | -0.251 | 0.055 | 0.323 | 2.23E-163 |
| ANKRD36 | 6.88E-149 | -0.25111 | 0.046 | 0.284 | 1.51E-144 |
| UBE2J2 | 1.15E-167 | -0.25122 | 0.053 | 0.319 | 2.53E-163 |
| RPL39 | 2.56E-142 | -0.25132 | 1 | 1 | 5.65E-138 |
| CIR1 | 4.55E-177 | -0.25139 | 0.082 | 0.385 | 1.00E-172 |
| EIF2AK1 | 1.26E-158 | -0.25148 | 0.057 | 0.316 | 2.77E-154 |
| CAPN7 | 1.16E-149 | -0.25153 | 0.036 | 0.264 | 2.56E-145 |
| CDC73 | 1.66E-166 | -0.25155 | 0.07 | 0.351 | 3.66E-162 |
| RILPL2 | 1.91E-176 | -0.25159 | 0.066 | 0.353 | 4.20E-172 |
| TMED5 | 4.63E-156 | -0.25165 | 0.041 | 0.283 | 1.02E-151 |
| SNHG32 | 3.89E-149 | -0.25173 | 0.06 | 0.311 | 8.57E-145 |
| LYST | 3.44E-143 | -0.25177 | 0.048 | 0.28 | 7.57E-139 |
| KLHL24 | 3.65E-149 | -0.25179 | 0.037 | 0.266 | 8.04E-145 |
| ATF7IP2 | 3.40E-143 | -0.25188 | 0.041 | 0.266 | 7.49E-139 |
| CUL5 | 8.57E-155 | -0.25191 | 0.04 | 0.278 | 1.89E-150 |
| GPBP1L1 | 5.41E-163 | -0.25196 | 0.055 | 0.318 | 1.19E-158 |
| CD59 | 4.30E-114 | -0.25216 | 0.064 | 0.274 | 9.47E-110 |
| TMEM126B | 4.39E-164 | -0.25232 | 0.055 | 0.319 | 9.67E-160 |
| CCT2 | 9.60E-165 | -0.25236 | 0.075 | 0.357 | 2.11E-160 |
| PIBF1 | 4.81E-149 | -0.25239 | 0.056 | 0.304 | 1.06E-144 |
| RIC1 | 1.48E-146 | -0.25249 | 0.039 | 0.267 | 3.25E-142 |
| OSBPL9 | 4.08E-166 | -0.2525 | 0.06 | 0.331 | 8.98E-162 |
| APMAP | 6.47E-175 | -0.25253 | 0.056 | 0.334 | 1.42E-170 |
| TRIM27 | 4.79E-167 | -0.25255 | 0.063 | 0.336 | 1.05E-162 |
| ICAM2 | 2.34E-145 | -0.25263 | 0.03 | 0.248 | 5.14E-141 |
| NSL1 | 3.83E-167 | -0.25272 | 0.06 | 0.332 | 8.44E-163 |
| PSMD2 | 1.04E-179 | -0.25274 | 0.091 | 0.406 | 2.29E-175 |
| GNPAT | 3.20E-150 | -0.25278 | 0.037 | 0.267 | 7.05E-146 |
| ANTXR2 | 1.67E-141 | -0.25283 | 0.045 | 0.272 | 3.68E-137 |
| NGLY1 | 7.34E-158 | -0.25295 | 0.047 | 0.295 | 1.62E-153 |
| PTPN1 | 1.10E-156 | -0.25298 | 0.057 | 0.313 | 2.43E-152 |
| EMC2 | 9.57E-155 | -0.25299 | 0.034 | 0.267 | 2.11E-150 |
| RALGAPA2 | 4.63E-147 | -0.25301 | 0.032 | 0.255 | 1.02E-142 |
| ACTR10 | 1.61E-178 | -0.25303 | 0.077 | 0.379 | 3.54E-174 |
| GPCPD1 | 1.46E-145 | -0.25307 | 0.034 | 0.256 | 3.21E-141 |
| KXD1 | 4.13E-152 | -0.25311 | 0.177 | 0.485 | 9.10E-148 |
| SBNO1 | 8.66E-158 | -0.25315 | 0.044 | 0.289 | 1.91E-153 |
| SPPL2A | 3.95E-166 | -0.25315 | 0.068 | 0.347 | 8.70E-162 |
| LDB1 | 3.09E-158 | -0.25322 | 0.042 | 0.287 | 6.81E-154 |
| PHACTR2 | 1.29E-76 | -0.25334 | 0.251 | 0.445 | 2.83E-72 |
| MRPS15 | 2.32E-194 | -0.25343 | 0.088 | 0.416 | 5.10E-190 |
| EBPL | 3.96E-158 | -0.25356 | 0.041 | 0.286 | 8.73E-154 |
| PRMT1 | 1.50E-180 | -0.25356 | 0.086 | 0.398 | 3.30E-176 |
| MRPL38 | 1.72E-180 | -0.25362 | 0.074 | 0.373 | 3.79E-176 |
| ATG3 | 7.70E-169 | -0.2537 | 0.061 | 0.335 | 1.69E-164 |
| MKNK2 | 6.02E-164 | -0.25377 | 0.056 | 0.321 | 1.33E-159 |
| C1orf122 | 4.29E-179 | -0.2538 | 0.073 | 0.371 | 9.44E-175 |
| PBX4 | 3.88E-160 | -0.25387 | 0.049 | 0.303 | 8.53E-156 |
| SEL1L3 | 2.96E-155 | -0.25395 | 0.054 | 0.307 | 6.52E-151 |
| LPAR6 | 1.94E-133 | -0.25401 | 0.026 | 0.226 | 4.26E-129 |
| ZFAND2B | 1.44E-172 | -0.25416 | 0.048 | 0.315 | 3.16E-168 |
| TRAV8-2 | 1.39E-62 | -0.25419 | 0.029 | 0.142 | 3.06E-58 |
| HOOK3 | 3.12E-153 | -0.2543 | 0.043 | 0.283 | 6.88E-149 |
| PFDN2 | 9.73E-204 | -0.25441 | 0.116 | 0.481 | 2.14E-199 |
| NUMA1 | 7.85E-155 | -0.25447 | 0.053 | 0.304 | 1.73E-150 |
| STAU1 | 1.70E-181 | -0.25469 | 0.079 | 0.386 | 3.74E-177 |
| DUS1L | 1.56E-171 | -0.25471 | 0.063 | 0.342 | 3.43E-167 |
| SNRNP35 | 8.75E-153 | -0.25474 | 0.038 | 0.272 | 1.93E-148 |
| FCMR | 7.53E-13 | -0.2548 | 0.489 | 0.483 | 1.66E-08 |
| MAN2B1 | 2.81E-151 | -0.25482 | 0.04 | 0.275 | 6.19E-147 |
| RPP21 | 1.76E-192 | -0.25488 | 0.102 | 0.442 | 3.87E-188 |
| PPIL4 | 3.11E-163 | -0.25491 | 0.045 | 0.297 | 6.84E-159 |
| POLR2C | 4.01E-169 | -0.25503 | 0.051 | 0.316 | 8.83E-165 |
| STOM | 3.87E-137 | -0.25516 | 0.036 | 0.25 | 8.51E-133 |
| CARD11 | 1.44E-155 | -0.25519 | 0.052 | 0.303 | 3.17E-151 |
| EMC4 | 8.97E-152 | -0.25543 | 0.18 | 0.479 | 1.98E-147 |
| USO1 | 3.52E-159 | -0.25551 | 0.053 | 0.309 | 7.75E-155 |
| SCFD1 | 8.19E-149 | -0.2556 | 0.047 | 0.285 | 1.80E-144 |
| ATP8A1 | 1.98E-136 | -0.25579 | 0.022 | 0.222 | 4.36E-132 |
| FDX1 | 1.06E-164 | -0.25581 | 0.058 | 0.324 | 2.33E-160 |
| TRAPPC4 | 1.94E-170 | -0.25583 | 0.059 | 0.333 | 4.27E-166 |
| UXS1 | 4.40E-159 | -0.25585 | 0.057 | 0.317 | 9.70E-155 |
| SNX14 | 1.54E-156 | -0.25585 | 0.049 | 0.299 | 3.40E-152 |
| NFATC2 | 1.65E-132 | -0.25593 | 0.162 | 0.432 | 3.64E-128 |
| EMC3 | 1.66E-178 | -0.25604 | 0.073 | 0.368 | 3.65E-174 |
| GTF2I | 2.29E-167 | -0.25606 | 0.108 | 0.424 | 5.04E-163 |
| NUDC | 6.14E-194 | -0.25622 | 0.1 | 0.439 | 1.35E-189 |
| AGGF1 | 1.32E-167 | -0.25638 | 0.063 | 0.338 | 2.91E-163 |
| LUC7L2 | 2.82E-182 | -0.25641 | 0.104 | 0.433 | 6.21E-178 |
| RRAS | 5.39E-173 | -0.25642 | 0.059 | 0.337 | 1.19E-168 |
| YARS | 1.06E-173 | -0.25656 | 0.118 | 0.449 | 2.33E-169 |
| RFXANK | 3.45E-164 | -0.25669 | 0.045 | 0.299 | 7.59E-160 |
| PKN2 | 3.11E-149 | -0.25676 | 0.039 | 0.269 | 6.85E-145 |
| TMEM80 | 4.97E-159 | -0.25677 | 0.031 | 0.265 | 1.09E-154 |
| PPP6R1 | 1.14E-177 | -0.25691 | 0.088 | 0.397 | 2.51E-173 |
| SELL | 2.05E-11 | -0.25708 | 0.98 | 0.591 | 4.52E-07 |
| FDPS | 1.20E-179 | -0.25712 | 0.091 | 0.405 | 2.65E-175 |
| ADIPOR1 | 4.83E-184 | -0.25714 | 0.07 | 0.369 | 1.06E-179 |
| NBR1 | 2.61E-167 | -0.25715 | 0.052 | 0.316 | 5.75E-163 |
| NMI | 1.00E-156 | -0.25721 | 0.044 | 0.288 | 2.21E-152 |
| SPPL3 | 4.29E-155 | -0.25723 | 0.037 | 0.271 | 9.44E-151 |
| GRK6 | 1.15E-157 | -0.25728 | 0.047 | 0.294 | 2.53E-153 |
| SNAPIN | 1.17E-177 | -0.25738 | 0.068 | 0.358 | 2.59E-173 |
| NCOA4 | 1.97E-186 | -0.25738 | 0.106 | 0.441 | 4.34E-182 |
| IBTK | 3.28E-146 | -0.25747 | 0.026 | 0.241 | 7.22E-142 |
| OGDH | 1.70E-160 | -0.25751 | 0.067 | 0.337 | 3.73E-156 |
| VMP1 | 8.82E-177 | -0.25757 | 0.107 | 0.433 | 1.94E-172 |
| TMEM87A | 6.98E-169 | -0.25757 | 0.068 | 0.349 | 1.54E-164 |
| C1GALT1 | 4.87E-156 | -0.25757 | 0.034 | 0.268 | 1.07E-151 |
| STAT5B | 1.55E-162 | -0.25759 | 0.057 | 0.32 | 3.41E-158 |
| STK17A | 1.33E-74 | -0.25761 | 0.994 | 0.859 | 2.93E-70 |
| TMEM131L | 1.25E-139 | -0.25769 | 0.024 | 0.23 | 2.76E-135 |
| EIF3F | 1.93E-114 | -0.25773 | 1 | 0.963 | 4.25E-110 |
| DIPK1A | 5.90E-145 | -0.25775 | 0.046 | 0.279 | 1.30E-140 |
| PPM1K | 3.90E-150 | -0.25808 | 0.04 | 0.273 | 8.58E-146 |
| GLB1 | 1.27E-156 | -0.25813 | 0.047 | 0.294 | 2.81E-152 |
| INTS10 | 3.36E-165 | -0.25813 | 0.045 | 0.3 | 7.39E-161 |
| PIGC | 5.35E-168 | -0.25815 | 0.045 | 0.303 | 1.18E-163 |
| MAEA | 8.68E-171 | -0.25819 | 0.065 | 0.345 | 1.91E-166 |
| ARRDC2 | 6.52E-153 | -0.25821 | 0.046 | 0.288 | 1.44E-148 |
| MRPL36 | 2.41E-192 | -0.2583 | 0.096 | 0.429 | 5.30E-188 |
| SPN | 3.90E-163 | -0.25831 | 0.084 | 0.373 | 8.59E-159 |
| CHD3 | 1.39E-162 | -0.25838 | 0.081 | 0.366 | 3.07E-158 |
| ZNF131 | 5.71E-153 | -0.25851 | 0.041 | 0.278 | 1.26E-148 |
| TAF7 | 6.28E-92 | -0.25866 | 0.284 | 0.493 | 1.38E-87 |
| SEC13 | 1.47E-192 | -0.2587 | 0.076 | 0.391 | 3.23E-188 |
| ZNF688 | 1.28E-160 | -0.25873 | 0.04 | 0.285 | 2.82E-156 |
| STMP1 | 1.36E-196 | -0.25876 | 0.094 | 0.429 | 3.00E-192 |
| TXNDC12 | 1.14E-169 | -0.25894 | 0.059 | 0.332 | 2.51E-165 |
| RAP1GDS1 | 1.72E-160 | -0.25895 | 0.061 | 0.325 | 3.80E-156 |
| DLG1 | 1.85E-153 | -0.25904 | 0.046 | 0.288 | 4.08E-149 |
| DDX3X | 9.05E-162 | -0.25909 | 0.08 | 0.362 | 1.99E-157 |
| CHCHD1 | 2.03E-198 | -0.25915 | 0.103 | 0.449 | 4.46E-194 |
| GLT8D1 | 5.39E-162 | -0.25919 | 0.027 | 0.259 | 1.19E-157 |
| WNK1 | 3.39E-142 | -0.25931 | 0.168 | 0.457 | 7.45E-138 |
| MCRS1 | 3.00E-165 | -0.25934 | 0.047 | 0.303 | 6.60E-161 |
| RNF113A | 1.66E-173 | -0.25948 | 0.048 | 0.314 | 3.66E-169 |
| PLCL2 | 2.58E-151 | -0.25963 | 0.049 | 0.292 | 5.69E-147 |
| BCAS3 | 6.23E-139 | -0.25969 | 0.036 | 0.252 | 1.37E-134 |
| KIF5B | 2.19E-147 | -0.25975 | 0.162 | 0.46 | 4.83E-143 |
| RALGAPA1 | 5.29E-148 | -0.25983 | 0.043 | 0.277 | 1.16E-143 |
| VSIR | 1.06E-145 | -0.25989 | 0.038 | 0.263 | 2.33E-141 |
| ERG28 | 6.76E-174 | -0.26012 | 0.07 | 0.359 | 1.49E-169 |
| CSGALNACT2 | 6.39E-153 | -0.26012 | 0.045 | 0.285 | 1.41E-148 |
| ZMYND11 | 1.36E-154 | -0.26014 | 0.033 | 0.263 | 3.00E-150 |
| LARP7 | 4.62E-158 | -0.26027 | 0.052 | 0.305 | 1.02E-153 |
| HIST3H2A | 1.04E-158 | -0.26055 | 0.055 | 0.313 | 2.29E-154 |
| MAP3K5 | 3.98E-143 | -0.26063 | 0.032 | 0.249 | 8.77E-139 |
| OTUD6B-AS1 | 7.18E-173 | -0.26063 | 0.043 | 0.305 | 1.58E-168 |
| JAGN1 | 2.79E-175 | -0.26075 | 0.049 | 0.319 | 6.14E-171 |
| DOCK11 | 6.32E-161 | -0.26075 | 0.051 | 0.307 | 1.39E-156 |
| TBK1 | 3.21E-160 | -0.26081 | 0.041 | 0.288 | 7.07E-156 |
| GMDS-DT | 3.01E-151 | -0.26094 | 0.057 | 0.308 | 6.64E-147 |
| LMAN1 | 2.26E-169 | -0.26115 | 0.075 | 0.363 | 4.97E-165 |
| CPOX | 2.81E-149 | -0.26116 | 0.071 | 0.331 | 6.20E-145 |
| MAN2A1 | 5.58E-139 | -0.26117 | 0.032 | 0.245 | 1.23E-134 |
| GOPC | 1.36E-164 | -0.26118 | 0.045 | 0.3 | 2.99E-160 |
| EMB | 4.76E-45 | -0.26121 | 0.989 | 0.794 | 1.05E-40 |
| ZC3H6 | 7.41E-149 | -0.26134 | 0.046 | 0.283 | 1.63E-144 |
| ANKRD49 | 6.06E-161 | -0.26152 | 0.044 | 0.294 | 1.34E-156 |
| TBC1D2B | 1.27E-155 | -0.26152 | 0.046 | 0.292 | 2.79E-151 |
| TIPRL | 4.12E-165 | -0.26159 | 0.051 | 0.311 | 9.07E-161 |
| DBNL | 8.88E-173 | -0.2617 | 0.065 | 0.348 | 1.95E-168 |
| PAIP1 | 5.86E-167 | -0.26172 | 0.037 | 0.285 | 1.29E-162 |
| SDHB | 2.54E-180 | -0.26178 | 0.061 | 0.349 | 5.60E-176 |
| TM9SF3 | 1.08E-164 | -0.2618 | 0.058 | 0.324 | 2.37E-160 |
| MAPK1 | 3.58E-177 | -0.26188 | 0.071 | 0.363 | 7.89E-173 |
| SPOCK2 | 2.66E-56 | -0.26191 | 0.992 | 0.824 | 5.86E-52 |
| IL12RB1 | 1.67E-150 | -0.26208 | 0.035 | 0.264 | 3.68E-146 |
| STIM1 | 5.01E-155 | -0.26212 | 0.04 | 0.278 | 1.10E-150 |
| DHPS | 2.86E-170 | -0.26218 | 0.05 | 0.315 | 6.30E-166 |
| CMC2 | 1.08E-181 | -0.26219 | 0.124 | 0.469 | 2.39E-177 |
| DIMT1 | 4.43E-167 | -0.26221 | 0.057 | 0.325 | 9.76E-163 |
| SMCO4 | 7.19E-125 | -0.26235 | 0.092 | 0.338 | 1.58E-120 |
| PHF20 | 2.44E-168 | -0.26255 | 0.064 | 0.341 | 5.38E-164 |
| CBLL1 | 8.72E-160 | -0.26256 | 0.047 | 0.298 | 1.92E-155 |
| PBDC1 | 2.06E-166 | -0.26262 | 0.043 | 0.298 | 4.55E-162 |
| DDHD1 | 6.51E-151 | -0.26287 | 0.043 | 0.279 | 1.43E-146 |
| SMARCC1 | 7.87E-161 | -0.26289 | 0.052 | 0.309 | 1.73E-156 |
| VPS72 | 6.00E-174 | -0.26291 | 0.052 | 0.322 | 1.32E-169 |
| RCN2 | 6.39E-192 | -0.26297 | 0.074 | 0.385 | 1.41E-187 |
| METTL9 | 1.37E-124 | -0.26307 | 0.23 | 0.49 | 3.01E-120 |
| VPS13A | 4.15E-172 | -0.26327 | 0.075 | 0.366 | 9.14E-168 |
| MTCH2 | 1.58E-181 | -0.26333 | 0.081 | 0.388 | 3.48E-177 |
| COMMD1 | 3.68E-179 | -0.26335 | 0.063 | 0.352 | 8.11E-175 |
| ABHD13 | 1.20E-159 | -0.26339 | 0.029 | 0.261 | 2.63E-155 |
| CTR9 | 2.68E-159 | -0.26362 | 0.045 | 0.293 | 5.90E-155 |
| DPY30 | 1.22E-196 | -0.26369 | 0.103 | 0.447 | 2.68E-192 |
| CLN3 | 3.17E-173 | -0.26378 | 0.043 | 0.306 | 6.98E-169 |
| MYL12A | 5.07E-97 | -0.2638 | 1 | 0.997 | 1.12E-92 |
| INO80E | 5.98E-180 | -0.2638 | 0.062 | 0.349 | 1.32E-175 |
| CAMK4 | 9.17E-39 | -0.26381 | 0.985 | 0.769 | 2.02E-34 |
| VPS13B | 7.23E-164 | -0.26385 | 0.066 | 0.339 | 1.59E-159 |
| FAM102B | 7.50E-149 | -0.26412 | 0.04 | 0.271 | 1.65E-144 |
| CDIPT | 4.02E-170 | -0.26416 | 0.048 | 0.312 | 8.85E-166 |
| RNF126 | 1.36E-166 | -0.26416 | 0.05 | 0.311 | 2.99E-162 |
| ARID5A | 5.17E-160 | -0.2642 | 0.063 | 0.33 | 1.14E-155 |
| SSBP2 | 5.06E-139 | -0.26428 | 0.037 | 0.254 | 1.11E-134 |
| PHIP | 9.60E-148 | -0.26439 | 0.075 | 0.336 | 2.11E-143 |
| WDR82 | 2.57E-169 | -0.26448 | 0.052 | 0.319 | 5.65E-165 |
| MXRA7 | 1.73E-152 | -0.26449 | 0.06 | 0.314 | 3.81E-148 |
| RHBDD2 | 4.47E-164 | -0.26449 | 0.049 | 0.306 | 9.85E-160 |
| RBM6 | 3.86E-174 | -0.26452 | 0.06 | 0.339 | 8.51E-170 |
| UQCRC1 | 3.48E-171 | -0.26468 | 0.123 | 0.433 | 7.66E-167 |
| SRSF5 | 1.60E-101 | -0.2647 | 0.994 | 0.89 | 3.53E-97 |
| UROS | 1.02E-172 | -0.2648 | 0.047 | 0.313 | 2.26E-168 |
| PJA2 | 8.23E-178 | -0.26481 | 0.071 | 0.363 | 1.81E-173 |
| ZNF414 | 1.84E-168 | -0.26482 | 0.043 | 0.3 | 4.05E-164 |
| HARS | 4.28E-162 | -0.26483 | 0.04 | 0.285 | 9.42E-158 |
| SPG7 | 5.37E-162 | -0.26489 | 0.043 | 0.293 | 1.18E-157 |
| DNAJC7 | 4.59E-183 | -0.265 | 0.074 | 0.377 | 1.01E-178 |
| ANKH | 2.87E-165 | -0.26503 | 0.074 | 0.356 | 6.32E-161 |
| INPP5D | 6.03E-155 | -0.26503 | 0.049 | 0.297 | 1.33E-150 |
| NCOA3 | 3.88E-161 | -0.26507 | 0.063 | 0.331 | 8.54E-157 |
| MAP3K2 | 1.36E-168 | -0.26521 | 0.078 | 0.367 | 3.00E-164 |
| BIRC2 | 2.57E-158 | -0.26531 | 0.075 | 0.351 | 5.67E-154 |
| MIF4GD | 1.88E-176 | -0.26541 | 0.057 | 0.336 | 4.14E-172 |
| RNF34 | 3.80E-174 | -0.26552 | 0.058 | 0.336 | 8.36E-170 |
| STN1 | 3.89E-170 | -0.2656 | 0.057 | 0.329 | 8.57E-166 |
| CFAP36 | 3.89E-174 | -0.26571 | 0.059 | 0.337 | 8.56E-170 |
| SSB | 9.69E-196 | -0.26572 | 0.091 | 0.424 | 2.13E-191 |
| SEC22C | 4.01E-163 | -0.26572 | 0.045 | 0.297 | 8.82E-159 |
| NDUFA10 | 5.89E-187 | -0.26577 | 0.083 | 0.398 | 1.30E-182 |
| ZFP36L2 | 1.08E-23 | -0.26578 | 0.983 | 0.733 | 2.38E-19 |
| PPP3CB | 3.82E-159 | -0.2659 | 0.038 | 0.28 | 8.41E-155 |
| SEMA4D | 8.22E-162 | -0.26603 | 0.05 | 0.306 | 1.81E-157 |
| PSTPIP1 | 4.42E-187 | -0.26624 | 0.093 | 0.418 | 9.73E-183 |
| PNP | 2.47E-175 | -0.26624 | 0.063 | 0.347 | 5.43E-171 |
| RRP36 | 3.36E-176 | -0.26625 | 0.06 | 0.341 | 7.41E-172 |
| MAP3K1 | 1.47E-142 | -0.26625 | 0.028 | 0.24 | 3.24E-138 |
| CD7 | 0.025111 | -0.26627 | 0.985 | 0.671 | 1 |
| PPM1M | 1.11E-158 | -0.26629 | 0.04 | 0.283 | 2.43E-154 |
| CAMTA1 | 3.92E-190 | -0.26638 | 0.083 | 0.402 | 8.63E-186 |
| ATP6V1D | 2.92E-165 | -0.2664 | 0.037 | 0.283 | 6.43E-161 |
| MAP4K4 | 2.20E-166 | -0.26642 | 0.071 | 0.351 | 4.85E-162 |
| AK3 | 1.49E-155 | -0.26648 | 0.041 | 0.281 | 3.28E-151 |
| TAF12 | 1.63E-176 | -0.26651 | 0.049 | 0.321 | 3.59E-172 |
| ETNK1 | 2.13E-173 | -0.26651 | 0.047 | 0.313 | 4.69E-169 |
| MRPL4 | 5.12E-192 | -0.26656 | 0.07 | 0.378 | 1.13E-187 |
| 2-Mar | 3.67E-181 | -0.26664 | 0.057 | 0.342 | 8.08E-177 |
| MANF | 1.76E-167 | -0.26667 | 0.066 | 0.344 | 3.88E-163 |
| SMIM27 | 3.75E-180 | -0.26708 | 0.079 | 0.383 | 8.26E-176 |
| GSK3B | 6.90E-158 | -0.26709 | 0.041 | 0.284 | 1.52E-153 |
| G3BP1 | 6.90E-184 | -0.26711 | 0.102 | 0.43 | 1.52E-179 |
| NCF1 | 1.05E-149 | -0.26713 | 0.071 | 0.332 | 2.32E-145 |
| CDK9 | 1.59E-173 | -0.26725 | 0.047 | 0.314 | 3.51E-169 |
| JAZF1 | 3.93E-140 | -0.26728 | 0.025 | 0.231 | 8.66E-136 |
| CHMP7 | 9.51E-156 | -0.26728 | 0.037 | 0.273 | 2.09E-151 |
| GID8 | 3.35E-185 | -0.26735 | 0.062 | 0.355 | 7.38E-181 |
| RO60 | 1.93E-171 | -0.26748 | 0.046 | 0.31 | 4.24E-167 |
| KCMF1 | 3.29E-180 | -0.26748 | 0.057 | 0.339 | 7.25E-176 |
| CLK3 | 8.04E-169 | -0.26772 | 0.042 | 0.299 | 1.77E-164 |
| CD46 | 3.08E-206 | -0.26774 | 0.104 | 0.459 | 6.79E-202 |
| RIC3 | 5.79E-144 | -0.26774 | 0.019 | 0.224 | 1.28E-139 |
| SCAMP3 | 1.08E-192 | -0.26776 | 0.079 | 0.398 | 2.39E-188 |
| A1BG | 2.12E-195 | -0.26779 | 0.13 | 0.497 | 4.66E-191 |
| GIMAP7 | 1.41E-19 | -0.26791 | 0.98 | 0.728 | 3.11E-15 |
| BAZ2A | 1.89E-166 | -0.268 | 0.045 | 0.302 | 4.16E-162 |
| GGNBP2 | 1.29E-182 | -0.26809 | 0.086 | 0.399 | 2.83E-178 |
| RALA | 6.58E-177 | -0.26821 | 0.129 | 0.454 | 1.45E-172 |
| VPS35 | 1.73E-184 | -0.26829 | 0.081 | 0.391 | 3.80E-180 |
| RNF125 | 3.91E-166 | -0.26829 | 0.031 | 0.273 | 8.60E-162 |
| DRAM2 | 7.86E-159 | -0.26834 | 0.03 | 0.262 | 1.73E-154 |
| UFD1 | 2.69E-181 | -0.2684 | 0.078 | 0.382 | 5.92E-177 |
| SNRK | 4.39E-155 | -0.26842 | 0.026 | 0.251 | 9.67E-151 |
| WIPI2 | 1.63E-171 | -0.26858 | 0.057 | 0.329 | 3.58E-167 |
| ZNF22 | 2.88E-168 | -0.26859 | 0.038 | 0.288 | 6.33E-164 |
| ANK3 | 1.01E-129 | -0.2686 | 0.082 | 0.327 | 2.22E-125 |
| IVNS1ABP | 7.60E-140 | -0.26865 | 0.114 | 0.397 | 1.67E-135 |
| SNX20 | 3.45E-170 | -0.26871 | 0.039 | 0.293 | 7.60E-166 |
| TPGS1 | 8.32E-199 | -0.26872 | 0.079 | 0.405 | 1.83E-194 |
| ZFAND3 | 2.53E-173 | -0.26879 | 0.073 | 0.362 | 5.58E-169 |
| HCFC1R1 | 2.04E-193 | -0.26884 | 0.096 | 0.429 | 4.50E-189 |
| NDUFA9 | 9.22E-181 | -0.26886 | 0.066 | 0.357 | 2.03E-176 |
| CNIH4 | 1.16E-165 | -0.2689 | 0.07 | 0.348 | 2.55E-161 |
| ANKRD10 | 1.63E-178 | -0.26893 | 0.071 | 0.364 | 3.59E-174 |
| SECISBP2L | 4.25E-174 | -0.26894 | 0.082 | 0.381 | 9.35E-170 |
| CPSF6 | 5.86E-178 | -0.26896 | 0.067 | 0.357 | 1.29E-173 |
| PTOV1 | 8.67E-176 | -0.26908 | 0.06 | 0.34 | 1.91E-171 |
| ZNF24 | 7.55E-170 | -0.26918 | 0.045 | 0.305 | 1.66E-165 |
| LIX1L | 1.31E-167 | -0.2692 | 0.047 | 0.306 | 2.89E-163 |
| TINF2 | 2.44E-167 | -0.26933 | 0.05 | 0.311 | 5.36E-163 |
| NUBP2 | 9.14E-186 | -0.26935 | 0.062 | 0.356 | 2.01E-181 |
| PHF5A | 5.06E-184 | -0.26947 | 0.069 | 0.368 | 1.11E-179 |
| ACIN1 | 2.99E-171 | -0.26949 | 0.086 | 0.386 | 6.58E-167 |
| PQBP1 | 9.59E-188 | -0.26958 | 0.083 | 0.399 | 2.11E-183 |
| ABHD14A | 7.16E-169 | -0.26962 | 0.041 | 0.297 | 1.58E-164 |
| AC245407.2 | 3.20E-146 | -0.26972 | 0.017 | 0.222 | 7.04E-142 |
| MPV17 | 3.30E-188 | -0.26974 | 0.084 | 0.401 | 7.27E-184 |
| CDK5RAP3 | 8.05E-188 | -0.2698 | 0.079 | 0.392 | 1.77E-183 |
| CLTB | 7.96E-125 | -0.26982 | 0.233 | 0.494 | 1.75E-120 |
| GOLPH3 | 3.88E-167 | -0.26985 | 0.051 | 0.313 | 8.55E-163 |
| ACTB | 3.33E-108 | -0.26987 | 1 | 1 | 7.34E-104 |
| ZNF862 | 7.71E-163 | -0.26997 | 0.028 | 0.262 | 1.70E-158 |
| PHF23 | 1.13E-173 | -0.27005 | 0.041 | 0.303 | 2.48E-169 |
| RASA1 | 9.21E-158 | -0.27007 | 0.037 | 0.275 | 2.03E-153 |
| MGAT1 | 4.22E-178 | -0.27007 | 0.055 | 0.335 | 9.30E-174 |
| SLC7A6 | 4.85E-153 | -0.27037 | 0.044 | 0.284 | 1.07E-148 |
| ERCC1 | 4.00E-171 | -0.27041 | 0.064 | 0.343 | 8.80E-167 |
| ALKBH5 | 8.16E-170 | -0.27041 | 0.038 | 0.292 | 1.80E-165 |
| RPS4Y1 | 2.42E-135 | -0.27044 | 1 | 0.989 | 5.33E-131 |
| CCDC90B | 9.33E-185 | -0.27061 | 0.072 | 0.374 | 2.05E-180 |
| SPOP | 3.63E-160 | -0.27063 | 0.037 | 0.276 | 7.99E-156 |
| FAM104A | 3.10E-184 | -0.27064 | 0.083 | 0.396 | 6.83E-180 |
| ORAI3 | 1.29E-162 | -0.27076 | 0.052 | 0.31 | 2.85E-158 |
| WASHC4 | 6.87E-169 | -0.27088 | 0.054 | 0.322 | 1.51E-164 |
| GMIP | 3.68E-172 | -0.27095 | 0.051 | 0.319 | 8.09E-168 |
| CERS2 | 3.35E-181 | -0.27098 | 0.064 | 0.354 | 7.37E-177 |
| ELK3 | 5.98E-175 | -0.27114 | 0.073 | 0.366 | 1.32E-170 |
| HNRNPH2 | 3.47E-173 | -0.27115 | 0.056 | 0.33 | 7.63E-169 |
| MARK3 | 4.93E-167 | -0.27122 | 0.056 | 0.322 | 1.08E-162 |
| NRBP1 | 1.71E-189 | -0.27128 | 0.071 | 0.377 | 3.77E-185 |
| CYB561D2 | 3.83E-177 | -0.27131 | 0.04 | 0.304 | 8.44E-173 |
| STARD3 | 5.04E-166 | -0.27141 | 0.045 | 0.3 | 1.11E-161 |
| CREBZF | 1.71E-167 | -0.27149 | 0.034 | 0.28 | 3.76E-163 |
| CTNNB1 | 7.14E-173 | -0.27157 | 0.061 | 0.339 | 1.57E-168 |
| LBR | 1.29E-176 | -0.27163 | 0.088 | 0.395 | 2.84E-172 |
| UBL3 | 3.21E-157 | -0.2717 | 0.036 | 0.273 | 7.07E-153 |
| INPP4A | 2.76E-165 | -0.27183 | 0.048 | 0.305 | 6.09E-161 |
| BCL7B | 5.77E-184 | -0.27195 | 0.053 | 0.338 | 1.27E-179 |
| MAPRE1 | 1.28E-176 | -0.27216 | 0.063 | 0.346 | 2.82E-172 |
| GNG10 | 6.31E-168 | -0.27228 | 0.044 | 0.3 | 1.39E-163 |
| DENND1C | 9.66E-177 | -0.27231 | 0.055 | 0.332 | 2.13E-172 |
| GRN | 6.36E-174 | -0.27239 | 0.058 | 0.336 | 1.40E-169 |
| CYC1 | 1.05E-197 | -0.27246 | 0.085 | 0.413 | 2.30E-193 |
| PPP6R3 | 6.05E-172 | -0.27249 | 0.058 | 0.332 | 1.33E-167 |
| JKAMP | 3.94E-188 | -0.27251 | 0.057 | 0.347 | 8.67E-184 |
| MAPK1IP1L | 1.37E-193 | -0.27253 | 0.076 | 0.391 | 3.02E-189 |
| EIF2A | 8.84E-180 | -0.27271 | 0.063 | 0.352 | 1.95E-175 |
| UBE2R2 | 1.10E-154 | -0.27272 | 0.158 | 0.456 | 2.42E-150 |
| MYO5A | 5.17E-163 | -0.27279 | 0.057 | 0.321 | 1.14E-158 |
| AFTPH | 2.28E-164 | -0.27282 | 0.053 | 0.313 | 5.02E-160 |
| MRPS7 | 5.62E-183 | -0.27286 | 0.058 | 0.344 | 1.24E-178 |
| KANSL1 | 1.12E-172 | -0.27295 | 0.063 | 0.343 | 2.47E-168 |
| SNF8 | 5.86E-190 | -0.27295 | 0.079 | 0.394 | 1.29E-185 |
| CAT | 1.70E-171 | -0.27298 | 0.047 | 0.311 | 3.74E-167 |
| ZFAND1 | 1.71E-167 | -0.27306 | 0.045 | 0.302 | 3.75E-163 |
| SDR39U1 | 1.31E-164 | -0.27308 | 0.028 | 0.265 | 2.89E-160 |
| AKAP8L | 2.59E-182 | -0.27314 | 0.065 | 0.358 | 5.69E-178 |
| MRPL16 | 7.30E-207 | -0.27325 | 0.086 | 0.425 | 1.61E-202 |
| IER3IP1 | 7.88E-198 | -0.27333 | 0.081 | 0.406 | 1.73E-193 |
| MAP4K2 | 3.94E-165 | -0.27338 | 0.045 | 0.299 | 8.67E-161 |
| HDAC2 | 8.84E-175 | -0.2734 | 0.058 | 0.335 | 1.95E-170 |
| TCIRG1 | 2.57E-180 | -0.27344 | 0.059 | 0.344 | 5.65E-176 |
| PYHIN1 | 4.31E-92 | -0.2735 | 0.249 | 0.466 | 9.49E-88 |
| DOCK2 | 1.00E-172 | -0.27354 | 0.085 | 0.387 | 2.21E-168 |
| CYB5B | 9.16E-179 | -0.27357 | 0.061 | 0.346 | 2.02E-174 |
| FXR1 | 3.66E-186 | -0.27359 | 0.094 | 0.418 | 8.07E-182 |
| ESYT2 | 2.33E-164 | -0.27372 | 0.046 | 0.302 | 5.12E-160 |
| NORAD | 1.55E-156 | -0.27375 | 0.037 | 0.273 | 3.41E-152 |
| ERCC5 | 2.73E-175 | -0.27375 | 0.058 | 0.336 | 6.01E-171 |
| PPP1R21 | 3.09E-161 | -0.27381 | 0.046 | 0.297 | 6.80E-157 |
| TIMP1 | 8.59E-108 | -0.27394 | 0.1 | 0.327 | 1.89E-103 |
| VPS4B | 1.21E-187 | -0.27435 | 0.083 | 0.398 | 2.67E-183 |
| TSEN15 | 1.25E-186 | -0.27442 | 0.083 | 0.397 | 2.74E-182 |
| USP48 | 4.20E-168 | -0.27468 | 0.054 | 0.32 | 9.25E-164 |
| C12orf10 | 7.06E-171 | -0.2747 | 0.04 | 0.295 | 1.56E-166 |
| ARL14EP | 9.99E-172 | -0.2748 | 0.049 | 0.315 | 2.20E-167 |
| LSM1 | 1.08E-178 | -0.27494 | 0.069 | 0.36 | 2.38E-174 |
| VKORC1 | 2.50E-192 | -0.27495 | 0.088 | 0.414 | 5.50E-188 |
| TTC1 | 4.53E-188 | -0.27527 | 0.08 | 0.394 | 9.97E-184 |
| TCTN3 | 3.74E-111 | -0.27535 | 0.066 | 0.273 | 8.23E-107 |
| PRKCB | 1.80E-160 | -0.27538 | 0.049 | 0.302 | 3.97E-156 |
| GDI1 | 1.29E-183 | -0.27551 | 0.069 | 0.367 | 2.84E-179 |
| RNF5 | 2.43E-178 | -0.27553 | 0.063 | 0.349 | 5.35E-174 |
| AMZ2 | 2.07E-181 | -0.27556 | 0.058 | 0.342 | 4.57E-177 |
| ST8SIA4 | 1.33E-160 | -0.27558 | 0.049 | 0.301 | 2.92E-156 |
| ARHGAP30 | 1.14E-161 | -0.27575 | 0.127 | 0.423 | 2.51E-157 |
| LYPLA1 | 1.95E-179 | -0.27583 | 0.067 | 0.358 | 4.28E-175 |
| TRANK1 | 8.44E-169 | -0.27589 | 0.047 | 0.308 | 1.86E-164 |
| PSMA4 | 2.88E-200 | -0.27596 | 0.106 | 0.457 | 6.35E-196 |
| PAM | 1.19E-159 | -0.27602 | 0.042 | 0.288 | 2.62E-155 |
| CUEDC2 | 1.10E-167 | -0.27629 | 0.147 | 0.461 | 2.42E-163 |
| HDDC2 | 1.42E-178 | -0.27665 | 0.069 | 0.361 | 3.12E-174 |
| ZNF302 | 1.91E-159 | -0.27668 | 0.024 | 0.252 | 4.20E-155 |
| JAML | 1.13E-22 | -0.27683 | 0.994 | 0.767 | 2.48E-18 |
| ZBTB7A | 1.10E-180 | -0.2769 | 0.083 | 0.389 | 2.41E-176 |
| RPS15 | 2.49E-211 | -0.27696 | 1 | 1 | 5.48E-207 |
| LETMD1 | 1.14E-172 | -0.27696 | 0.047 | 0.311 | 2.52E-168 |
| SDCCAG8 | 4.46E-160 | -0.27721 | 0.036 | 0.276 | 9.81E-156 |
| RALGDS | 3.76E-176 | -0.2773 | 0.07 | 0.36 | 8.27E-172 |
| GON4L | 4.54E-176 | -0.27758 | 0.047 | 0.315 | 9.99E-172 |
| BCLAF1 | 6.94E-162 | -0.27798 | 0.182 | 0.502 | 1.53E-157 |
| MRPL3 | 3.84E-180 | -0.27806 | 0.051 | 0.327 | 8.45E-176 |
| AKR7A2 | 2.62E-186 | -0.27814 | 0.056 | 0.345 | 5.78E-182 |
| UFM1 | 1.59E-192 | -0.27815 | 0.097 | 0.429 | 3.51E-188 |
| KLF13 | 1.16E-184 | -0.2782 | 0.079 | 0.388 | 2.56E-180 |
| PDHB | 1.15E-178 | -0.27822 | 0.051 | 0.326 | 2.52E-174 |
| COPA | 4.33E-183 | -0.2783 | 0.065 | 0.359 | 9.53E-179 |
| HECTD1 | 1.89E-174 | -0.27834 | 0.058 | 0.336 | 4.15E-170 |
| SP110 | 1.55E-181 | -0.27837 | 0.079 | 0.383 | 3.41E-177 |
| ZNF721 | 1.93E-176 | -0.27837 | 0.06 | 0.341 | 4.25E-172 |
| UBASH3A | 3.55E-158 | -0.27844 | 0.031 | 0.265 | 7.83E-154 |
| ARID2 | 1.44E-164 | -0.27848 | 0.046 | 0.3 | 3.17E-160 |
| RSBN1L | 3.81E-191 | -0.27853 | 0.068 | 0.373 | 8.38E-187 |
| AKIRIN1 | 1.99E-176 | -0.27861 | 0.047 | 0.316 | 4.39E-172 |
| ZNF280D | 1.16E-166 | -0.27862 | 0.047 | 0.305 | 2.55E-162 |
| FLOT1 | 7.25E-158 | -0.27862 | 0.079 | 0.355 | 1.60E-153 |
| SHARPIN | 1.67E-180 | -0.2787 | 0.053 | 0.332 | 3.67E-176 |
| DUSP11 | 7.95E-189 | -0.2788 | 0.054 | 0.342 | 1.75E-184 |
| CCDC57 | 1.41E-179 | -0.27886 | 0.053 | 0.331 | 3.10E-175 |
| RAB4B | 1.96E-182 | -0.27887 | 0.064 | 0.356 | 4.31E-178 |
| INTS8 | 1.58E-163 | -0.27888 | 0.025 | 0.258 | 3.48E-159 |
| CTSS | 2.60E-181 | -0.27901 | 0.067 | 0.36 | 5.73E-177 |
| ZBTB1 | 1.02E-167 | -0.27922 | 0.051 | 0.314 | 2.24E-163 |
| VTI1A | 7.47E-168 | -0.27923 | 0.051 | 0.314 | 1.65E-163 |
| AKR1A1 | 7.14E-190 | -0.27924 | 0.07 | 0.376 | 1.57E-185 |
| CYBC1 | 3.63E-195 | -0.27925 | 0.081 | 0.403 | 8.00E-191 |
| TAGAP | 1.02E-137 | -0.27932 | 0.026 | 0.231 | 2.25E-133 |
| MRPL13 | 2.34E-187 | -0.27935 | 0.065 | 0.363 | 5.16E-183 |
| UBXN11 | 3.63E-150 | -0.27936 | 0.035 | 0.263 | 8.00E-146 |
| PHF1 | 4.83E-168 | -0.2794 | 0.042 | 0.298 | 1.06E-163 |
| RPS13 | 5.92E-149 | -0.27948 | 1 | 1 | 1.30E-144 |
| FBL | 2.64E-189 | -0.27955 | 0.078 | 0.391 | 5.82E-185 |
| TRAPPC10 | 5.15E-177 | -0.27958 | 0.039 | 0.3 | 1.13E-172 |
| SATB1-AS1 | 1.21E-148 | -0.27965 | 0.031 | 0.255 | 2.66E-144 |
| FIP1L1 | 3.60E-192 | -0.27982 | 0.073 | 0.384 | 7.93E-188 |
| NFKB1 | 6.15E-177 | -0.28009 | 0.083 | 0.385 | 1.35E-172 |
| CNOT8 | 7.14E-177 | -0.28032 | 0.043 | 0.31 | 1.57E-172 |
| FAM200B | 1.16E-177 | -0.28035 | 0.057 | 0.337 | 2.56E-173 |
| EIF1AY | 1.95E-186 | -0.28043 | 0.075 | 0.383 | 4.30E-182 |
| XBP1 | 2.25E-74 | -0.28044 | 0.278 | 0.46 | 4.95E-70 |
| EFR3A | 3.25E-169 | -0.28053 | 0.045 | 0.304 | 7.16E-165 |
| LINC01138 | 1.39E-195 | -0.28054 | 0.15 | 0.53 | 3.05E-191 |
| MRPL11 | 8.16E-197 | -0.28072 | 0.084 | 0.41 | 1.80E-192 |
| XPC | 4.46E-164 | -0.28103 | 0.057 | 0.321 | 9.82E-160 |
| COMMD10 | 5.31E-167 | -0.28117 | 0.038 | 0.288 | 1.17E-162 |
| HTATIP2 | 1.98E-159 | -0.28123 | 0.186 | 0.501 | 4.37E-155 |
| ZFR | 4.13E-179 | -0.28129 | 0.069 | 0.361 | 9.10E-175 |
| SUMF2 | 6.22E-177 | -0.28139 | 0.049 | 0.321 | 1.37E-172 |
| RGL4 | 3.20E-145 | -0.28143 | 0.026 | 0.24 | 7.04E-141 |
| MYDGF | 3.85E-207 | -0.28173 | 0.105 | 0.456 | 8.49E-203 |
| WAPL | 1.60E-177 | -0.28174 | 0.067 | 0.357 | 3.53E-173 |
| NLRC5 | 3.62E-168 | -0.28175 | 0.052 | 0.317 | 7.97E-164 |
| NPEPPS | 3.14E-186 | -0.28176 | 0.079 | 0.388 | 6.91E-182 |
| 7-Mar | 2.31E-184 | -0.28185 | 0.059 | 0.349 | 5.08E-180 |
| BBX | 5.65E-176 | -0.28195 | 0.077 | 0.375 | 1.24E-171 |
| SLC25A46 | 6.09E-163 | -0.282 | 0.039 | 0.285 | 1.34E-158 |
| DLEU2 | 2.59E-119 | -0.28206 | 0.127 | 0.389 | 5.69E-115 |
| NARF | 1.71E-182 | -0.28231 | 0.081 | 0.389 | 3.76E-178 |
| UBR5 | 4.60E-167 | -0.28231 | 0.047 | 0.307 | 1.01E-162 |
| DYNLT3 | 9.93E-187 | -0.28265 | 0.055 | 0.342 | 2.19E-182 |
| CYCS | 3.29E-185 | -0.28267 | 0.083 | 0.395 | 7.24E-181 |
| FAM162A | 1.30E-198 | -0.28275 | 0.091 | 0.426 | 2.87E-194 |
| SNX9 | 5.51E-160 | -0.28284 | 0.087 | 0.374 | 1.21E-155 |
| HADHB | 7.06E-186 | -0.28291 | 0.063 | 0.356 | 1.55E-181 |
| C5orf56 | 4.92E-179 | -0.28303 | 0.069 | 0.362 | 1.08E-174 |
| CD2BP2 | 8.84E-193 | -0.28312 | 0.051 | 0.341 | 1.95E-188 |
| ZBTB25 | 4.72E-169 | -0.28316 | 0.036 | 0.285 | 1.04E-164 |
| MTHFS | 8.17E-181 | -0.28326 | 0.064 | 0.355 | 1.80E-176 |
| RPL21 | 5.12E-144 | -0.2833 | 1 | 1 | 1.13E-139 |
| NUDT16L1 | 2.16E-191 | -0.28336 | 0.054 | 0.346 | 4.75E-187 |
| SETD5 | 1.15E-166 | -0.28365 | 0.053 | 0.317 | 2.54E-162 |
| RPL27A | 2.72E-102 | -0.2837 | 1 | 0.987 | 5.99E-98 |
| PSMB7 | 1.73E-205 | -0.28376 | 0.098 | 0.447 | 3.81E-201 |
| NCBP2AS2 | 3.72E-193 | -0.28384 | 0.05 | 0.341 | 8.20E-189 |
| MDFIC | 4.59E-183 | -0.28407 | 0.109 | 0.442 | 1.01E-178 |
| DOK2 | 1.87E-163 | -0.28422 | 0.032 | 0.273 | 4.13E-159 |
| CCDC88C | 1.35E-183 | -0.28423 | 0.063 | 0.356 | 2.98E-179 |
| PPM1B | 8.00E-173 | -0.28426 | 0.046 | 0.308 | 1.76E-168 |
| TASOR | 1.16E-190 | -0.28428 | 0.072 | 0.381 | 2.55E-186 |
| KMT2C | 2.26E-173 | -0.28441 | 0.064 | 0.345 | 4.97E-169 |
| DEXI | 1.07E-179 | -0.28466 | 0.032 | 0.289 | 2.36E-175 |
| PTPN4 | 6.65E-163 | -0.28473 | 0.046 | 0.299 | 1.46E-158 |
| NDUFA8 | 4.61E-219 | -0.28489 | 0.095 | 0.455 | 1.01E-214 |
| SURF1 | 3.73E-193 | -0.28489 | 0.063 | 0.365 | 8.21E-189 |
| LINC00861 | 3.90E-139 | -0.28492 | 0.027 | 0.233 | 8.59E-135 |
| HDAC3 | 7.09E-180 | -0.28511 | 0.045 | 0.316 | 1.56E-175 |
| ARHGEF6 | 1.58E-123 | -0.28522 | 0.216 | 0.476 | 3.47E-119 |
| AKAP9 | 7.21E-177 | -0.28524 | 0.132 | 0.465 | 1.59E-172 |
| ERO1A | 2.16E-177 | -0.28543 | 0.04 | 0.303 | 4.75E-173 |
| JPX | 1.43E-178 | -0.28544 | 0.038 | 0.3 | 3.14E-174 |
| UQCRHL | 4.70E-159 | -0.28545 | 0.004 | 0.207 | 1.03E-154 |
| SMAD3 | 2.80E-170 | -0.28564 | 0.038 | 0.292 | 6.17E-166 |
| SPG11 | 5.38E-164 | -0.2858 | 0.038 | 0.283 | 1.19E-159 |
| TPP2 | 7.67E-167 | -0.28611 | 0.035 | 0.282 | 1.69E-162 |
| STX10 | 4.83E-185 | -0.28618 | 0.045 | 0.322 | 1.06E-180 |
| MRPS18B | 1.11E-205 | -0.28626 | 0.088 | 0.426 | 2.44E-201 |
| PTEN | 7.62E-180 | -0.28635 | 0.054 | 0.334 | 1.68E-175 |
| DDRGK1 | 8.95E-192 | -0.28648 | 0.059 | 0.356 | 1.97E-187 |
| AP1S2 | 7.40E-185 | -0.28683 | 0.079 | 0.387 | 1.63E-180 |
| TRIP12 | 6.88E-181 | -0.28692 | 0.067 | 0.359 | 1.51E-176 |
| KIAA1109 | 9.31E-181 | -0.28695 | 0.052 | 0.331 | 2.05E-176 |
| ZMAT2 | 2.07E-209 | -0.28697 | 0.1 | 0.455 | 4.56E-205 |
| LNPEP | 2.54E-118 | -0.287 | 0.236 | 0.491 | 5.59E-114 |
| MED15 | 3.05E-200 | -0.28704 | 0.073 | 0.394 | 6.71E-196 |
| DGCR6L | 1.61E-126 | -0.2871 | 0.255 | 0.515 | 3.55E-122 |
| MPZL3 | 6.08E-158 | -0.28718 | 0.046 | 0.293 | 1.34E-153 |
| PPP1R16B | 8.23E-157 | -0.28755 | 0.137 | 0.431 | 1.81E-152 |
| TRA2A | 1.35E-179 | -0.28758 | 0.052 | 0.33 | 2.98E-175 |
| UBE2V2 | 1.47E-194 | -0.28788 | 0.052 | 0.344 | 3.24E-190 |
| DAXX | 2.53E-189 | -0.28794 | 0.057 | 0.35 | 5.56E-185 |
| KAT6B | 4.16E-161 | -0.28796 | 0.037 | 0.279 | 9.17E-157 |
| SURF4 | 2.66E-192 | -0.28801 | 0.087 | 0.412 | 5.86E-188 |
| SUPT20H | 2.13E-191 | -0.28805 | 0.059 | 0.354 | 4.69E-187 |
| SERINC1 | 7.61E-172 | -0.28811 | 0.179 | 0.506 | 1.68E-167 |
| IRF2 | 4.16E-196 | -0.28821 | 0.088 | 0.417 | 9.16E-192 |
| CARD16 | 1.19E-155 | -0.28821 | 0.195 | 0.511 | 2.62E-151 |
| MTPN | 4.96E-194 | -0.2883 | 0.082 | 0.404 | 1.09E-189 |
| NOP53 | 1.55E-97 | -0.28842 | 0.991 | 0.837 | 3.42E-93 |
| CCNY | 5.62E-176 | -0.28848 | 0.045 | 0.311 | 1.24E-171 |
| RPL14 | 4.20E-198 | -0.28874 | 1 | 1 | 9.24E-194 |
| GSDMD | 4.44E-196 | -0.28876 | 0.062 | 0.367 | 9.77E-192 |
| GOLGB1 | 1.03E-181 | -0.28883 | 0.098 | 0.421 | 2.27E-177 |
| RPAIN | 5.21E-202 | -0.28895 | 0.091 | 0.43 | 1.15E-197 |
| SMYD3 | 7.43E-164 | -0.28896 | 0.065 | 0.336 | 1.64E-159 |
| YTHDC1 | 6.14E-204 | -0.28905 | 0.094 | 0.438 | 1.35E-199 |
| CPNE7 | 1.10E-138 | -0.28905 | 0.026 | 0.232 | 2.41E-134 |
| BBC3 | 3.48E-142 | -0.28934 | 0.076 | 0.331 | 7.67E-138 |
| PMPCB | 1.73E-199 | -0.28936 | 0.085 | 0.416 | 3.80E-195 |
| LAMTOR3 | 2.01E-188 | -0.28938 | 0.05 | 0.334 | 4.43E-184 |
| DERL2 | 5.89E-196 | -0.28962 | 0.051 | 0.344 | 1.30E-191 |
| NAA20 | 4.23E-203 | -0.28969 | 0.066 | 0.382 | 9.31E-199 |
| GET3 | 7.12E-206 | -0.28973 | 0.085 | 0.422 | 1.57E-201 |
| PIK3R1 | 1.56E-183 | -0.28974 | 0.121 | 0.451 | 3.42E-179 |
| ESYT1 | 7.57E-174 | -0.29015 | 0.053 | 0.325 | 1.67E-169 |
| GLUD1 | 5.04E-183 | -0.29018 | 0.065 | 0.358 | 1.11E-178 |
| TMED3 | 1.85E-180 | -0.29034 | 0.041 | 0.309 | 4.06E-176 |
| OLA1 | 7.82E-193 | -0.29046 | 0.096 | 0.422 | 1.72E-188 |
| PIM1 | 3.62E-169 | -0.29046 | 0.073 | 0.359 | 7.97E-165 |
| PDLIM2 | 8.53E-168 | -0.29062 | 0.024 | 0.261 | 1.88E-163 |
| FGD3 | 9.29E-203 | -0.29069 | 0.105 | 0.458 | 2.05E-198 |
| TSSC4 | 2.23E-196 | -0.29072 | 0.047 | 0.335 | 4.91E-192 |
| TRAPPC2L | 1.20E-202 | -0.29085 | 0.078 | 0.404 | 2.64E-198 |
| KDELR2 | 8.44E-195 | -0.29087 | 0.064 | 0.369 | 1.86E-190 |
| SSR1 | 5.86E-203 | -0.29091 | 0.08 | 0.41 | 1.29E-198 |
| KDM5A | 3.22E-184 | -0.29092 | 0.064 | 0.358 | 7.09E-180 |
| CYHR1 | 4.53E-184 | -0.29107 | 0.046 | 0.321 | 9.97E-180 |
| SRP19 | 8.28E-209 | -0.29116 | 0.095 | 0.446 | 1.82E-204 |
| RBM26 | 6.21E-179 | -0.29128 | 0.064 | 0.351 | 1.37E-174 |
| DCP2 | 1.68E-184 | -0.29153 | 0.054 | 0.339 | 3.70E-180 |
| P2RY10 | 5.75E-163 | -0.29162 | 0.062 | 0.328 | 1.27E-158 |
| HIST1H2AC | 2.14E-161 | -0.29164 | 0.053 | 0.311 | 4.72E-157 |
| CNPPD1 | 1.95E-211 | -0.29175 | 0.088 | 0.435 | 4.30E-207 |
| DCAF8 | 3.76E-180 | -0.29188 | 0.044 | 0.314 | 8.27E-176 |
| LSM10 | 8.64E-214 | -0.29189 | 0.077 | 0.413 | 1.90E-209 |
| SAMD9 | 1.55E-175 | -0.29203 | 0.037 | 0.296 | 3.42E-171 |
| SNX5 | 6.39E-197 | -0.29218 | 0.08 | 0.403 | 1.41E-192 |
| LPCAT4 | 3.62E-186 | -0.29223 | 0.066 | 0.364 | 7.97E-182 |
| AP1G2 | 2.44E-181 | -0.29228 | 0.064 | 0.355 | 5.37E-177 |
| CTCF | 7.99E-181 | -0.2923 | 0.065 | 0.356 | 1.76E-176 |
| ACTR6 | 2.52E-194 | -0.29247 | 0.04 | 0.321 | 5.55E-190 |
| OCIAD2 | 3.31E-94 | -0.29252 | 0.997 | 0.924 | 7.30E-90 |
| LSR | 3.57E-141 | -0.2927 | 0.018 | 0.22 | 7.87E-137 |
| SEC31A | 3.64E-188 | -0.29298 | 0.074 | 0.382 | 8.02E-184 |
| ARAP2 | 1.37E-164 | -0.29304 | 0.045 | 0.299 | 3.01E-160 |
| YME1L1 | 6.98E-186 | -0.29317 | 0.058 | 0.348 | 1.54E-181 |
| ECHS1 | 7.92E-212 | -0.29328 | 0.093 | 0.444 | 1.74E-207 |
| CD96 | 6.17E-81 | -0.29333 | 0.999 | 0.965 | 1.36E-76 |
| USP47 | 1.12E-192 | -0.29363 | 0.078 | 0.394 | 2.46E-188 |
| RSRC2 | 3.13E-200 | -0.29378 | 0.09 | 0.424 | 6.90E-196 |
| PITHD1 | 6.08E-177 | -0.2939 | 0.067 | 0.356 | 1.34E-172 |
| LTBP4 | 4.84E-186 | -0.29444 | 0.046 | 0.324 | 1.07E-181 |
| HELZ | 1.73E-174 | -0.29453 | 0.055 | 0.33 | 3.81E-170 |
| C1orf162 | 2.28E-127 | -0.29464 | 0.021 | 0.209 | 5.03E-123 |
| OGFR | 9.76E-195 | -0.29482 | 0.05 | 0.342 | 2.15E-190 |
| TXNIP | 5.67E-184 | -0.29492 | 0.091 | 0.41 | 1.25E-179 |
| KDM7A | 3.08E-168 | -0.29505 | 0.036 | 0.285 | 6.79E-164 |
| ITM2B | 1.70E-136 | -0.29531 | 1 | 0.992 | 3.74E-132 |
| ATP6AP1 | 1.10E-199 | -0.29537 | 0.051 | 0.349 | 2.43E-195 |
| PTPN2 | 1.99E-199 | -0.2955 | 0.077 | 0.399 | 4.39E-195 |
| MGMT | 5.25E-199 | -0.29558 | 0.066 | 0.378 | 1.15E-194 |
| SLC44A2 | 6.74E-183 | -0.29572 | 0.046 | 0.321 | 1.48E-178 |
| YTHDF2 | 1.96E-204 | -0.29574 | 0.084 | 0.417 | 4.32E-200 |
| REEP3 | 7.52E-173 | -0.29576 | 0.052 | 0.321 | 1.66E-168 |
| ERGIC2 | 2.60E-191 | -0.29583 | 0.051 | 0.34 | 5.72E-187 |
| EDEM3 | 7.33E-166 | -0.29589 | 0.031 | 0.272 | 1.61E-161 |
| MLLT10 | 2.78E-190 | -0.2959 | 0.058 | 0.352 | 6.11E-186 |
| ATP6V1G1 | 6.07E-144 | -0.29592 | 0.996 | 0.91 | 1.34E-139 |
| UBTF | 1.68E-191 | -0.29603 | 0.103 | 0.439 | 3.70E-187 |
| COMMD7 | 4.81E-159 | -0.29613 | 0.172 | 0.471 | 1.06E-154 |
| SMIM7 | 1.18E-197 | -0.29631 | 0.062 | 0.368 | 2.60E-193 |
| ISCA2 | 4.49E-212 | -0.29635 | 0.062 | 0.384 | 9.88E-208 |
| SUCLG2 | 1.08E-178 | -0.29645 | 0.052 | 0.327 | 2.38E-174 |
| BTN3A2 | 1.08E-187 | -0.29648 | 0.052 | 0.339 | 2.37E-183 |
| PSMB4 | 3.16E-203 | -0.29678 | 0.085 | 0.418 | 6.96E-199 |
| GPATCH8 | 7.76E-191 | -0.29693 | 0.071 | 0.378 | 1.71E-186 |
| UQCRB | 2.52E-146 | -0.29701 | 0.998 | 0.96 | 5.55E-142 |
| WAC | 1.06E-194 | -0.29714 | 0.079 | 0.399 | 2.34E-190 |
| LENG8 | 4.37E-177 | -0.29717 | 0.033 | 0.288 | 9.62E-173 |
| DENND4C | 9.71E-190 | -0.29724 | 0.071 | 0.376 | 2.14E-185 |
| COA5 | 7.38E-186 | -0.2973 | 0.046 | 0.324 | 1.62E-181 |
| MHENCR | 3.64E-167 | -0.29743 | 0.032 | 0.276 | 8.01E-163 |
| COP1 | 3.78E-180 | -0.29746 | 0.052 | 0.328 | 8.32E-176 |
| REV3L | 2.41E-171 | -0.29774 | 0.043 | 0.302 | 5.30E-167 |
| MED13L | 3.36E-176 | -0.29795 | 0.057 | 0.335 | 7.40E-172 |
| PCGF5 | 1.00E-196 | -0.29805 | 0.067 | 0.376 | 2.20E-192 |
| NDUFS3 | 2.64E-212 | -0.29812 | 0.08 | 0.419 | 5.81E-208 |
| MRPL28 | 7.17E-218 | -0.29822 | 0.076 | 0.418 | 1.58E-213 |
| PCMTD1 | 4.85E-189 | -0.29822 | 0.055 | 0.345 | 1.07E-184 |
| DNAJC19 | 1.85E-217 | -0.29823 | 0.088 | 0.441 | 4.07E-213 |
| EVA1B | 3.07E-127 | -0.29845 | 0.039 | 0.243 | 6.75E-123 |
| YPEL3 | 1.82E-117 | -0.29876 | 0.999 | 0.969 | 4.01E-113 |
| SS18 | 6.02E-189 | -0.29883 | 0.064 | 0.363 | 1.33E-184 |
| KDSR | 4.47E-181 | -0.29883 | 0.058 | 0.342 | 9.85E-177 |
| BAZ1A | 2.75E-184 | -0.29886 | 0.056 | 0.342 | 6.06E-180 |
| RPS2 | 1.26E-131 | -0.29904 | 1 | 1 | 2.78E-127 |
| NSD1 | 3.30E-187 | -0.29904 | 0.061 | 0.355 | 7.26E-183 |
| PHF14 | 9.42E-191 | -0.29931 | 0.057 | 0.35 | 2.07E-186 |
| CDK13 | 1.41E-185 | -0.29941 | 0.063 | 0.357 | 3.10E-181 |
| NAXE | 6.75E-216 | -0.29961 | 0.081 | 0.424 | 1.49E-211 |
| HERPUD2 | 7.31E-179 | -0.29965 | 0.045 | 0.313 | 1.61E-174 |
| ATP6V0D1 | 2.70E-215 | -0.29965 | 0.075 | 0.412 | 5.95E-211 |
| GLG1 | 9.72E-185 | -0.29966 | 0.064 | 0.359 | 2.14E-180 |
| SMARCC2 | 2.56E-198 | -0.29969 | 0.086 | 0.415 | 5.64E-194 |
| SMAD4 | 2.66E-187 | -0.29979 | 0.032 | 0.297 | 5.85E-183 |
| LRRC8D | 5.97E-165 | -0.2999 | 0.033 | 0.276 | 1.31E-160 |
| HBS1L | 9.65E-179 | -0.29994 | 0.072 | 0.364 | 2.12E-174 |
| CARD8 | 3.67E-189 | -0.29996 | 0.055 | 0.345 | 8.07E-185 |
| ACYP2 | 3.66E-185 | -0.30007 | 0.083 | 0.396 | 8.06E-181 |
| PCSK1N | 1.09E-109 | -0.30027 | 0.041 | 0.225 | 2.40E-105 |
| NAE1 | 1.65E-182 | -0.30028 | 0.054 | 0.336 | 3.63E-178 |
| EMC7 | 5.44E-237 | -0.3006 | 0.087 | 0.458 | 1.20E-232 |
| MRPL43 | 3.24E-190 | -0.30063 | 0.16 | 0.494 | 7.13E-186 |
| COA3 | 2.41E-224 | -0.30072 | 0.083 | 0.436 | 5.31E-220 |
| PHF11 | 2.66E-199 | -0.30083 | 0.087 | 0.418 | 5.86E-195 |
| SPAG7 | 3.94E-212 | -0.30086 | 0.096 | 0.449 | 8.67E-208 |
| RFTN1 | 1.63E-197 | -0.30089 | 0.079 | 0.401 | 3.59E-193 |
| AC022706.1 | 4.02E-171 | -0.30094 | 0.062 | 0.34 | 8.86E-167 |
| SNX1 | 3.13E-202 | -0.30099 | 0.064 | 0.377 | 6.89E-198 |
| CAMLG | 1.03E-204 | -0.30104 | 0.087 | 0.424 | 2.27E-200 |
| TIMM17B | 4.15E-218 | -0.30106 | 0.093 | 0.449 | 9.14E-214 |
| NDUFA5 | 2.72E-216 | -0.30107 | 0.082 | 0.426 | 5.98E-212 |
| CDC26 | 4.82E-199 | -0.30112 | 0.072 | 0.388 | 1.06E-194 |
| WBP1 | 4.18E-204 | -0.30121 | 0.076 | 0.403 | 9.21E-200 |
| SPG21 | 9.45E-202 | -0.30122 | 0.075 | 0.395 | 2.08E-197 |
| MAPRE2 | 4.20E-182 | -0.30128 | 0.033 | 0.294 | 9.24E-178 |
| MEA1 | 8.43E-211 | -0.30136 | 0.101 | 0.456 | 1.86E-206 |
| RASSF7 | 1.52E-198 | -0.30159 | 0.052 | 0.348 | 3.35E-194 |
| LINC00623 | 4.92E-211 | -0.30177 | 0.111 | 0.478 | 1.08E-206 |
| TMEM165 | 1.43E-202 | -0.30196 | 0.078 | 0.404 | 3.15E-198 |
| TOMM22 | 4.02E-194 | -0.30197 | 0.138 | 0.478 | 8.84E-190 |
| SMARCE1 | 4.42E-210 | -0.3022 | 0.095 | 0.445 | 9.73E-206 |
| SIK3 | 1.48E-182 | -0.30222 | 0.071 | 0.37 | 3.27E-178 |
| STK38 | 8.22E-195 | -0.30241 | 0.063 | 0.368 | 1.81E-190 |
| SRSF1 | 4.66E-190 | -0.30252 | 0.046 | 0.328 | 1.03E-185 |
| RAB18 | 6.12E-211 | -0.30268 | 0.084 | 0.425 | 1.35E-206 |
| ADAM10 | 5.10E-182 | -0.30275 | 0.061 | 0.349 | 1.12E-177 |
| FLYWCH2 | 1.22E-205 | -0.30279 | 0.058 | 0.368 | 2.69E-201 |
| CSNK1G2 | 7.30E-193 | -0.30281 | 0.052 | 0.343 | 1.61E-188 |
| ZNF800 | 7.77E-191 | -0.30283 | 0.051 | 0.339 | 1.71E-186 |
| PSENEN | 7.97E-204 | -0.30291 | 0.063 | 0.378 | 1.76E-199 |
| TUT4 | 1.51E-204 | -0.30291 | 0.115 | 0.477 | 3.32E-200 |
| TAF9 | 8.79E-194 | -0.30337 | 0.063 | 0.365 | 1.94E-189 |
| AFF1 | 5.00E-181 | -0.30352 | 0.041 | 0.309 | 1.10E-176 |
| SELENOM | 5.12E-170 | -0.30355 | 0.052 | 0.318 | 1.13E-165 |
| STOML2 | 3.71E-215 | -0.30377 | 0.099 | 0.447 | 8.17E-211 |
| NDUFS4 | 6.21E-202 | -0.30379 | 0.072 | 0.392 | 1.37E-197 |
| PAN3 | 1.44E-180 | -0.3038 | 0.053 | 0.331 | 3.17E-176 |
| GABPB1-AS1 | 3.37E-174 | -0.30387 | 0.03 | 0.28 | 7.43E-170 |
| RNF166 | 2.40E-198 | -0.30392 | 0.049 | 0.343 | 5.29E-194 |
| SYNE1 | 1.79E-159 | -0.30439 | 0.155 | 0.451 | 3.95E-155 |
| CEMIP2 | 9.84E-169 | -0.30455 | 0.054 | 0.321 | 2.17E-164 |
| PDCD2 | 7.63E-217 | -0.3046 | 0.078 | 0.42 | 1.68E-212 |
| PRRC2B | 3.04E-161 | -0.3048 | 0.137 | 0.433 | 6.68E-157 |
| SETD2 | 4.17E-198 | -0.30481 | 0.08 | 0.404 | 9.18E-194 |
| USP16 | 2.60E-186 | -0.30517 | 0.058 | 0.348 | 5.73E-182 |
| PSMD11 | 2.33E-211 | -0.30528 | 0.077 | 0.41 | 5.13E-207 |
| RGS1 | 6.10E-136 | -0.30538 | 0.081 | 0.331 | 1.34E-131 |
| STAM | 3.33E-163 | -0.3055 | 0.034 | 0.275 | 7.32E-159 |
| TCF12 | 2.99E-182 | -0.30587 | 0.064 | 0.355 | 6.59E-178 |
| LINC00649 | 3.62E-189 | -0.30595 | 0.061 | 0.356 | 7.97E-185 |
| ZNHIT3 | 7.91E-205 | -0.30596 | 0.07 | 0.391 | 1.74E-200 |
| DHX36 | 1.48E-187 | -0.30629 | 0.07 | 0.371 | 3.26E-183 |
| PSMC2 | 2.50E-204 | -0.30656 | 0.068 | 0.386 | 5.51E-200 |
| CCDC91 | 1.90E-190 | -0.30679 | 0.055 | 0.347 | 4.19E-186 |
| USP33 | 2.16E-190 | -0.30682 | 0.058 | 0.351 | 4.76E-186 |
| TSR3 | 9.97E-218 | -0.30685 | 0.072 | 0.407 | 2.20E-213 |
| RNF13 | 3.44E-191 | -0.30686 | 0.046 | 0.329 | 7.57E-187 |
| DPM3 | 5.58E-221 | -0.30687 | 0.127 | 0.519 | 1.23E-216 |
| UNC50 | 3.03E-205 | -0.30704 | 0.063 | 0.377 | 6.68E-201 |
| REXO2 | 4.32E-202 | -0.30707 | 0.078 | 0.403 | 9.50E-198 |
| RPS8 | 1.58E-199 | -0.30709 | 1 | 1 | 3.48E-195 |
| FAM172A | 1.95E-192 | -0.30713 | 0.062 | 0.361 | 4.30E-188 |
| RAB28 | 8.75E-184 | -0.30714 | 0.041 | 0.312 | 1.93E-179 |
| FAM214A | 1.99E-178 | -0.30715 | 0.048 | 0.319 | 4.37E-174 |
| ERP44 | 9.23E-206 | -0.30724 | 0.066 | 0.385 | 2.03E-201 |
| CREB1 | 2.38E-196 | -0.30742 | 0.063 | 0.368 | 5.24E-192 |
| CLIC3 | 1.33E-08 | -0.30749 | 0.467 | 0.463 | 0.000293 |
| HMG20B | 5.93E-197 | -0.3076 | 0.06 | 0.362 | 1.31E-192 |
| NUTM2B-AS1 | 2.23E-198 | -0.30765 | 0.08 | 0.403 | 4.91E-194 |
| MRPL14 | 1.16E-212 | -0.30793 | 0.081 | 0.419 | 2.55E-208 |
| SMURF2 | 7.78E-173 | -0.30811 | 0.054 | 0.324 | 1.71E-168 |
| ASXL1 | 2.15E-183 | -0.30823 | 0.116 | 0.44 | 4.74E-179 |
| DOCK9 | 8.24E-170 | -0.30825 | 0.028 | 0.269 | 1.81E-165 |
| RHEB | 1.25E-197 | -0.30829 | 0.1 | 0.441 | 2.74E-193 |
| PVT1 | 3.70E-186 | -0.30831 | 0.102 | 0.431 | 8.15E-182 |
| USP9Y | 8.31E-185 | -0.30838 | 0.051 | 0.331 | 1.83E-180 |
| CCR4 | 1.69E-131 | -0.30847 | 0.065 | 0.296 | 3.72E-127 |
| BTF3 | 1.20E-202 | -0.30851 | 1 | 0.999 | 2.64E-198 |
| ARID4A | 3.85E-187 | -0.30862 | 0.055 | 0.343 | 8.49E-183 |
| GMPR2 | 1.73E-188 | -0.3088 | 0.042 | 0.318 | 3.82E-184 |
| RPS9 | 4.86E-223 | -0.30929 | 1 | 1 | 1.07E-218 |
| PKIA | 3.44E-168 | -0.30931 | 0.069 | 0.348 | 7.58E-164 |
| NAPRT | 1.07E-186 | -0.30946 | 0.039 | 0.31 | 2.35E-182 |
| ANKHD1 | 5.64E-198 | -0.30966 | 0.075 | 0.393 | 1.24E-193 |
| SVBP | 7.78E-203 | -0.30986 | 0.063 | 0.377 | 1.71E-198 |
| SLC2A3 | 4.67E-202 | -0.30995 | 0.074 | 0.396 | 1.03E-197 |
| INPP5F | 7.84E-166 | -0.31005 | 0.048 | 0.305 | 1.73E-161 |
| PPP4R3B | 1.08E-190 | -0.31007 | 0.067 | 0.37 | 2.37E-186 |
| SDF2 | 6.40E-204 | -0.31008 | 0.058 | 0.366 | 1.41E-199 |
| IDH3G | 3.28E-222 | -0.31012 | 0.074 | 0.416 | 7.21E-218 |
| ARHGAP4 | 2.38E-194 | -0.31092 | 0.052 | 0.343 | 5.25E-190 |
| TSPOAP1-AS1 | 1.03E-178 | -0.31095 | 0.058 | 0.339 | 2.26E-174 |
| AC006369.1 | 7.49E-180 | -0.31109 | 0.073 | 0.37 | 1.65E-175 |
| TMED9 | 6.44E-194 | -0.31121 | 0.146 | 0.484 | 1.42E-189 |
| TMEM9B | 4.72E-199 | -0.31127 | 0.052 | 0.349 | 1.04E-194 |
| MRPL54 | 1.77E-200 | -0.3114 | 0.063 | 0.373 | 3.89E-196 |
| COPS5 | 4.85E-214 | -0.31156 | 0.071 | 0.403 | 1.07E-209 |
| M6PR | 5.01E-213 | -0.31165 | 0.063 | 0.386 | 1.10E-208 |
| TRADD | 1.86E-215 | -0.31192 | 0.073 | 0.408 | 4.08E-211 |
| ZNF644 | 6.05E-194 | -0.31201 | 0.066 | 0.37 | 1.33E-189 |
| TMEM259 | 1.16E-207 | -0.31208 | 0.065 | 0.386 | 2.56E-203 |
| TGIF1 | 2.57E-179 | -0.31216 | 0.044 | 0.314 | 5.65E-175 |
| NEK1 | 1.88E-178 | -0.31255 | 0.076 | 0.375 | 4.14E-174 |
| CLTC | 2.79E-206 | -0.31258 | 0.089 | 0.428 | 6.14E-202 |
| TOX4 | 2.02E-214 | -0.31261 | 0.059 | 0.38 | 4.45E-210 |
| ACAA1 | 2.04E-200 | -0.31284 | 0.055 | 0.358 | 4.48E-196 |
| CPNE3 | 1.50E-213 | -0.31309 | 0.083 | 0.426 | 3.30E-209 |
| AL136456.1 | 4.30E-92 | -0.31342 | 0.067 | 0.248 | 9.48E-88 |
| ITGB2 | 3.74E-183 | -0.31353 | 0.038 | 0.305 | 8.23E-179 |
| MFF | 1.31E-168 | -0.31364 | 0.188 | 0.499 | 2.89E-164 |
| ZCCHC7 | 5.24E-192 | -0.31365 | 0.056 | 0.35 | 1.15E-187 |
| SRPRA | 2.28E-215 | -0.31374 | 0.068 | 0.399 | 5.03E-211 |
| LRP10 | 1.76E-210 | -0.31381 | 0.076 | 0.408 | 3.88E-206 |
| SLC66A3 | 5.10E-200 | -0.31388 | 0.049 | 0.345 | 1.12E-195 |
| HBP1 | 9.07E-204 | -0.31421 | 0.047 | 0.345 | 2.00E-199 |
| CRBN | 3.64E-204 | -0.31428 | 0.055 | 0.361 | 8.01E-200 |
| MPST | 2.79E-196 | -0.31438 | 0.081 | 0.402 | 6.15E-192 |
| WWP1 | 1.55E-188 | -0.31441 | 0.047 | 0.327 | 3.42E-184 |
| N4BP1 | 1.90E-203 | -0.3145 | 0.081 | 0.411 | 4.18E-199 |
| GOLGA4 | 1.33E-186 | -0.31451 | 0.065 | 0.361 | 2.92E-182 |
| PPP1R15A | 1.85E-202 | -0.31462 | 0.09 | 0.428 | 4.07E-198 |
| HPS1 | 3.40E-211 | -0.31503 | 0.083 | 0.421 | 7.48E-207 |
| ADD1 | 6.69E-203 | -0.31511 | 0.095 | 0.43 | 1.47E-198 |
| PRKCQ | 1.76E-139 | -0.31513 | 0.193 | 0.464 | 3.87E-135 |
| PFDN4 | 8.22E-207 | -0.31532 | 0.085 | 0.421 | 1.81E-202 |
| PREX1 | 5.32E-196 | -0.31537 | 0.065 | 0.373 | 1.17E-191 |
| MED28 | 1.14E-216 | -0.31559 | 0.067 | 0.398 | 2.52E-212 |
| ARFGEF1 | 1.88E-197 | -0.31566 | 0.059 | 0.361 | 4.15E-193 |
| BNIP2 | 2.43E-210 | -0.31567 | 0.063 | 0.384 | 5.34E-206 |
| GIT2 | 9.38E-210 | -0.31572 | 0.07 | 0.395 | 2.06E-205 |
| AC243960.1 | 1.14E-206 | -0.31578 | 0.064 | 0.382 | 2.51E-202 |
| ZEB1 | 2.26E-186 | -0.31589 | 0.073 | 0.376 | 4.99E-182 |
| NUDT22 | 2.71E-215 | -0.31604 | 0.075 | 0.411 | 5.98E-211 |
| CPNE1 | 1.18E-199 | -0.31608 | 0.125 | 0.468 | 2.59E-195 |
| NFATC3 | 8.36E-186 | -0.31634 | 0.056 | 0.343 | 1.84E-181 |
| ATP11B | 1.56E-192 | -0.31649 | 0.048 | 0.334 | 3.42E-188 |
| UBE2H | 1.84E-195 | -0.3166 | 0.061 | 0.363 | 4.04E-191 |
| TBL1XR1 | 2.42E-200 | -0.31661 | 0.076 | 0.398 | 5.32E-196 |
| COMMD8 | 3.04E-209 | -0.31679 | 0.085 | 0.426 | 6.69E-205 |
| CERK | 1.35E-187 | -0.31682 | 0.058 | 0.348 | 2.98E-183 |
| EEF1G | 1.89E-184 | -0.31712 | 1 | 0.999 | 4.16E-180 |
| UBE2E1 | 2.84E-203 | -0.31713 | 0.052 | 0.355 | 6.25E-199 |
| FBXO34 | 3.60E-180 | -0.31725 | 0.047 | 0.318 | 7.92E-176 |
| S100PBP | 1.32E-188 | -0.31728 | 0.075 | 0.383 | 2.90E-184 |
| C11orf68 | 1.27E-203 | -0.31729 | 0.041 | 0.334 | 2.79E-199 |
| TMEM238 | 2.25E-169 | -0.31791 | 0.156 | 0.466 | 4.96E-165 |
| MT-CYB | 2.88E-111 | -0.31811 | 1 | 0.997 | 6.34E-107 |
| SETX | 1.28E-206 | -0.31845 | 0.069 | 0.391 | 2.83E-202 |
| ARL6IP6 | 1.79E-201 | -0.31849 | 0.074 | 0.395 | 3.94E-197 |
| CCDC12 | 1.54E-210 | -0.31911 | 0.072 | 0.4 | 3.40E-206 |
| TMF1 | 5.49E-196 | -0.31916 | 0.049 | 0.34 | 1.21E-191 |
| ZFP36L1 | 3.80E-46 | -0.31924 | 0.989 | 0.784 | 8.36E-42 |
| ATP2B1 | 6.29E-182 | -0.31932 | 0.049 | 0.325 | 1.38E-177 |
| CRLF3 | 2.53E-194 | -0.31933 | 0.04 | 0.322 | 5.58E-190 |
| GCHFR | 8.28E-184 | -0.31948 | 0.059 | 0.346 | 1.82E-179 |
| SIGIRR | 5.85E-212 | -0.31962 | 0.059 | 0.378 | 1.29E-207 |
| TNFRSF1B | 6.08E-149 | -0.31976 | 0.134 | 0.424 | 1.34E-144 |
| MRFAP1 | 2.53E-217 | -0.31989 | 0.069 | 0.402 | 5.58E-213 |
| ORAI1 | 1.29E-179 | -0.32007 | 0.17 | 0.494 | 2.84E-175 |
| PLD3 | 1.84E-217 | -0.32012 | 0.072 | 0.408 | 4.05E-213 |
| PIH1D1 | 3.69E-208 | -0.32022 | 0.055 | 0.365 | 8.13E-204 |
| SRSF8 | 1.48E-205 | -0.32046 | 0.055 | 0.362 | 3.26E-201 |
| TBC1D10A | 1.50E-202 | -0.32048 | 0.038 | 0.325 | 3.30E-198 |
| PUF60 | 1.58E-226 | -0.32064 | 0.119 | 0.487 | 3.48E-222 |
| PHYKPL | 3.37E-213 | -0.32068 | 0.059 | 0.378 | 7.42E-209 |
| MRPS12 | 1.82E-220 | -0.3208 | 0.062 | 0.391 | 4.02E-216 |
| VASP | 8.85E-207 | -0.32082 | 0.063 | 0.38 | 1.95E-202 |
| ARF4 | 1.64E-231 | -0.32085 | 0.128 | 0.516 | 3.62E-227 |
| RBM4 | 7.04E-218 | -0.32089 | 0.085 | 0.433 | 1.55E-213 |
| RAB8B | 5.67E-205 | -0.32092 | 0.083 | 0.417 | 1.25E-200 |
| DENND1B | 5.90E-183 | -0.321 | 0.055 | 0.338 | 1.30E-178 |
| FOXO1 | 5.01E-188 | -0.32103 | 0.046 | 0.326 | 1.10E-183 |
| CCDC28A | 7.40E-216 | -0.32135 | 0.059 | 0.381 | 1.63E-211 |
| THOC2 | 1.52E-204 | -0.32136 | 0.052 | 0.355 | 3.35E-200 |
| KRCC1 | 1.10E-220 | -0.32144 | 0.084 | 0.435 | 2.43E-216 |
| S1PR1 | 3.92E-164 | -0.32169 | 0.035 | 0.279 | 8.64E-160 |
| PUM2 | 2.79E-201 | -0.32174 | 0.065 | 0.376 | 6.14E-197 |
| CHCHD10 | 2.00E-201 | -0.32194 | 0.079 | 0.404 | 4.40E-197 |
| URM1 | 1.94E-215 | -0.32202 | 0.064 | 0.391 | 4.27E-211 |
| FKBP5 | 2.00E-190 | -0.32208 | 0.057 | 0.351 | 4.40E-186 |
| POLR2B | 4.26E-221 | -0.32209 | 0.081 | 0.429 | 9.38E-217 |
| MAN1A2 | 4.47E-199 | -0.32215 | 0.056 | 0.357 | 9.84E-195 |
| LAMP1 | 1.35E-238 | -0.32226 | 0.093 | 0.469 | 2.98E-234 |
| UBR2 | 1.35E-204 | -0.32227 | 0.062 | 0.376 | 2.98E-200 |
| SNRNP40 | 3.44E-225 | -0.32263 | 0.078 | 0.426 | 7.58E-221 |
| SMIM29 | 2.21E-233 | -0.32269 | 0.086 | 0.453 | 4.87E-229 |
| TMEM245 | 1.17E-190 | -0.3229 | 0.052 | 0.339 | 2.57E-186 |
| GORASP2 | 1.65E-212 | -0.32292 | 0.057 | 0.374 | 3.64E-208 |
| MOSPD3 | 2.27E-215 | -0.32308 | 0.065 | 0.392 | 5.00E-211 |
| NLRP1 | 4.15E-186 | -0.32332 | 0.031 | 0.295 | 9.13E-182 |
| RACK1 | 8.93E-210 | -0.32333 | 1 | 1 | 1.97E-205 |
| IP6K2 | 1.92E-211 | -0.32336 | 0.063 | 0.383 | 4.24E-207 |
| CALHM2 | 3.73E-201 | -0.32343 | 0.063 | 0.374 | 8.20E-197 |
| SYNRG | 7.99E-161 | -0.32358 | 0.177 | 0.473 | 1.76E-156 |
| BLOC1S2 | 7.79E-221 | -0.32362 | 0.08 | 0.426 | 1.71E-216 |
| SYTL2 | 3.92E-138 | -0.32392 | 0.043 | 0.263 | 8.63E-134 |
| GLOD4 | 9.21E-208 | -0.32417 | 0.056 | 0.367 | 2.03E-203 |
| SNX2 | 1.63E-212 | -0.32419 | 0.062 | 0.382 | 3.59E-208 |
| RRAGA | 1.46E-218 | -0.32448 | 0.065 | 0.396 | 3.21E-214 |
| RASA2 | 1.94E-198 | -0.32456 | 0.07 | 0.383 | 4.27E-194 |
| R3HDM2 | 2.50E-215 | -0.32476 | 0.074 | 0.41 | 5.50E-211 |
| UBE2G1 | 4.38E-205 | -0.3249 | 0.054 | 0.36 | 9.65E-201 |
| VPS51 | 5.77E-209 | -0.32494 | 0.06 | 0.375 | 1.27E-204 |
| RICTOR | 5.09E-193 | -0.32508 | 0.06 | 0.359 | 1.12E-188 |
| CYB5R3 | 3.10E-219 | -0.3251 | 0.075 | 0.417 | 6.84E-215 |
| ABT1 | 3.71E-226 | -0.32512 | 0.061 | 0.395 | 8.18E-222 |
| ATF6B | 2.79E-215 | -0.32525 | 0.08 | 0.42 | 6.13E-211 |
| RB1CC1 | 2.57E-194 | -0.32528 | 0.051 | 0.342 | 5.65E-190 |
| NFAT5 | 3.77E-194 | -0.3259 | 0.061 | 0.363 | 8.31E-190 |
| SNRNP70 | 3.16E-224 | -0.32639 | 0.092 | 0.453 | 6.96E-220 |
| NEK7 | 1.71E-194 | -0.3264 | 0.065 | 0.37 | 3.77E-190 |
| ARL15 | 1.11E-185 | -0.32653 | 0.039 | 0.309 | 2.45E-181 |
| KLF12 | 5.00E-185 | -0.3266 | 0.042 | 0.316 | 1.10E-180 |
| WDR33 | 8.52E-220 | -0.32679 | 0.072 | 0.409 | 1.88E-215 |
| BCL7C | 2.94E-194 | -0.32695 | 0.146 | 0.482 | 6.47E-190 |
| ZNF148 | 9.57E-199 | -0.32704 | 0.055 | 0.354 | 2.11E-194 |
| B3GAT3 | 1.31E-216 | -0.32719 | 0.066 | 0.396 | 2.89E-212 |
| CCAR1 | 3.34E-196 | -0.32748 | 0.078 | 0.396 | 7.35E-192 |
| RNASEH2B | 1.12E-207 | -0.32762 | 0.077 | 0.407 | 2.46E-203 |
| KDM4C | 1.31E-190 | -0.32762 | 0.025 | 0.287 | 2.89E-186 |
| AC004687.1 | 6.35E-199 | -0.32777 | 0.089 | 0.423 | 1.40E-194 |
| GZMM | 2.56E-182 | -0.32783 | 0.055 | 0.338 | 5.63E-178 |
| GPR155 | 1.82E-171 | -0.32783 | 0.026 | 0.268 | 4.02E-167 |
| CUL3 | 3.39E-221 | -0.32801 | 0.065 | 0.399 | 7.47E-217 |
| SKIL | 2.96E-188 | -0.32808 | 0.061 | 0.354 | 6.53E-184 |
| DAZAP1 | 2.96E-214 | -0.32831 | 0.09 | 0.44 | 6.51E-210 |
| LRRN3 | 2.49E-84 | -0.32837 | 0.041 | 0.192 | 5.48E-80 |
| RPS25 | 1.57E-203 | -0.32864 | 1 | 1 | 3.45E-199 |
| IRF3 | 7.66E-215 | -0.32903 | 0.063 | 0.388 | 1.69E-210 |
| UFL1 | 1.05E-199 | -0.32931 | 0.051 | 0.348 | 2.31E-195 |
| SAT2 | 4.78E-222 | -0.32935 | 0.065 | 0.398 | 1.05E-217 |
| ZNF655 | 2.37E-208 | -0.32938 | 0.048 | 0.351 | 5.21E-204 |
| EXOSC1 | 1.05E-226 | -0.3295 | 0.063 | 0.4 | 2.32E-222 |
| PPWD1 | 6.94E-205 | -0.32976 | 0.041 | 0.333 | 1.53E-200 |
| TTC14 | 6.10E-198 | -0.32982 | 0.045 | 0.333 | 1.34E-193 |
| NAAA | 3.92E-155 | -0.33017 | 0.073 | 0.339 | 8.63E-151 |
| YIPF3 | 3.04E-235 | -0.33029 | 0.091 | 0.462 | 6.68E-231 |
| PSMC6 | 3.55E-229 | -0.33032 | 0.065 | 0.405 | 7.83E-225 |
| ARHGEF3 | 1.44E-184 | -0.33046 | 0.051 | 0.332 | 3.16E-180 |
| HERC4 | 3.96E-215 | -0.33047 | 0.075 | 0.411 | 8.72E-211 |
| ITPR2 | 4.78E-188 | -0.33059 | 0.091 | 0.412 | 1.05E-183 |
| WBP2 | 3.56E-234 | -0.33092 | 0.084 | 0.447 | 7.83E-230 |
| SENP6 | 1.34E-210 | -0.33104 | 0.082 | 0.421 | 2.95E-206 |
| PRKY | 9.26E-189 | -0.33118 | 0.038 | 0.311 | 2.04E-184 |
| LYPLA2 | 2.90E-217 | -0.33141 | 0.061 | 0.385 | 6.38E-213 |
| GALNT10 | 9.94E-189 | -0.33141 | 0.056 | 0.346 | 2.19E-184 |
| OFD1 | 6.11E-200 | -0.33155 | 0.038 | 0.322 | 1.34E-195 |
| RPLP1 | 1.02E-292 | -0.3316 | 1 | 1 | 2.25E-288 |
| COPB2 | 4.03E-214 | -0.3316 | 0.08 | 0.42 | 8.87E-210 |
| VPS36 | 5.06E-211 | -0.33166 | 0.067 | 0.391 | 1.11E-206 |
| EXOC4 | 3.38E-198 | -0.33173 | 0.055 | 0.355 | 7.45E-194 |
| ZNF217 | 9.29E-204 | -0.33177 | 0.053 | 0.357 | 2.05E-199 |
| NCF4 | 6.00E-183 | -0.3319 | 0.067 | 0.361 | 1.32E-178 |
| FRYL | 8.38E-219 | -0.33192 | 0.08 | 0.426 | 1.84E-214 |
| CAPN1 | 1.04E-225 | -0.33201 | 0.071 | 0.413 | 2.29E-221 |
| METTL5 | 1.67E-247 | -0.33246 | 0.086 | 0.466 | 3.67E-243 |
| NCK1 | 1.20E-220 | -0.33257 | 0.064 | 0.396 | 2.64E-216 |
| APPL2 | 2.26E-185 | -0.33288 | 0.031 | 0.293 | 4.97E-181 |
| DAP3 | 1.11E-228 | -0.33288 | 0.082 | 0.439 | 2.45E-224 |
| APEX1 | 3.00E-209 | -0.33295 | 0.078 | 0.411 | 6.60E-205 |
| KLHDC2 | 1.78E-216 | -0.33312 | 0.053 | 0.37 | 3.92E-212 |
| COMT | 8.31E-235 | -0.33322 | 0.08 | 0.439 | 1.83E-230 |
| PCSK7 | 2.76E-198 | -0.3334 | 0.035 | 0.315 | 6.07E-194 |
| NCKAP1L | 2.30E-216 | -0.33374 | 0.076 | 0.415 | 5.06E-212 |
| CHD1 | 3.98E-198 | -0.33385 | 0.052 | 0.346 | 8.76E-194 |
| SP3 | 3.90E-211 | -0.33386 | 0.059 | 0.376 | 8.58E-207 |
| FAU | 1.31E-245 | -0.33393 | 1 | 1 | 2.87E-241 |
| YIPF4 | 6.58E-231 | -0.33416 | 0.068 | 0.413 | 1.45E-226 |
| MRFAP1L1 | 4.99E-182 | -0.33432 | 0.201 | 0.532 | 1.10E-177 |
| SUMO3 | 4.82E-219 | -0.33435 | 0.1 | 0.462 | 1.06E-214 |
| NT5C3A | 7.09E-206 | -0.33444 | 0.041 | 0.334 | 1.56E-201 |
| SDHAF2 | 2.33E-249 | -0.33463 | 0.108 | 0.509 | 5.14E-245 |
| OGT | 1.32E-197 | -0.33466 | 0.057 | 0.358 | 2.90E-193 |
| SATB1 | 1.44E-191 | -0.33475 | 0.058 | 0.352 | 3.18E-187 |
| FTX | 1.46E-206 | -0.3349 | 0.072 | 0.397 | 3.22E-202 |
| LY9 | 6.88E-201 | -0.33505 | 0.04 | 0.329 | 1.51E-196 |
| RGS14 | 5.78E-207 | -0.33551 | 0.037 | 0.327 | 1.27E-202 |
| C1D | 3.25E-241 | -0.33575 | 0.067 | 0.42 | 7.15E-237 |
| SLC25A39 | 5.17E-222 | -0.33576 | 0.065 | 0.398 | 1.14E-217 |
| CNOT2 | 4.48E-219 | -0.33581 | 0.066 | 0.398 | 9.86E-215 |
| TIAL1 | 4.42E-232 | -0.33693 | 0.085 | 0.447 | 9.73E-228 |
| STXBP2 | 6.04E-243 | -0.33714 | 0.087 | 0.463 | 1.33E-238 |
| RPL35A | 3.94E-253 | -0.33714 | 1 | 1 | 8.68E-249 |
| TMEM60 | 4.97E-223 | -0.33723 | 0.052 | 0.374 | 1.09E-218 |
| LYAR | 2.97E-132 | -0.33742 | 0.057 | 0.282 | 6.53E-128 |
| ETV6 | 3.26E-186 | -0.3376 | 0.044 | 0.32 | 7.17E-182 |
| ECHDC1 | 3.04E-219 | -0.33767 | 0.051 | 0.367 | 6.69E-215 |
| UBA2 | 1.69E-233 | -0.33767 | 0.075 | 0.429 | 3.72E-229 |
| SHFL | 3.18E-238 | -0.33778 | 0.088 | 0.461 | 7.00E-234 |
| LRRC8C | 7.20E-215 | -0.33793 | 0.063 | 0.388 | 1.58E-210 |
| HIKESHI | 2.32E-234 | -0.33798 | 0.091 | 0.462 | 5.11E-230 |
| RGS19 | 3.52E-223 | -0.33808 | 0.084 | 0.438 | 7.76E-219 |
| PTDSS1 | 5.26E-222 | -0.33817 | 0.074 | 0.416 | 1.16E-217 |
| YPEL2 | 3.88E-203 | -0.33819 | 0.064 | 0.378 | 8.55E-199 |
| CDR2 | 1.57E-202 | -0.33821 | 0.056 | 0.36 | 3.46E-198 |
| CDC42SE1 | 2.12E-220 | -0.33825 | 0.083 | 0.432 | 4.67E-216 |
| RPL29 | 1.05E-251 | -0.33866 | 1 | 1 | 2.31E-247 |
| CCS | 2.11E-228 | -0.33932 | 0.057 | 0.39 | 4.64E-224 |
| MPLKIP | 2.47E-230 | -0.33935 | 0.063 | 0.402 | 5.43E-226 |
| CD58 | 8.51E-205 | -0.33956 | 0.124 | 0.471 | 1.87E-200 |
| STAT3 | 6.60E-222 | -0.33984 | 0.063 | 0.394 | 1.45E-217 |
| LEPROTL1 | 4.83E-122 | -0.34046 | 0.985 | 0.838 | 1.06E-117 |
| FLOT2 | 1.79E-207 | -0.34058 | 0.05 | 0.354 | 3.93E-203 |
| NEMF | 1.02E-210 | -0.34076 | 0.061 | 0.379 | 2.24E-206 |
| AC008105.3 | 1.05E-199 | -0.34091 | 0.073 | 0.391 | 2.32E-195 |
| SMAD2 | 1.03E-224 | -0.34127 | 0.065 | 0.402 | 2.26E-220 |
| NECAP2 | 2.35E-236 | -0.34127 | 0.077 | 0.437 | 5.16E-232 |
| PYM1 | 3.70E-218 | -0.3413 | 0.077 | 0.419 | 8.14E-214 |
| TRMT10C | 3.84E-207 | -0.34135 | 0.033 | 0.319 | 8.46E-203 |
| MIS18BP1 | 1.55E-198 | -0.34139 | 0.098 | 0.436 | 3.41E-194 |
| MORC3 | 1.65E-208 | -0.34161 | 0.04 | 0.336 | 3.63E-204 |
| SENP7 | 4.63E-206 | -0.34162 | 0.054 | 0.361 | 1.02E-201 |
| SFT2D1 | 1.88E-225 | -0.34177 | 0.077 | 0.426 | 4.15E-221 |
| CNPY3 | 3.27E-218 | -0.34201 | 0.058 | 0.381 | 7.20E-214 |
| UTY | 6.35E-198 | -0.34231 | 0.051 | 0.344 | 1.40E-193 |
| STX8 | 7.65E-231 | -0.34258 | 0.082 | 0.44 | 1.68E-226 |
| RANBP9 | 5.19E-221 | -0.34274 | 0.092 | 0.448 | 1.14E-216 |
| THYN1 | 1.12E-254 | -0.34297 | 0.09 | 0.479 | 2.47E-250 |
| ABI1 | 2.19E-220 | -0.34302 | 0.109 | 0.469 | 4.81E-216 |
| TNFSF13B | 1.77E-151 | -0.34316 | 0.068 | 0.325 | 3.89E-147 |
| GPR171 | 1.47E-180 | -0.34324 | 0.07 | 0.364 | 3.23E-176 |
| BTG2 | 6.41E-200 | -0.34325 | 0.053 | 0.352 | 1.41E-195 |
| USP34 | 1.13E-228 | -0.3434 | 0.095 | 0.462 | 2.48E-224 |
| AC092821.3 | 8.06E-222 | -0.34346 | 0.062 | 0.392 | 1.78E-217 |
| DNAJA2 | 3.44E-210 | -0.34348 | 0.143 | 0.497 | 7.56E-206 |
| AUP1 | 1.96E-226 | -0.34353 | 0.119 | 0.487 | 4.32E-222 |
| PRNP | 1.24E-212 | -0.34356 | 0.094 | 0.444 | 2.73E-208 |
| SMIM19 | 1.65E-227 | -0.34357 | 0.068 | 0.411 | 3.64E-223 |
| UBA3 | 3.80E-225 | -0.34358 | 0.066 | 0.404 | 8.36E-221 |
| SPATA13 | 1.92E-223 | -0.34364 | 0.07 | 0.41 | 4.22E-219 |
| BRWD1 | 1.26E-206 | -0.34376 | 0.048 | 0.349 | 2.77E-202 |
| SUCLG1 | 1.42E-235 | -0.34424 | 0.081 | 0.443 | 3.12E-231 |
| GIPC1 | 4.52E-226 | -0.34431 | 0.062 | 0.398 | 9.96E-222 |
| RPN1 | 2.84E-239 | -0.34433 | 0.08 | 0.447 | 6.25E-235 |
| GLRX5 | 6.46E-236 | -0.34448 | 0.09 | 0.46 | 1.42E-231 |
| FLI1 | 2.78E-206 | -0.34495 | 0.038 | 0.328 | 6.13E-202 |
| CENPC | 1.61E-222 | -0.34503 | 0.076 | 0.42 | 3.55E-218 |
| C18orf32 | 9.15E-254 | -0.34512 | 0.095 | 0.488 | 2.01E-249 |
| AASDHPPT | 1.84E-234 | -0.34555 | 0.063 | 0.407 | 4.06E-230 |
| CAMK2D | 1.42E-196 | -0.34608 | 0.064 | 0.369 | 3.14E-192 |
| ITFG1 | 2.05E-213 | -0.34622 | 0.063 | 0.385 | 4.52E-209 |
| BECN1 | 1.22E-220 | -0.34627 | 0.055 | 0.377 | 2.68E-216 |
| MRPL10 | 4.76E-225 | -0.34654 | 0.068 | 0.409 | 1.05E-220 |
| RBM42 | 1.17E-235 | -0.34709 | 0.082 | 0.445 | 2.57E-231 |
| EML3 | 3.10E-217 | -0.34724 | 0.055 | 0.374 | 6.84E-213 |
| PTBP3 | 6.22E-220 | -0.34742 | 0.079 | 0.424 | 1.37E-215 |
| ARID1B | 1.39E-210 | -0.34772 | 0.075 | 0.407 | 3.07E-206 |
| THAP11 | 4.11E-238 | -0.34804 | 0.053 | 0.39 | 9.04E-234 |
| PTPRJ | 2.47E-213 | -0.34805 | 0.077 | 0.413 | 5.44E-209 |
| EAPP | 1.18E-232 | -0.34818 | 0.12 | 0.501 | 2.60E-228 |
| COA1 | 9.47E-205 | -0.34856 | 0.058 | 0.367 | 2.09E-200 |
| GPBP1 | 1.52E-236 | -0.34862 | 0.107 | 0.495 | 3.34E-232 |
| MBNL2 | 1.74E-216 | -0.34866 | 0.087 | 0.436 | 3.83E-212 |
| TNFSF8 | 4.90E-161 | -0.34872 | 0.043 | 0.29 | 1.08E-156 |
| RAPGEF1 | 2.75E-226 | -0.34882 | 0.086 | 0.442 | 6.06E-222 |
| ODF2L | 2.38E-211 | -0.34931 | 0.063 | 0.384 | 5.23E-207 |
| TNFRSF1A | 2.95E-218 | -0.34998 | 0.048 | 0.361 | 6.50E-214 |
| NUDCD2 | 3.80E-234 | -0.34998 | 0.066 | 0.411 | 8.37E-230 |
| CMTM7 | 2.56E-212 | -0.35006 | 0.065 | 0.388 | 5.64E-208 |
| PPP1R11 | 3.92E-231 | -0.35108 | 0.065 | 0.408 | 8.63E-227 |
| REST | 5.22E-224 | -0.35123 | 0.081 | 0.433 | 1.15E-219 |
| RASSF5 | 4.47E-219 | -0.35128 | 0.052 | 0.369 | 9.83E-215 |
| EVI2A | 1.27E-216 | -0.35133 | 0.068 | 0.398 | 2.79E-212 |
| TMUB1 | 3.01E-245 | -0.35149 | 0.078 | 0.447 | 6.62E-241 |
| UBLCP1 | 4.67E-242 | -0.35214 | 0.093 | 0.473 | 1.03E-237 |
| TNIK | 1.94E-196 | -0.35225 | 0.058 | 0.359 | 4.27E-192 |
| PPHLN1 | 3.17E-229 | -0.35253 | 0.08 | 0.434 | 6.98E-225 |
| YIPF5 | 4.06E-235 | -0.35259 | 0.106 | 0.492 | 8.95E-231 |
| FAM117A | 6.51E-238 | -0.35303 | 0.091 | 0.467 | 1.43E-233 |
| PLCL1 | 1.13E-148 | -0.35309 | 0.029 | 0.247 | 2.48E-144 |
| ARRDC1 | 9.63E-240 | -0.35348 | 0.055 | 0.397 | 2.12E-235 |
| PERP | 4.62E-176 | -0.35372 | 0.064 | 0.346 | 1.02E-171 |
| ERBIN | 1.52E-214 | -0.35385 | 0.069 | 0.399 | 3.34E-210 |
| TBC1D4 | 3.16E-156 | -0.35395 | 0.035 | 0.269 | 6.96E-152 |
| NSMCE3 | 6.00E-231 | -0.35397 | 0.051 | 0.379 | 1.32E-226 |
| PDE4D | 4.04E-192 | -0.3541 | 0.054 | 0.345 | 8.90E-188 |
| CNOT6L | 2.81E-220 | -0.35417 | 0.061 | 0.387 | 6.18E-216 |
| RSF1 | 2.85E-230 | -0.35562 | 0.089 | 0.45 | 6.28E-226 |
| PNN | 3.19E-235 | -0.35574 | 0.081 | 0.443 | 7.02E-231 |
| UBAC2 | 6.36E-242 | -0.35579 | 0.067 | 0.423 | 1.40E-237 |
| ARL5A | 3.13E-232 | -0.35685 | 0.055 | 0.389 | 6.89E-228 |
| TIPARP | 2.18E-206 | -0.35706 | 0.071 | 0.393 | 4.80E-202 |
| KAT6A | 2.80E-213 | -0.35732 | 0.063 | 0.385 | 6.17E-209 |
| SYPL1 | 1.98E-218 | -0.35749 | 0.047 | 0.36 | 4.36E-214 |
| DCK | 2.11E-221 | -0.35751 | 0.049 | 0.366 | 4.64E-217 |
| BACH2 | 1.73E-166 | -0.35754 | 0.071 | 0.348 | 3.81E-162 |
| MPRIP | 1.35E-222 | -0.35762 | 0.075 | 0.42 | 2.98E-218 |
| ZFAND5 | 3.02E-223 | -0.35802 | 0.076 | 0.42 | 6.65E-219 |
| DNAJB1 | 1.97E-230 | -0.35806 | 0.076 | 0.428 | 4.34E-226 |
| CHKB | 1.38E-227 | -0.35862 | 0.049 | 0.372 | 3.04E-223 |
| RAB6A | 2.93E-225 | -0.35916 | 0.048 | 0.367 | 6.45E-221 |
| TBC1D5 | 1.50E-221 | -0.3599 | 0.087 | 0.441 | 3.31E-217 |
| TBC1D22A | 8.05E-230 | -0.36048 | 0.082 | 0.44 | 1.77E-225 |
| MGAT5 | 1.66E-184 | -0.36048 | 0.04 | 0.311 | 3.67E-180 |
| RPL18 | 0 | -0.36066 | 1 | 1 | 0 |
| TOP2B | 3.94E-228 | -0.36106 | 0.063 | 0.4 | 8.68E-224 |
| GRSF1 | 6.05E-238 | -0.36111 | 0.065 | 0.414 | 1.33E-233 |
| PICALM | 1.53E-225 | -0.36111 | 0.062 | 0.396 | 3.38E-221 |
| RPL9 | 5.10E-190 | -0.36136 | 1 | 0.999 | 1.12E-185 |
| IFITM1 | 3.85E-62 | -0.36142 | 0.99 | 0.794 | 8.47E-58 |
| SESN1 | 2.29E-183 | -0.36171 | 0.059 | 0.345 | 5.05E-179 |
| FUT8 | 1.89E-202 | -0.36214 | 0.073 | 0.394 | 4.15E-198 |
| TMEM134 | 1.96E-232 | -0.36226 | 0.056 | 0.392 | 4.33E-228 |
| DUSP16 | 4.44E-194 | -0.36242 | 0.053 | 0.345 | 9.78E-190 |
| CCDC115 | 3.74E-239 | -0.36284 | 0.059 | 0.404 | 8.24E-235 |
| ALOX5AP | 2.19E-15 | -0.36297 | 0.994 | 0.756 | 4.81E-11 |
| ABTB1 | 4.94E-234 | -0.36389 | 0.058 | 0.397 | 1.09E-229 |
| AKR1B1 | 2.83E-229 | -0.36407 | 0.056 | 0.388 | 6.24E-225 |
| PDE7A | 7.43E-173 | -0.36435 | 0.158 | 0.46 | 1.64E-168 |
| PPP6C | 5.40E-250 | -0.36455 | 0.064 | 0.424 | 1.19E-245 |
| MICU2 | 1.64E-226 | -0.36487 | 0.05 | 0.373 | 3.61E-222 |
| BLOC1S4 | 4.43E-234 | -0.36495 | 0.042 | 0.364 | 9.76E-230 |
| EGLN3 | 1.40E-86 | -0.36506 | 0.048 | 0.205 | 3.08E-82 |
| ANKRD13D | 3.97E-236 | -0.36508 | 0.054 | 0.39 | 8.74E-232 |
| RPL11 | 5.57E-298 | -0.36543 | 1 | 1 | 1.23E-293 |
| UBE3A | 1.37E-229 | -0.36552 | 0.05 | 0.374 | 3.02E-225 |
| DEGS1 | 6.39E-242 | -0.36553 | 0.066 | 0.42 | 1.41E-237 |
| FBXL17 | 1.01E-203 | -0.36593 | 0.045 | 0.339 | 2.23E-199 |
| U2AF1L4 | 1.15E-227 | -0.36594 | 0.061 | 0.396 | 2.54E-223 |
| LAPTM4A | 2.01E-269 | -0.36617 | 0.093 | 0.5 | 4.42E-265 |
| LGALS8 | 2.74E-152 | -0.36639 | 0.219 | 0.509 | 6.04E-148 |
| RAD23B | 1.31E-234 | -0.36644 | 0.072 | 0.424 | 2.89E-230 |
| OGA | 1.67E-228 | -0.36664 | 0.081 | 0.435 | 3.68E-224 |
| TM2D1 | 5.20E-251 | -0.36671 | 0.066 | 0.428 | 1.15E-246 |
| CITED2 | 3.92E-186 | -0.36728 | 0.139 | 0.467 | 8.62E-182 |
| TEX264 | 1.75E-238 | -0.36759 | 0.052 | 0.389 | 3.86E-234 |
| RPL28 | 0 | -0.36767 | 1 | 1 | 0 |
| PCNX1 | 8.24E-226 | -0.36794 | 0.076 | 0.423 | 1.81E-221 |
| RPS6KA5 | 2.02E-207 | -0.36855 | 0.074 | 0.399 | 4.45E-203 |
| CRTAP | 2.84E-226 | -0.36952 | 0.077 | 0.426 | 6.25E-222 |
| TMED2 | 2.42E-251 | -0.36956 | 0.079 | 0.455 | 5.32E-247 |
| ARHGAP25 | 8.18E-204 | -0.36971 | 0.117 | 0.447 | 1.80E-199 |
| TMEM204 | 4.28E-224 | -0.36976 | 0.036 | 0.342 | 9.42E-220 |
| TSPAN14 | 2.90E-238 | -0.37046 | 0.065 | 0.415 | 6.39E-234 |
| MKLN1 | 1.16E-220 | -0.37085 | 0.106 | 0.461 | 2.55E-216 |
| LINC01619 | 9.44E-191 | -0.37099 | 0.04 | 0.317 | 2.08E-186 |
| PDCD6IP | 3.00E-254 | -0.37128 | 0.075 | 0.449 | 6.60E-250 |
| CEP350 | 7.89E-229 | -0.37203 | 0.088 | 0.448 | 1.74E-224 |
| PAPOLA | 4.99E-252 | -0.37243 | 0.087 | 0.47 | 1.10E-247 |
| MALT1 | 1.13E-212 | -0.3725 | 0.044 | 0.347 | 2.49E-208 |
| GK | 2.45E-201 | -0.37256 | 0.064 | 0.374 | 5.39E-197 |
| LINC01550 | 7.48E-211 | -0.37311 | 0.051 | 0.358 | 1.65E-206 |
| LIMD2 | 1.12E-134 | -0.37339 | 0.999 | 0.964 | 2.47E-130 |
| CEP85L | 5.91E-227 | -0.37408 | 0.065 | 0.403 | 1.30E-222 |
| RPL34 | 4.12E-281 | -0.37412 | 1 | 1 | 9.07E-277 |
| CSGALNACT1 | 1.51E-191 | -0.37413 | 0.077 | 0.388 | 3.32E-187 |
| FXYD5 | 2.65E-164 | -0.37574 | 0.999 | 0.994 | 5.83E-160 |
| GLCCI1 | 7.76E-207 | -0.37575 | 0.08 | 0.411 | 1.71E-202 |
| TTC3 | 8.15E-225 | -0.376 | 0.085 | 0.438 | 1.80E-220 |
| ZFAND6 | 8.66E-255 | -0.37698 | 0.07 | 0.44 | 1.91E-250 |
| SLC35D2 | 1.98E-232 | -0.37718 | 0.037 | 0.353 | 4.35E-228 |
| CLK1 | 1.29E-239 | -0.37734 | 0.059 | 0.404 | 2.83E-235 |
| URI1 | 1.52E-248 | -0.37768 | 0.083 | 0.459 | 3.34E-244 |
| B2M | 0 | -0.37769 | 1 | 1 | 0 |
| MLLT3 | 1.31E-219 | -0.37779 | 0.051 | 0.367 | 2.88E-215 |
| NCK2 | 2.46E-248 | -0.37842 | 0.055 | 0.404 | 5.43E-244 |
| DGKA | 9.96E-204 | -0.37856 | 0.141 | 0.48 | 2.19E-199 |
| IL6ST | 3.30E-186 | -0.3788 | 0.024 | 0.279 | 7.27E-182 |
| CD52 | 2.89E-136 | -0.37996 | 0.999 | 0.988 | 6.36E-132 |
| SERPINB9 | 3.21E-203 | -0.38072 | 0.031 | 0.312 | 7.07E-199 |
| ZNF524 | 7.09E-258 | -0.38151 | 0.067 | 0.436 | 1.56E-253 |
| OXLD1 | 2.89E-259 | -0.38269 | 0.064 | 0.432 | 6.36E-255 |
| CASK | 7.23E-139 | -0.38272 | 0.237 | 0.5 | 1.59E-134 |
| RETREG1 | 2.87E-203 | -0.38335 | 0.033 | 0.315 | 6.32E-199 |
| RBBP6 | 3.31E-246 | -0.38393 | 0.096 | 0.472 | 7.29E-242 |
| TRG-AS1 | 4.53E-235 | -0.38465 | 0.052 | 0.386 | 9.98E-231 |
| PCNX2 | 4.58E-180 | -0.38565 | 0.165 | 0.48 | 1.01E-175 |
| CCNG1 | 3.90E-223 | -0.38598 | 0.064 | 0.397 | 8.60E-219 |
| CNOT7 | 6.96E-262 | -0.386 | 0.061 | 0.429 | 1.53E-257 |
| PURA | 4.07E-246 | -0.38607 | 0.068 | 0.427 | 8.96E-242 |
| GPRIN3 | 3.65E-211 | -0.38628 | 0.081 | 0.416 | 8.04E-207 |
| SLAMF1 | 7.36E-233 | -0.38747 | 0.079 | 0.437 | 1.62E-228 |
| PFKFB3 | 5.15E-220 | -0.38758 | 0.076 | 0.417 | 1.13E-215 |
| CCDC107 | 7.21E-260 | -0.38813 | 0.093 | 0.489 | 1.59E-255 |
| PIAS1 | 2.11E-256 | -0.38934 | 0.074 | 0.449 | 4.65E-252 |
| ZMYM2 | 3.51E-242 | -0.38935 | 0.062 | 0.412 | 7.74E-238 |
| HERC1 | 3.02E-230 | -0.39052 | 0.061 | 0.398 | 6.65E-226 |
| MBD5 | 2.26E-237 | -0.39094 | 0.07 | 0.422 | 4.98E-233 |
| TBCC | 1.39E-245 | -0.39098 | 0.042 | 0.376 | 3.07E-241 |
| CHST11 | 1.35E-207 | -0.39198 | 0.113 | 0.443 | 2.98E-203 |
| CD40LG | 5.70E-201 | -0.3925 | 0.093 | 0.427 | 1.26E-196 |
| DBP | 6.99E-236 | -0.39266 | 0.03 | 0.343 | 1.54E-231 |
| TNFAIP3 | 3.75E-203 | -0.39299 | 0.109 | 0.458 | 8.26E-199 |
| PHC3 | 9.14E-259 | -0.3933 | 0.075 | 0.453 | 2.01E-254 |
| SFXN1 | 1.37E-251 | -0.39338 | 0.077 | 0.45 | 3.01E-247 |
| TSC22D3 | 7.98E-202 | -0.39384 | 0.103 | 0.447 | 1.76E-197 |
| CD69 | 4.32E-199 | -0.39412 | 0.076 | 0.392 | 9.51E-195 |
| BTG1 | 2.80E-142 | -0.39443 | 1 | 0.989 | 6.15E-138 |
| FKBP11 | 1.90E-214 | -0.39448 | 0.074 | 0.407 | 4.18E-210 |
| CDK17 | 1.34E-235 | -0.39478 | 0.069 | 0.419 | 2.95E-231 |
| RAB33A | 1.16E-236 | -0.39485 | 0.074 | 0.428 | 2.54E-232 |
| PPP3CC | 1.38E-250 | -0.39588 | 0.055 | 0.406 | 3.03E-246 |
| HLA-F | 6.29E-238 | -0.39747 | 0.056 | 0.396 | 1.38E-233 |
| IL10RA | 2.94E-251 | -0.3977 | 0.081 | 0.46 | 6.47E-247 |
| TNKS2 | 1.45E-247 | -0.39781 | 0.06 | 0.413 | 3.19E-243 |
| RPS6KA3 | 1.41E-246 | -0.39802 | 0.074 | 0.439 | 3.10E-242 |
| TMX4 | 4.59E-243 | -0.39823 | 0.068 | 0.424 | 1.01E-238 |
| USP25 | 2.92E-236 | -0.39827 | 0.055 | 0.392 | 6.44E-232 |
| MOB4 | 6.60E-270 | -0.3985 | 0.068 | 0.448 | 1.45E-265 |
| MPC1 | 1.61E-255 | -0.39857 | 0.087 | 0.474 | 3.54E-251 |
| CLCN3 | 9.73E-240 | -0.39902 | 0.062 | 0.41 | 2.14E-235 |
| RPL19 | 0 | -0.39955 | 1 | 1 | 0 |
| YPEL5 | 7.16E-278 | -0.40063 | 0.104 | 0.52 | 1.58E-273 |
| RBM38 | 3.98E-217 | -0.40068 | 0.055 | 0.373 | 8.75E-213 |
| PRKCQ-AS1 | 1.56E-230 | -0.40101 | 0.065 | 0.407 | 3.43E-226 |
| PRKACB | 9.39E-242 | -0.40109 | 0.096 | 0.464 | 2.07E-237 |
| CCL5 | 9.83E-261 | -0.40132 | 0.994 | 0.384 | 2.17E-256 |
| UTRN | 1.09E-232 | -0.40207 | 0.074 | 0.423 | 2.39E-228 |
| GIMAP2 | 6.75E-248 | -0.40216 | 0.051 | 0.395 | 1.49E-243 |
| AKT3 | 9.20E-249 | -0.40246 | 0.081 | 0.457 | 2.02E-244 |
| CNIH1 | 3.70E-260 | -0.40343 | 0.069 | 0.442 | 8.16E-256 |
| UBL7 | 5.52E-268 | -0.40443 | 0.064 | 0.44 | 1.22E-263 |
| DNAJB14 | 1.32E-270 | -0.40486 | 0.08 | 0.475 | 2.91E-266 |
| PNPLA2 | 3.28E-271 | -0.4049 | 0.069 | 0.453 | 7.23E-267 |
| ARL2BP | 2.48E-276 | -0.40536 | 0.069 | 0.459 | 5.47E-272 |
| CCNH | 4.18E-248 | -0.40582 | 0.08 | 0.451 | 9.20E-244 |
| PDE4DIP | 1.14E-224 | -0.40626 | 0.059 | 0.389 | 2.52E-220 |
| PDCD4 | 6.61E-88 | -0.40677 | 0.993 | 0.823 | 1.46E-83 |
| CCNL1 | 3.18E-258 | -0.4072 | 0.062 | 0.427 | 7.00E-254 |
| SLC9A9 | 2.86E-207 | -0.4076 | 0.066 | 0.383 | 6.30E-203 |
| ASF1A | 1.64E-252 | -0.4078 | 0.077 | 0.452 | 3.61E-248 |
| PPCS | 4.33E-277 | -0.40786 | 0.071 | 0.462 | 9.54E-273 |
| ASB2 | 1.08E-180 | -0.40858 | 0.058 | 0.338 | 2.39E-176 |
| ZBTB38 | 6.18E-229 | -0.40866 | 0.092 | 0.454 | 1.36E-224 |
| TMEM248 | 5.22E-271 | -0.40882 | 0.074 | 0.461 | 1.15E-266 |
| HPCAL1 | 4.51E-259 | -0.4089 | 0.057 | 0.417 | 9.93E-255 |
| CASP4 | 3.60E-264 | -0.40899 | 0.07 | 0.449 | 7.93E-260 |
| AP3S1 | 3.03E-286 | -0.40918 | 0.08 | 0.489 | 6.67E-282 |
| TTC17 | 2.62E-251 | -0.41022 | 0.055 | 0.407 | 5.76E-247 |
| TRAF1 | 6.10E-257 | -0.41113 | 0.073 | 0.446 | 1.34E-252 |
| MTERF4 | 5.42E-253 | -0.41181 | 0.047 | 0.392 | 1.19E-248 |
| GATA3 | 2.13E-214 | -0.41183 | 0.084 | 0.425 | 4.69E-210 |
| STT3B | 5.55E-265 | -0.41342 | 0.06 | 0.429 | 1.22E-260 |
| CHIC2 | 2.13E-276 | -0.41474 | 0.062 | 0.443 | 4.70E-272 |
| ZNF638 | 8.20E-267 | -0.41533 | 0.072 | 0.454 | 1.81E-262 |
| HNRNPLL | 1.89E-229 | -0.4167 | 0.113 | 0.472 | 4.17E-225 |
| FNBP4 | 4.15E-269 | -0.41746 | 0.075 | 0.463 | 9.13E-265 |
| PHTF2 | 1.78E-241 | -0.41803 | 0.098 | 0.477 | 3.92E-237 |
| RPS27A | 0 | -0.4229 | 1 | 1 | 0 |
| TUBA4A | 7.39E-250 | -0.42403 | 0.057 | 0.408 | 1.63E-245 |
| GTF2B | 2.17E-297 | -0.42426 | 0.07 | 0.477 | 4.79E-293 |
| LMBRD1 | 6.63E-287 | -0.42473 | 0.07 | 0.47 | 1.46E-282 |
| WSB1 | 8.83E-259 | -0.42529 | 0.078 | 0.456 | 1.94E-254 |
| SARAF | 4.30E-226 | -0.42611 | 0.999 | 0.979 | 9.47E-222 |
| METTL23 | 7.19E-290 | -0.42829 | 0.08 | 0.492 | 1.58E-285 |
| OXNAD1 | 9.68E-201 | -0.4291 | 0.164 | 0.497 | 2.13E-196 |
| GOLGA7 | 4.04E-278 | -0.42993 | 0.06 | 0.44 | 8.89E-274 |
| RPL26 | 0 | -0.43166 | 1 | 1 | 0 |
| RPL7A | 0 | -0.43234 | 1 | 1 | 0 |
| NLRC3 | 3.22E-262 | -0.4338 | 0.079 | 0.457 | 7.09E-258 |
| ATP6V0E2 | 1.66E-286 | -0.4342 | 0.074 | 0.469 | 3.64E-282 |
| RPS23 | 0 | -0.43621 | 1 | 1 | 0 |
| SERINC5 | 1.61E-243 | -0.43713 | 0.083 | 0.446 | 3.54E-239 |
| ZNF292 | 5.31E-267 | -0.43726 | 0.086 | 0.469 | 1.17E-262 |
| EGLN2 | 2.61E-298 | -0.43766 | 0.071 | 0.482 | 5.75E-294 |
| MT-ATP6 | 2.03E-197 | -0.43891 | 1 | 0.998 | 4.47E-193 |
| TBRG1 | 4.42E-284 | -0.43931 | 0.056 | 0.439 | 9.74E-280 |
| PLEKHF1 | 8.65E-275 | -0.4397 | 0.054 | 0.426 | 1.91E-270 |
| RPS14 | 0 | -0.44051 | 1 | 1 | 0 |
| SIRPG | 9.79E-246 | -0.44074 | 0.057 | 0.406 | 2.16E-241 |
| SDCBP | 2.44E-269 | -0.44269 | 0.1 | 0.5 | 5.38E-265 |
| FAM102A | 2.04E-281 | -0.44302 | 0.065 | 0.454 | 4.49E-277 |
| FXYD7 | 1.49E-153 | -0.44344 | 0.027 | 0.25 | 3.27E-149 |
| LAG3 | 9.89E-103 | -0.44427 | 0.079 | 0.281 | 2.18E-98 |
| TIGIT | 1.08E-126 | -0.44477 | 0.053 | 0.265 | 2.39E-122 |
| GRAMD1A | 1.67E-298 | -0.44675 | 0.068 | 0.476 | 3.68E-294 |
| RPS3A | 0 | -0.44865 | 1 | 1 | 0 |
| NKTR | 1.48E-281 | -0.44957 | 0.061 | 0.444 | 3.25E-277 |
| FGFR1OP2 | 1.08E-303 | -0.45182 | 0.065 | 0.474 | 2.37E-299 |
| HSD17B11 | 2.46E-288 | -0.45191 | 0.061 | 0.453 | 5.42E-284 |
| CDC42EP3 | 4.44E-286 | -0.45202 | 0.069 | 0.466 | 9.78E-282 |
| SCML4 | 4.67E-253 | -0.4559 | 0.032 | 0.362 | 1.03E-248 |
| NELL2 | 9.80E-173 | -0.45773 | 0.045 | 0.305 | 2.16E-168 |
| RAPGEF6 | 1.67E-278 | -0.46264 | 0.066 | 0.453 | 3.68E-274 |
| MALAT1 | 8.77E-181 | -0.46668 | 1 | 1 | 1.93E-176 |
| RPS4X | 0 | -0.46676 | 1 | 1 | 0 |
| ZNRF2 | 6.57E-274 | -0.46843 | 0.104 | 0.488 | 1.45E-269 |
| TRAT1 | 1.24E-283 | -0.46856 | 0.068 | 0.462 | 2.74E-279 |
| LYSMD2 | 1.89E-293 | -0.47135 | 0.073 | 0.48 | 4.16E-289 |
| HIVEP2 | 1.12E-280 | -0.47376 | 0.067 | 0.456 | 2.46E-276 |
| RPL41 | 0 | -0.47617 | 1 | 1 | 0 |
| ID3 | 9.86E-174 | -0.47974 | 0.044 | 0.304 | 2.17E-169 |
| PSMB8-AS1 | 0 | -0.48925 | 0.072 | 0.503 | 0 |
| RPS19 | 0 | -0.48952 | 1 | 1 | 0 |
| RPL13 | 0 | -0.49544 | 1 | 1 | 0 |
| SORL1 | 3.79E-275 | -0.49733 | 0.044 | 0.405 | 8.35E-271 |
| CCDC18-AS1 | 2.51E-304 | -0.50387 | 0.06 | 0.464 | 5.53E-300 |
| RPS15A | 0 | -0.50399 | 1 | 1 | 0 |
| ERN1 | 1.97E-279 | -0.50579 | 0.062 | 0.446 | 4.34E-275 |
| MT-CO1 | 0 | -0.51371 | 1 | 1 | 0 |
| RPS12 | 0 | -0.5152 | 1 | 1 | 0 |
| TSHZ2 | 4.60E-93 | -0.5163 | 0.217 | 0.405 | 1.01E-88 |
| RPL30 | 0 | -0.52173 | 1 | 1 | 0 |
| FBLN7 | 2.44E-280 | -0.52293 | 0.054 | 0.431 | 5.37E-276 |
| TMSB4X | 0 | -0.52561 | 1 | 1 | 0 |
| MT-CO3 | 0 | -0.54195 | 1 | 0.997 | 0 |
| RPL3 | 0 | -0.54552 | 1 | 1 | 0 |
| SESN3 | 1.71E-242 | -0.54846 | 0.061 | 0.405 | 3.77E-238 |
| PASK | 5.57E-185 | -0.56499 | 0.029 | 0.287 | 1.23E-180 |
| RPS18 | 0 | -0.56645 | 1 | 1 | 0 |
| RPL10 | 0 | -0.56763 | 1 | 1 | 0 |
| CXCR6 | 6.50E-45 | -0.58618 | 0.319 | 0.414 | 1.43E-40 |
| LTB | 2.44E-15 | -0.58946 | 0.99 | 0.695 | 5.38E-11 |
| RPS3 | 0 | -0.61477 | 1 | 1 | 0 |
| EEF1A1 | 0 | -0.63889 | 1 | 1 | 0 |
| MT-ND3 | 0 | -0.66864 | 1 | 0.997 | 0 |
| IL7R | 3.12E-49 | -0.7542 | 0.997 | 0.824 | 6.86E-45 |
| MT-ATP8 | 0 | -0.79407 | 0.998 | 0.99 | 0 |
| KLRB1 | 7.93E-135 | -0.85565 | 0.12 | 0.357 | 1.75E-130 |
| TRBV5-1 | 3.88E-12 | -0.95095 | 0.069 | 0.107 | 8.55E-08 |
| TRBV20-1 | 1.39E-10 | -0.99662 | 0.095 | 0.131 | 3.05E-06 |
| MT-CO2 | 0 | -1.01215 | 1 | 0.999 | 0 |
