# Supplementary material for: Effect of fentanyl on HIV expression in peripheral blood mononuclear cells
Source: Front Microbiol. 2024 Sep 25;15:1463441. doi: 10.3389/fmicb.2024.1463441 (PMC11461324; doi:10.3389/fmicb.2024.1463441)
Supplement: Supplementary file 5 [file Presentation_4.PPTX]

## Slide 1
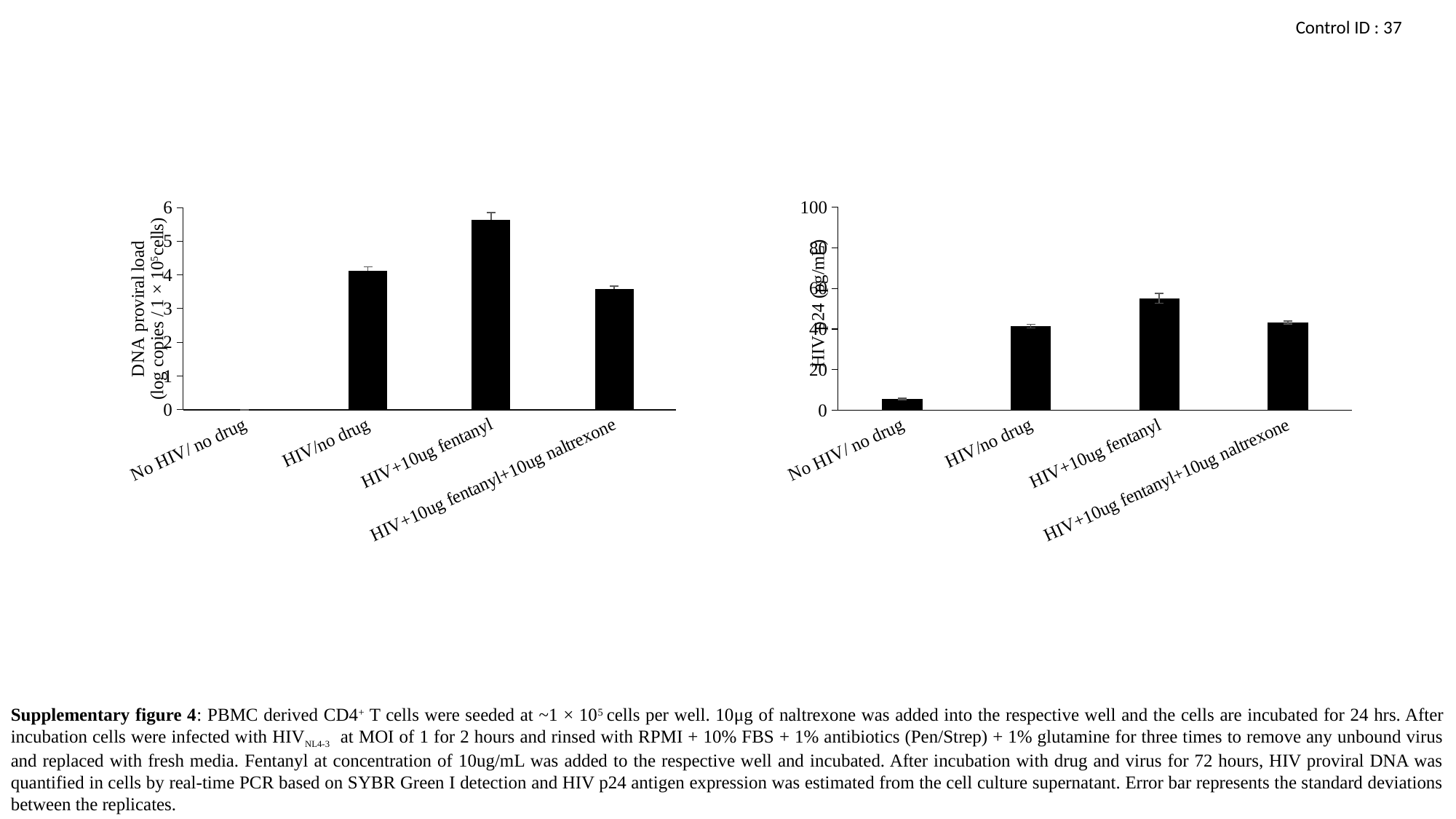

Control ID : 37
### Chart
| Category | |
|---|---|
| No HIV/ no drug | 0.0 |
| HIV/no drug | 4.128484370241138 |
| HIV+10ug fentanyl | 5.64008561778008 |
| HIV+10ug fentanyl+10ug naltrexone | 3.5763993345750134 |
### Chart
| Category | |
|---|---|
| No HIV/ no drug | 5.502938241463694 |
| HIV/no drug | 41.472234270939055 |
| HIV+10ug fentanyl | 55.174823234548725 |
| HIV+10ug fentanyl+10ug naltrexone | 43.185057891390265 |Supplementary figure 4: PBMC derived CD4+ T cells were seeded at ~1 × 10^5^ cells per well. 10μg of naltrexone was added into the respective well and the cells are incubated for 24 hrs. After incubation cells were infected with HIVNL4-3 at MOI of 1 for 2 hours and rinsed with RPMI + 10% FBS + 1% antibiotics (Pen/Strep) + 1% glutamine for three times to remove any unbound virus and replaced with fresh media. Fentanyl at concentration of 10ug/mL was added to the respective well and incubated. After incubation with drug and virus for 72 hours, HIV proviral DNA was quantified in cells by real-time PCR based on SYBR Green I detection and HIV p24 antigen expression was estimated from the cell culture supernatant. Error bar represents the standard deviations between the replicates.
