# Supplementary material for: Effect of fentanyl on HIV expression in peripheral blood mononuclear cells
Source: Front Microbiol. 2024 Sep 25;15:1463441. doi: 10.3389/fmicb.2024.1463441 (PMC11461324; doi:10.3389/fmicb.2024.1463441)
Supplement: Supplementary file 6 [file Presentation_5.PPTX]

## Slide 1
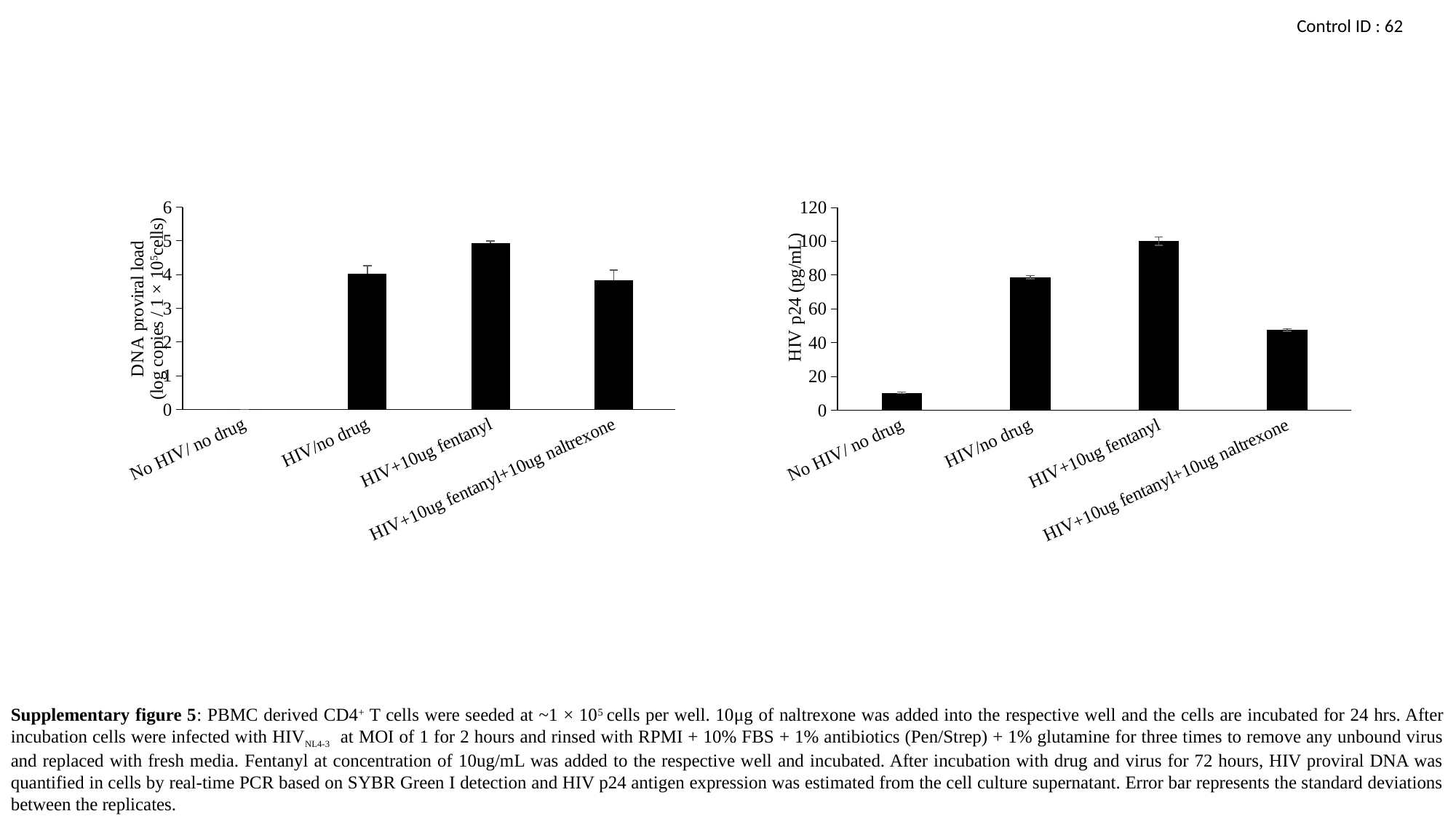

Control ID : 62
### Chart
| Category | |
|---|---|
| No HIV/ no drug | 0.0 |
| HIV/no drug | 4.036733396064358 |
| HIV+10ug fentanyl | 4.939082908019804 |
| HIV+10ug fentanyl+10ug naltrexone | 3.839334570382821 |
### Chart
| Category | |
|---|---|
| No HIV/ no drug | 10.377897776594054 |
| HIV/no drug | 78.75908693153073 |
| HIV+10ug fentanyl | 100.10350435561503 |
| HIV+10ug fentanyl+10ug naltrexone | 47.5329947740741 |Supplementary figure 5: PBMC derived CD4+ T cells were seeded at ~1 × 10^5^ cells per well. 10μg of naltrexone was added into the respective well and the cells are incubated for 24 hrs. After incubation cells were infected with HIVNL4-3 at MOI of 1 for 2 hours and rinsed with RPMI + 10% FBS + 1% antibiotics (Pen/Strep) + 1% glutamine for three times to remove any unbound virus and replaced with fresh media. Fentanyl at concentration of 10ug/mL was added to the respective well and incubated. After incubation with drug and virus for 72 hours, HIV proviral DNA was quantified in cells by real-time PCR based on SYBR Green I detection and HIV p24 antigen expression was estimated from the cell culture supernatant. Error bar represents the standard deviations between the replicates.
