# Supplementary material for: Effect of fentanyl on HIV expression in peripheral blood mononuclear cells
Source: Front Microbiol. 2024 Sep 25;15:1463441. doi: 10.3389/fmicb.2024.1463441 (PMC11461324; doi:10.3389/fmicb.2024.1463441)
Supplement: Supplementary file 7 [file Presentation_6.PPTX]

## Slide 1
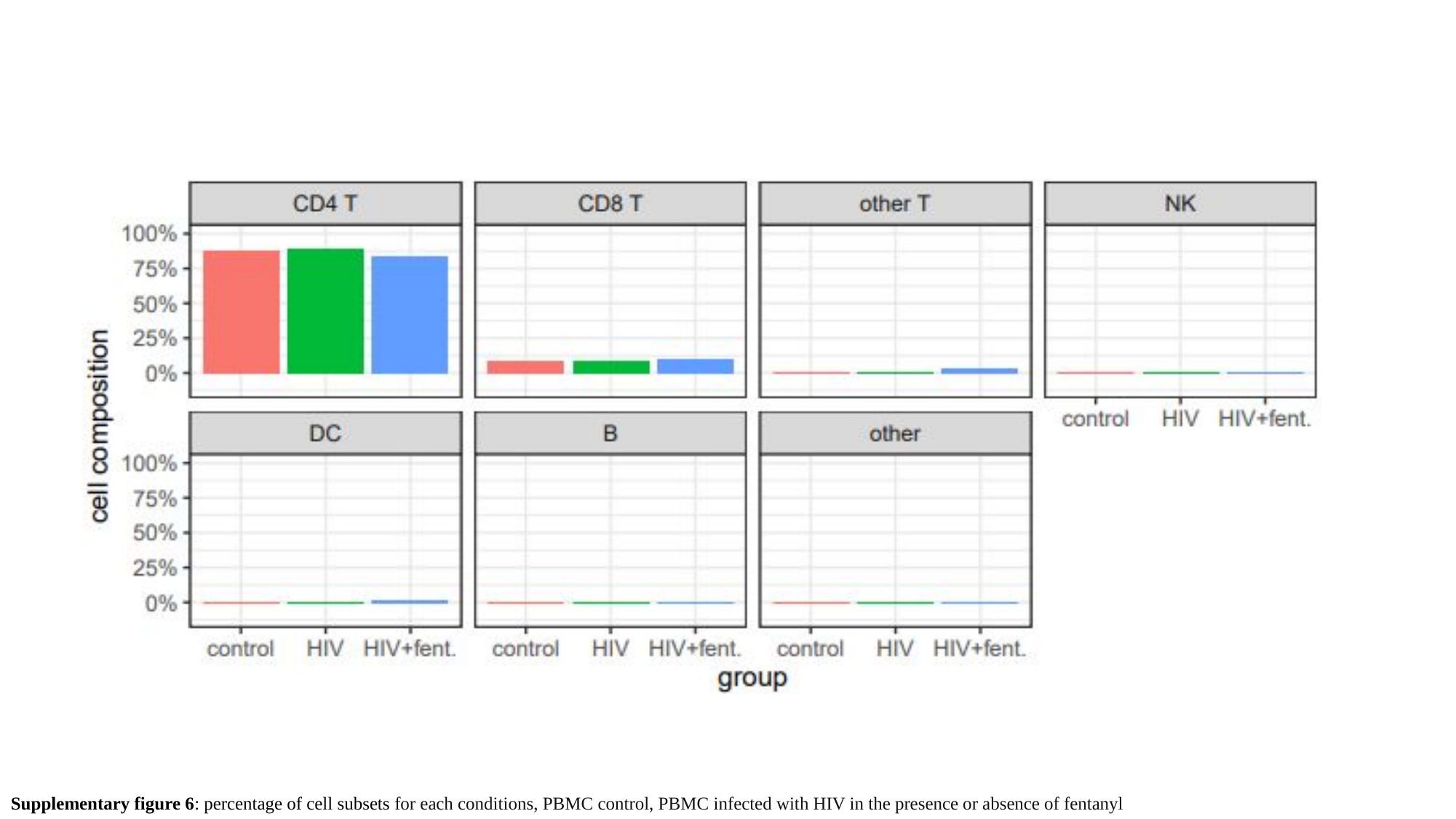

Supplementary figure 6: percentage of cell subsets for each conditions, PBMC control, PBMC infected with HIV in the presence or absence of fentanyl
