# Supplementary material for: Effect of fentanyl on HIV expression in peripheral blood mononuclear cells
Source: Front Microbiol. 2024 Sep 25;15:1463441. doi: 10.3389/fmicb.2024.1463441 (PMC11461324; doi:10.3389/fmicb.2024.1463441)
Supplement: Supplementary file 8 [file Presentation_7.PPTX]

## Slide 1
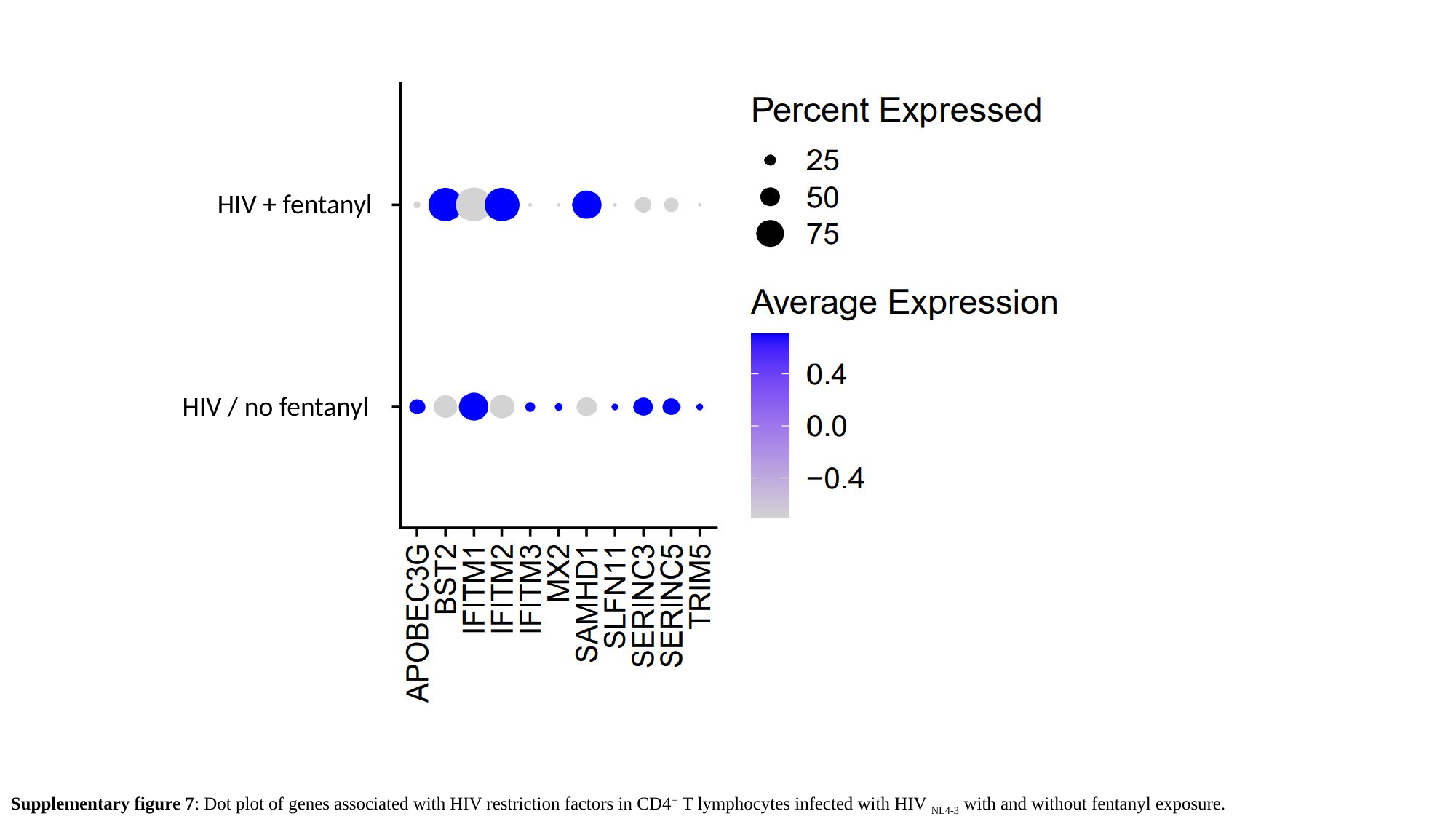

HIV + fentanyl
HIV / no fentanyl
Supplementary figure 7: Dot plot of genes associated with HIV restriction factors in CD4+ T lymphocytes infected with HIV NL4-3 with and without fentanyl exposure.
